# Scarring and selection effects on children surviving elevated rates of postneonatal mortality in sub-Saharan Africa

**DOI:** 10.1016/j.ssmph.2022.101160

**Published:** 2022-07-02

**Authors:** Omar Karlsson

**Affiliations:** a Takemi Program in International Health, Harvard T.H. Chan School of Public Health, Harvard University, 677 Huntington Avenue, Boston, MA, 02115, United States; b Department of Economic History, School of Economics and Management, Lund University, P.O. Box 7083, 220 07 Lund, Sweden

**Keywords:** Height-for-age, School attendance, Early life adversity, Scarring, Selective mortality, Postneonatal mortality rate

## Abstract

Infants in sub-Saharan Africa face adversity: Infections and undernutrition are major causes of infant deaths and can cause physiological damage with long-lasting adverse *scarring* effects on the human development of the survivors, for example, in terms of health and education. However, *selective mortality* of more vulnerable children at very high levels of adversity in early life can leave the surviving population to appear on average healthier. This paper estimated the nonlinear effects of postneonatal mortality rate—a proxy for adversity, particularly infections and undernutrition—in a 50 km radius, occurring over the period of infancy, on the subsequent height-for-age and school attendance of the surviving children. The results indicated that an adverse environment in infancy negatively affected height-for-age at age 1–4 years: At relatively low levels of adversity (at the 10th percentile of postneonatal mortality rate), an additional postneonatal death per 100 person-years decreased height-for-age of the survivors by almost 2% of the mean deficit in height (relative to a common growth standard) when comparing siblings born into different levels of adversity. At high levels of adversity, no effect was found for height-for-age while a small positive association was observed for school attendance at age 7–16 years. The results indicated that selective mortality may have canceled out (or even dominated in the case of school attendance) observable scarring effects following high levels of postneonatal mortality rate in sub-Saharan Africa.

## Highlights

• Adversity in early life negatively affects human development in sub-Saharan Africa.
• The postneonatal mortality rate over infancy was used as a proxy for adversity.
• Elevated adversity in infancy negatively affected height-for-age at age 1–4 years.
• At high adversity, selective mortality may cancel out observable scarring effects.

## 1 Introduction

In sub-Saharan Africa, over 750 thousand children die in the postneonatal period, mostly due to adverse exposures such as undernutrition and infections causing diarrhea, pneumonia, and malaria (Global Burden of Disease Collaborative Network, 2020). Such adversity also has long-term negative consequences for the human development of surviving children, for example, health and cognitive development, which is reflected in reduced physical growth and eventually shorter adult height and lower education, income, and health (Case & Paxson, 2008; Crimmins & Finch, 2006; McGovern et al., 2017).

Negative *scarring effects* of early life adversity on adult height, earnings, education, and health have been identified using child mortality rates as proxies for adversity (Bengtsson & Lindström, 2003; Bozzoli et al., 2009). This paper studied the effects of adversity in infancy on survivors’ height-for-age at age 1–4 years and school attendance at age 7–16 years in sub-Saharan Africa by comparing siblings exposed to different rates of postneonatal mortality within a 50 km radius in infancy.

In addition to *scarring effects,* adversity can also cause *selection effects* wherein those more vulnerable and on a worse health trajectory—for example, due to poverty and underlying health problems—are more likely to die during periods of elevated adversity, leaving the surviving population to appear “healthier” (Bozzoli et al., 2009; Preston et al., 1998). A selection effect is suggested to dominate over scarring at extreme levels of adversity, such as in many areas in sub-Saharan Africa, where adversity in early life was found to positively affect adult height (Deaton, 2007). Further, adults in sub-Saharan Africa are relatively tall while prevalence of stunted growth in childhood is high, suggesting that adult height may not adequately reflect scarring effects of childhood morbidity (Akachi & Canning, 2010).

Therefore, this paper's first contribution was to assess the nonlinear effects of adversity in infancy on outcomes observable early in the human development process, before adulthood. The second contribution was a new proxy for temporal variation in the intensity of adversity which was exogenous to child health outcomes and more period and area-specific, which provided more variation than the annual national-level proxies used in previous studies. Further, contextual covariates constructed from household surveys have proved helpful in studying health more generally (Karlsson, 2019; Kravdal, 2004): this paper extended the use of contextual information by taking advantage of the spatial dimension of geocoded neighborhoods as well as the temporal dimension of birth histories.

## 2 Background

### 2.1 Ecological measures of adversity

Children reported to have suffered respiratory infections or other severe illnesses in early life have been observed to be as much as 2.5 cm shorter later in childhood (Kuh & Wadsworth, 1989; Rona & Florey, 1980; Tanner, 1962). However, disease history in early childhood may be endogenous to later outcomes (Johansson, 2004). Ecological indicators indexed to the year of birth (or other periods in early life) provide variation approximating adversity that is exogenous to later health outcomes. For example, temporal variations in malaria rates in early life negatively affected adulthood socioeconomic status and productivity (Barreca, 2010; Bleakley, 2010; Cutler et al., 2010; Lucas, 2010). Mortality rates are also common proxies for the extent of adversity (Bozzoli et al., 2009): A high infant mortality rate in the year of birth has been found to reduce adult life expectancy (Finch & Crimmins, 2004) and wealth accumulation (Bengtsson & Broström, 2009). The effects of infant mortality rate indexed to later periods in childhood were not as strong, indicating that harmful exposures in infancy were more critical (Finch & Crimmins, 2004).

### 2.2 Selection and scarring

Four mechanisms link early life adversity to later outcomes: scarring and acquired immunity are direct effects, and correlated environments and selection are indirect effects (Preston et al., 1998). Scarring refers to the long-term negative effects of adversity in early life on survivors, which shifts the whole distribution of health downwards. On the other hand, better adult health is a possible consequence of exposure to infections that provide future immunity. Acquired immunity may, however, be more relevant for adult mortality than height (Preston et al., 1998).

Children exposed to certain types of adversity in early life are often more likely to be exposed to other types, as well as continuing to suffer adversity in later periods because their environment—in terms of living standards or external epidemiological environment—often is correlated throughout their life (Preston et al., 1998) and across generations (Subramanian et al., 2009). Not accounting for correlated environments—for example, by comparing individuals within the same context—would bias the observed effect between ecological measures of early life adversity and later health outcomes upwards.

Finally, in some cases, the observed association between adverse environment in infancy and later outcomes can be biased downwards, canceled out, or in the most extreme situations, be positive because of selective mortality: If those with lower potential health—for example due to poverty and underlying health problems—are more likely than those on a better health trajectory to die during an increase in harmful exposures in early life—such as a famine or an epidemic—the observed mean health of the surviving population may be greater. Selection has been suggested to dominate at high levels of adversity, such as those existing in sub-Saharan Africa (Bozzoli et al., 2009). For example, survivors of the 1959–61 famine in China exposed in childhood were 1–2 cm taller as adults than those not exposed (Gørgens et al., 2012). Similarly, for women in sub-Saharan Africa, there was a positive association between child mortality rate during the year of birth and adult height, which suggests that selection dominated over scarring (Deaton, 2007).

### 2.3 Type of adversity reflected in postneonatal deaths

In sub-Saharan Africa, in 2019, deaths at 1–11 months were mainly attributed to diarrheal diseases (22%), lower respiratory infections (21.1%), malaria (15%), meningitis (4.5%), protein-energy malnutrition (3.8%), and whooping cough (3.8%; Supplementary Table S1). Further, these have been the top six causes of postneonatal deaths over the past 30 years (although measles were more common than whooping cough in 2000 and 1990). These causes were common across sub-Saharan Africa subregions (Global Burden of Disease Collaborative Network, 2020).

Although deaths are attributed to specific causes, most deaths result from repeated infections and chronic undernutrition, which also operate in synergy: infections inhibit the uptake of nutrients and suppress appetite, and undernutrition increases susceptibility and mortality from infections (Katona & Katona-Apte, 2008). Estimates have indicated that 45% of child deaths were related to child undernutrition (Black et al., 2013), for example, 73% of diarrhea deaths and 44% of pneumonia deaths (WHO, 2009).

Therefore, the postneonatal mortality rate is expected to mostly reflect undernutrition and infections causing diarrhea, pneumonia, and malaria, which are all significant determinants of stunted growth (Schlaudecker et al., 2011). Further, postneonatal mortality is a better indicator of undernutrition and disease environment facing infants than deaths occurring in the neonatal period (Bozzoli et al., 2009): over 50% of deaths among neonates are attributed to neonatal encephalopathy due to birth asphyxia and preterm birth (Global Burden of Disease Collaborative Network, 2020).

### 2.4 Variations by socioeconomic status and sex

Child health varies across socioeconomic status in most contexts, whether measured by the mother's education (Karlsson et al., 2018), father's occupation (Dribe & Karlsson, 2022), household wealth (Karlsson et al., 2020), or other measures. As for interactions with early life adversity, households with greater socioeconomic status could have invested more in the health of their children pre-adversity (eg, making them less susceptible to infections), sheltered them during periods of adversity (eg, by preventing and responding more effectively to infections), or by increasing post-exposure investments to ease some of the negative impacts of adversity.

Scarring or selection mechanisms may be greater for males, as males generally have higher mortality and stunting rates, most likely due to biological frailty (Sawyer, 2012; Wamani et al., 2007).

## 3 Data and methods

### 3.1 Data source

The nationally representative Demographic and Health Surveys were used (DHS, 2022). Women 15–49 years were interviewed, and data were collected on their birth histories, children's health, and other information. Information on the household and its members, such as whether they attended school, was also recorded. Only children born to interviewed women were considered for this study in order or obtain birth-related covariates. All surveys from sub-Saharan Africa that contained the relevant variables were pooled, and most countries had more than one survey.

The Demographic and Health Surveys used two-stage sampling. Primary sampling units were first sampled from strata of subnational regions separated into urban and rural areas with a probability proportional to size and then 20–30 households randomly drawn from each primary sampling unit (ICF International, 2012). The primary sampling units were relatively small geographic units, such as villages or urban neighborhoods (referred to as neighborhoods hereafter), geocoded in most surveys. The GPS locations were displaced to protect anonymity: Urban primary sampling units were displaced between 0 and 2 km, most rural primary sampling units were displaced between 0 and 5 km, and a randomly selected 1% of rural primary sampling units were displaced between 0 and 10 km (Burgert et al., 2013).

### 3.2 Outcomes

Child height-for-age z-scores represent standard deviations from a median growth trajectory of healthy children according to the 2006 World Health Organization growth standards (WHO, 2006). Trained interviewers measured height in millimeters. Age was recorded in months. Lower height-for-age was indicative of growth restrictions due to undernutrition and disease exposures.

The analyses of height-for-age were based on 100 Demographic and Health Surveys (96 when using sibling fixed-effects) from 33 countries (Table 1 and Supplementary Table S2). In most surveys, all children under five were considered for height measures (although in some surveys, only a subsample or children under three were considered). Children under 12 months old were excluded since they had not lived through the risk period (ie, infancy). A total of 444,925 children 1–4 years old were selected for height measure, while 27,326 had either an implausible height-for-age—that is, six z-scores above or below the median (Assaf et al., 2015)—or a missing height measure (Supplementary Table S2).

**Table 1:** Descriptive statistics.

Sample for outcome->	Height-for-age		School attendance	
Sample for fixed-effects->	Neighborhood	Sibling	Neighborhood	Sibling
Variables				
Height-for-age z-score	−1.8	−1.75		
	[-1.81, −1.8]	[-1.77, −1.7]		
School attendance			.761	.759
			[.757, .77]	[.755, .76]
Postneonatal mortality rate (per 100)	5.25	5.1	6.35	6.37
	[5.2, 5.3]	[5.05, 5.2]	[6.31, 6.4]	[6.33, 6.4]
Mother's education (years)	3.29	3.1	3.33	3.1
	[3.24, 3.3]	[3.03, 3.2]	[3.29, 3.4]	[3.05, 3.1]
Mother's age at birth (years)	27.3	27	26	26.4
	[27.3, 27]	[26.9, 27]	[26, 26]	[26.4, 26]
Birth interval (months)	38.4	30.2	35.9	33.7
	[38.3, 39]	[30.1, 30]	[35.8, 36]	[33.6, 34]
Firstborn	.197	.118	.218	.148
	[.195, .2]	[.115, .12]	[.216, .22]	[.147, .15]
Birth order	3.81	3.94	3.46	3.71
	[3.8, 3.8]	[3.91, 4]	[3.45, 3.5]	[3.69, 3.7]
Number of siblings	4.23	4.6	5.91	6.35
	[4.21, 4.2]	[4.57, 4.6]	[5.89, 5.9]	[6.33, 6.4]
Age (months)	34.1	35.3	135	138
	[34.1, 34]	[35.2, 35]	[135, 135]	[138, 138]
Female	.497	.494	.489	.485
	[.494, .5]	[.49, .5]	[.487, .49]	[.483, .49]
Twin	.0294	.0146	.0255	.0156
	[.0284, .03]	[.0136, .016]	[.0249, .026]	[.0152, .016]
Mother had education	.486	.48	.501	.485
	[.48, .49]	[.471, .49]	[.496, .51]	[.479, .49]
Child's year of birth	2004	2004	2000	2000
	[2003, 2004]	[2003, 2004]	[2000, 2000]	[1999, 2000]
Rural resident	.697	.719	.708	.723
	[.69, .7]	[.71, .73]	[.702, .71]	[.716, .73]

Observations	196,543	57,814	441,159	323,830
Fixed-effects	23,257	28,603	25,719	127,515
Surveys	100	96	73	73
Countries	33	33	32	32

Notes: 95% confidence intervals (shown in brackets) were adjusted for clustering within primary sampling units. Descriptive statistics for birth interval excluded firstborns (who were however included in the analyses using dummy variable adjustment). Separate samples were used for each outcome (height-for-age and school attendance) indicated in the first row of each column. Analyses using neighborhood fixed-effects models excluded neighborhoods with a single observation and sibling fixed-effects models excluded children without a sibling with valid data: the same exclusions were applied in this table indicated in the second row of each column. Descriptive statistics without these exclusions are shown in Supplementary Table S6. Postneonatal mortality rate refers to deaths per 100 person-years (ie, deaths per 1200 person months of exposure). Postneonatal mortality rate was restricted to 50 km radius and linked to the period of infancy.

An indicator for whether 7–16-year-olds attended school in the survey year was obtained from the data on household members. Studies have found child health—measured by, for example, height-for-age—to have determined school attendance in Ghana, Tanzania, and Pakistan (Alderman et al., 2001; Brooker et al., 1999). The analyses of school attendance were based on 73 surveys from 32 countries (Table 1 and Supplementary Table S3). A total of 740,671 children 7–16-year-olds could be linked to a mother in the same household (ie, from the household data to the women's data): Thereof, 851 had missing information on school attendance.

Several variables had a small association (but statistically significant in some cases) with being excluded due to missing outcomes: for example, maternal education, which was slightly lower among children with missing information (Supplementary Tables S4–S5).

### 3.3 Constructing postneonatal mortality rate

All birth histories that included coordinates for neighborhoods were pooled, and sums of person-time and deaths used to construct the postneonatal mortality rate did not necessarily occur within the same survey (but were restricted within countries). Surveys that did not record the outcome variables may still have been used to calculate the postneonatal mortality rate. Age 12 months was classified as postneonatal when constructing the mortality rate, due to age heaping on age at death. Distance between neighborhoods was determined using the haversine formula.

The rate (*pnm*_*nb*_) was calculated for each target neighborhood *n* and each target month *b* as:

$${pnm}_{nb}=\frac{\sum_{p=n}^{P}\;\sum_{b}^{b+11}{deaths}_{pm}\;}{\sum_{p=n}^{P}\;\sum_{m=b}^{b+11}{at\;risk}_{pm}}\times 1200$$

${deaths}_{pm}$ refers to the number of children aged 1–12 months that died in each month *m* (ie, any month between when the first child was born and until the month of the most recent survey in the pooled birth histories) and neighborhood *p;* with neighborhoods *p* to *P* being within 50 km of the target neighborhood *n* (and including *n*). ${at\;risk}_{pm}$ refers to the number of children aged 1–12 months under observation (ie, who had entered the age period and were not censored due to having died or past the survey month or lived through the age period) in each month *m* and neighborhood *p*. The numerator was the number of postneonatal deaths (ie, in ages 1–12 months) occurring in all neighborhoods *p* within a 50-km radius from the target neighborhood *n* over the period between the target month *b* and *b+11* (corresponding to a period of infancy). The denominator was the total person-months at risk (ie, alive infants aged 1–12 months) within a 50-km radius from the target neighborhood over the period *b* to *b+11*. The postneonatal mortality rate was multiplied by 1200 so that it indicated the number of deaths for every 100 person-years. The postneonatal mortality rate in each target neighborhood and month was linked to the corresponding neighborhood and month of birth of children with outcome measures in the data.

**Fig. 1:**
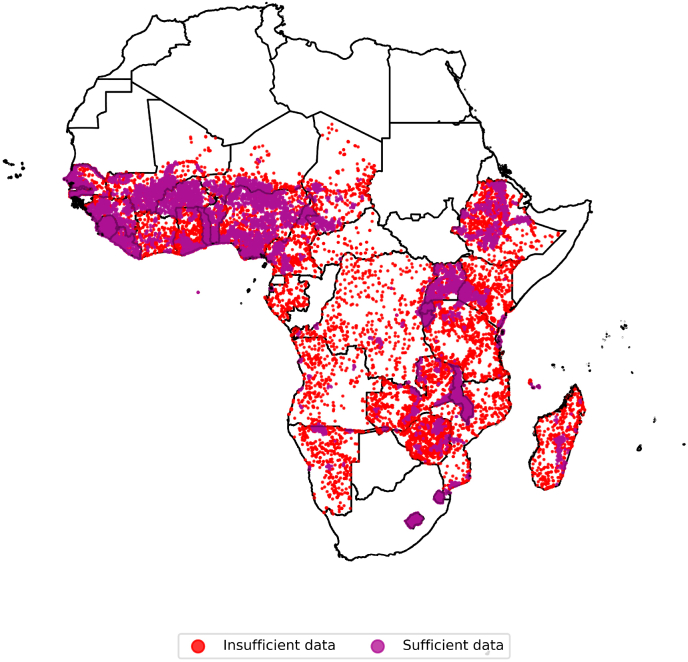
Map of the study area. Notes: Markers indicate neighborhoods (ie, primary sampling units). Insufficient data refers to neighborhoods where no children were exposed to at least 5 postneonatal deaths and 600 person-months over the period of infancy in a 50 km radius and were therefore excluded from analyses. This map refers to the sample used for analyses of height-for-age: a map showing neighborhoods in the analyses of school attendance looks similar and is shown in Supplementary Table S1.

The postneonatal mortality rate was limited to neighborhoods and periods with more than four deaths and 600 person-months (or 50 person-years) of exposure within a 50 km radius to improve precision and reduce noise. A total of 218,182 children with a valid height-for-age were excluded due to this limit (Supplementary Table S2), and 297,415 of the children with a valid measure of school attendance (Supplementary Table S3). These excluded children were more rural and somewhat better-off in terms of maternal education when compared overall, but worse-off when compared within surveys (Supplementary Tables S4–S5). Few neighborhoods had sufficient data to construct postneonatal mortality rates in Central Africa (Fig. 1 and Supplementary Fig. S1). The resulting measures ranged from 0.5 to 34.8 postneonatal deaths per 100 person-years for the pooled sample for both outcomes (Supplementary Fig. S2).

### 3.4 Covariates

Covariates were child's age (in months), birth order, birth interval (in months; adjusting for the firstborns using a dummy variable adjustment), and three binary indicators for whether the child was a firstborn, female, and twin (or triplet, etc.). Maternal education (in years), mother's age at birth (in years), and the number of siblings ever born to mother were also added to models using neighborhood fixed-effects. Squared terms were included for mother's age at birth, since young age can be harmful due to immaturity or low socioeconomic status while advanced age may also be negative for biological reasons (Aldous & Edmonson, 1993; Cabrera, 1980; Fraser et al., 1995); and child's age, since heigh-for-age tends to deviate further from the growth reference as children age until age two, but afterwards plateauing or improving somewhat (De Onis et al., 2006).

Except for the child's age, these covariates were unlikely to reflect confounders. However, not accounting for age could cause effects to be overestimated: older children may have suffered negative exposures over a longer period, which decreased their height-for-age (De Onis et al., 2006), as well as having been exposed to higher rates of postneonatal mortality in infancy on average, due to a downward trend in mortality. Further, as mortality has trended downwards, height-for-age and school attendance have trended upwards across cohorts, which may also bias the estimated effect, if not accounting for age. Bias relating to general improvements in outcomes and exposure was more of a concern when analyzing school attendance since children in that sample were born up to ten years apart, whereas that bias was less of a concern when analyzing the height-for-age sample, since in that sample children were born at most 4 years apart.

### 3.5 Estimation

Sibling fixed-effects models were estimated to account for all households and sibling-level factors. Additionally, neighborhood fixed-effects models were estimated for comparison. The models were specified as ordinary least squares regression models:

$$y_{isnb}=\alpha +\delta_{s}+\beta_{1}{pnm}_{nb}+\beta_{2}{pnm}_{nb}^{2}+\theta^{\prime }x_{i}+\varepsilon_{isnb}$$

where *y* was one of the two outcomes, **x** was a vector of covariates, and $pnm_{nb}$ was the measure for adversity—postneonatal mortality rate—indexed to the month of birth *b* and neighborhood *n* of child *i* in sibling pair *s* (or sibling group in a few cases). A squared term for postneonatal mortality rate was included to allow for nonlinearity due to the increasing role of selective mortality at higher adversity levels. Postneonatal mortality rate was centered around percentile 25 since scarring effects were expected at the lower end of the distribution: therefore, $\beta_{1}$ shows the effect of an additional postneonatal death per 100 person-years on height-for-age z-score or school attendance at percentile 25 of postneonatal mortality rate. $\delta_{s}$ were sibling (or neighborhood) fixed-effects, which were parametrically identical to adding a dummy coded variable for siblings (or neighborhoods).

Testing whether the effects of adversity in infancy varied by socioeconomic status (here, measured by maternal education) was explored by interacting postneonatal mortality rate with an indicator for whether the mother of the child had any education *e*:

$$y_{isnb}=\alpha +\delta_{s}+\beta_{1}{pnm}_{nb}+\beta_{2}{pnm}_{nb}^{2}+\beta_{3}\left(e_{s}{\times pnm}_{nb}\right)+\beta_{4}\left(e_{s}{\times pnm}_{nb}^{2}\right)+\theta^{\prime }x_{i}+\varepsilon_{isnb}$$

$\beta_{1}$ shows the effect of postneonatal mortality rate for children of mothers without education and the interaction term $\beta_{3}$ shows the difference in effect for children born to educated mothers. A baseline term for whether the mother had education was added when estimating neighborhood fixed-effects models. The interaction with sex was done using an indicator for being a female.

For twins, only one twin was considered (the firstborn, if more than one had valid data) for analyses using sibling fixed-effects since the postneonatal mortality rate did not vary between twins. For height-for-age, 141,603 children did not have a sibling with valid data and were excluded from siblings fixed-effects models, and 2874 neighborhoods had only a single valid observation and were excluded from neighborhood fixed-effects models (Supplementary Table S2). For school attendance, 118,575 children without a sibling with valid data were excluded when comparing siblings and 1246 neighborhoods with a single valid observation when comparing children within neighborhoods (Supplementary Table S3). These excluded children were very similar to children in the full sample, apart from variables relating to maternal fertility for children remaining in the analyses using sibling fixed-effects, who had more siblings and shorter birth intervals (Table 1 and Supplementary Table S6).

First, results from regression models were tabulated. Second, the marginal effects of the postneonatal mortality rate were estimated by first taking the partial derivative of the regression equation with respect to postneonatal mortality rate, and then estimating across the distribution of postneonatal mortality rate. The resulting marginal effects were graphed between percentile 5 and 95 of the postneonatal mortality rate distribution and then tabulated at percentiles 1, 5, 10, 25, 50, 75, 90, 95, and 99. (Percentiles of postneonatal mortality rate referred to the distribution for children that were included in either the height-for-age or school attendance sample before excluding children without siblings with valid data or single observations within neighborhoods so that the marginal effects were estimated at the same percentiles regardless of the outcome or the level of fixed-effects used.)

The results from neighborhood and sibling fixed-effects models were expected to be similar since it is unclear how sibling-level factors influence postneonatal mortality rates. Results from models using survey-fixed effects were shown in the Supplement (Tables S6–S8 and Figs. S3–S4). The expectation was that survey-fixed effects models would show a greater negative association across most of the distribution of postneonatal mortality rates since these models utilized both the temporal and spatial variation, and areas with higher rates on average are likely to be disadvantaged in many other aspects, which may even stretch across generations. Estimates were not weighted but standard errors were adjusted for clustering within primary sampling units.

### 3.6 Sensitivity analyses

Ten sensitivity analyses were done: 1) Deaths and children at risk were further restricted to a 25 km radius when constructing postneonatal mortality rate (Supplementary Tables S9–S10 and Figs. S5–S6). 2) Postneonatal mortality rate was distance-weighted within a 50 km radius, with weights defined as the natural logarithm of 50 minus the distance between the target neighborhood and each of the neighborhoods (Supplementary Tables S11–S12 and Figs. S7–S8). 3) Postneonatal mortality rate was restricted to at least 1200 person-months of exposure to further reduce noise (Supplementary Tables S13–S14 and Figs. S9–S10).

4) Neonates were included in the adversity measures, which was, therefore, an infant mortality rate (Supplementary Tables S15–S16 and Figs. S11–S12): Since neonatal mortality is determined to a greater extent by factors other than nutrition and infections, and since neonatal deaths are a large share of overall infant deaths (Li et al., 2021), their inclusion may add noise to the measure of adversity, which would reduce the association with subsequent child health outcomes.

5) Cubed term for postneonatal mortality rate was added to the regression models to test the robustness of the results to a more irregular relationship (Supplementary Tables S17–S18 and Figs. S13–S14). 6) Births occurring more than ten years before a survey were excluded when constructing the postneonatal mortality rate which would reduce the potential influence of maternal age censoring (UN, 2011): since, for example, 15 years before a survey only births to women 34 years and younger were captured in the postneonatal mortality rate (Supplementary Tables S19–S20 and Figs. S15–S16). It may also reduce errors due to migration.

7) Neighborhood fixed-effects models were estimated on samples restricted to children that had valid sibling data to explore whether differences in estimates between neighborhood and sibling fixed-effects models were due to sample restrictions (Supplementary Tables S21–S22 and Figs. S17–S18). 8) Postneonatal mortality rate was linked to a 12 month period starting at conception (ie, 9 months before birth), since pregnant mother's may be exposed to the infections and undernutrition associated with mortality, which may be observable in growth and school attendance later in childhood (Supplementary Tables S23–S24 and Figs. S19–S20). 9) School attendance was modeled using logit models instead of linear models (Supplementary Tables S25–S26 and Fig. S21): Sibling-level means for all valid observations were added to the logit models to control for sibling-level factors while avoiding the incidental parameter problem from using many fixed-effects with logit models (Wooldridge, 2019). 10) Postneonatal mortality rate was converted to natural log scale (Supplementary Tables S27–S28 and Figs. S22–S23).

## 4 Results

### 4.1 Descriptive statistics

On average, children were 1.8 z-score (95% confidence interval [CI]: −1.81, −1.8) below the median in the reference population and 76% (95% CI: 0.757, 0.77) attended school (Table 1). Postneonatal mortality rate was on average 5.25 (95% CI: 5.2, 5.3) deaths per 100 person-years of exposure in the height-for-age sample and 6.35 (95% CI: 6.31, 6.4) in the school attendance sample. These numbers were similar in the samples restricted to siblings.

### 4.2 Main results

A single postneonatal death increase per 100 person-years was associated with a 0.015 (95% CI: −0.026, −0.0046) z-score lower height-for-age (at percentile 25 of postneonatal mortality rate) when estimating neighborhood fixed-effects models (Table 2). When using sibling fixed-effects models, this association was −0.026 (95% CI: −0.05, −0.002). The squared term coefficient was positive, indicating a diminishing impact of postneonatal mortality rate on height-for-age at higher rates of postneonatal mortality.

**Table 2:** Results from linear regression models.

Outcome->	Height-for-age z-score		School attendance	
Fixed-effects->	Neighborhood	Sibling	Neighborhood	Sibling
Independent variables				
Postneonatal mortality rate (per 100)	−.015***	−.026**	−.0016***	−.0007
	[-.026, −.0046]	[-.05, −.002]	[-.0028, −.00046]	[-.0025, .0011]
Postneonatal mortality rate (per 100) squared	.0011**	.0021**	.00034***	.00028***
	[.00021, .0019]	[.00033, .0039]	[.00023, .00044]	[.00011, .00044]
Firstborn	.2***	.24***	−.000099	−.015***
	[.17, .24]	[.14, .33]	[-.0041, .0039]	[-.022, −.0085]
Birth interval (months)	.0047***	.007***	.00006*	−.00024**
	[.0042, .0051]	[.005, .009]	[-2.0e-06, .00012]	[-.00038, −.000098]
Birth order	−.23***	−1.1***	.00075	−.015***
	[-.25, −.21]	[-1.2, −.98]	[-.00066, .0022]	[-.021, −.0095]
Age (months)	−.058***	−.087***	.014***	.014***
	[-.061, −.054]	[-.095, −.08]	[.013, .014]	[.014, .015]
Age (months) squared	.00072***	.00073***	−.000049***	−.000053***
	[.00068, .00077]	[.00063, .00083]	[-.00005, −.000048]	[-.000055, −.000052]
Female	.16***	.17***	−.022***	−.028***
	[.14, .17]	[.13, .21]	[-.024, −.02]	[-.031, −.024]
Mother's age at birth (years)	.047***		−.0017**	
	[.037, .057]		[-.0034, −.000035]	
Mother's age at birth (years) squared	−.0005***		.000022	
	[-.00067, −.00032]		[-9.8e-06, .000053]	
Mother's education (years)	.035***		.0091***	
	[.032, .038]		[.0087, .0095]	
Number of siblings	.2***		−.0048***	
	[.18, .22]		[-.0059, −.0038]	
Twin	−.62***	−.7***	.0095**	.011
	[-.68, −.56]	[-.88, −.52]	[.0018, .017]	[-.0032, .026]
Constant	−1.8***	−1.7***	.76***	.76***
	[-1.8, −1.8]	[-1.8, −1.7]	[.76, .76]	[.75, .76]

R squared	0.267	0.699	0.433	0.706
Observations	196,543	57,814	441,159	323,830

Notes: ***p < 0.01; **p < 0.05; *p < 0.1. Linear regression coefficients are shown. Postneonatal mortality rate was centered around percentile 25 (considering the pooled samples for both outcomes) and all covariates were mean-centered (using means for all valid observations in each analysis): therefore, the constant shows the mean outcome when covariates were at their means and postneonatal mortality rate was at percentile 25, and the coefficient of the linear term for postneonatal mortality rate shows the marginal effect at percentile 25. 95% confidence intervals (shown in brackets) and p-values were adjusted for clustering within primary sampling units. Postneonatal mortality rate per 100 person-years within a 50 km radius was linked to the period of infancy.

When using neighborhood fixed-effects, the association between postneonatal mortality rate and school attendance indicated 0.0016 (95% CI: −0.0028, −0.00046) lower probability of attending school for an additional death at percentile 25 of postneonatal mortality rate. When using sibling fixed-effects models, that association was small and not statistically significant: However, the coefficient for the squared term of postneonatal mortality rate was positive and statistically significant. Females had a 0.028 (95% CI: −0.031, −0.024) lower probability of attending school.

**Fig. 2:**
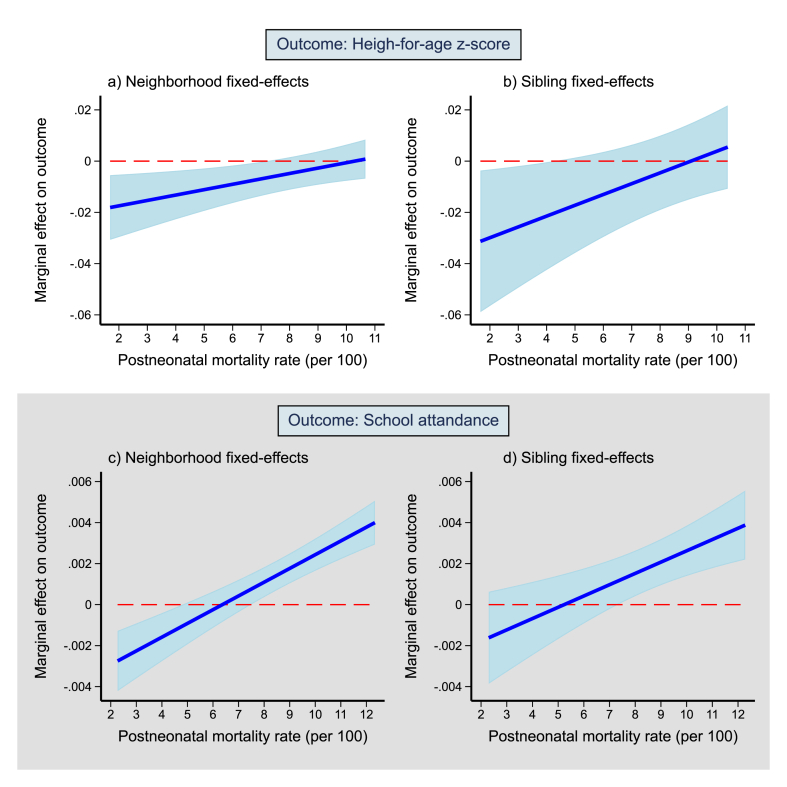
Marginal effects of postneonatal mortality rate on height-for-age and school attendance across the distribution of postneonatal mortality rate. Notes: The values on the y-axis indicate marginal effects: that is, change in the outcome for a single death increase in postneonatal mortality rate per 100 person-years. Since the relationship was nonlinear, the marginal effects vary across the distribution of postneonatal mortality rate. The marginal effects were obtained using the partial derivative of the regression equation with respect to postneonatal mortality rate. The plot was restricted to postneonatal mortality rate between the 5th and 95th percentile (considering the pooled samples for both outcomes). 95% confidence intervals adjusted for clustering within primary sampling units are shown. Postneonatal mortality rate per 100 person-years within a 50 km radius was linked to the period of infancy.

The marginal effect of postneonatal mortality rate on height-for-age was negative at the bottom of the postneonatal mortality rate distribution, while it attenuated at higher rates of postneonatal mortality, eventually reaching zero at the top of the distribution (Fig. 2). For school attendance, the marginal effect of postneonatal mortality rate was negative at very low levels of postneonatal mortality rate, although it was small and not statistically significant when using sibling fixed-effects. In contrast, the marginal effect was positive at higher levels of postneonatal mortality rate.

The interquartile range of postneonatal mortality rate was 4.2 deaths (Table 3). At the bottom of the distribution, at the first percentile (ie, 1.4 deaths per 100), an additional postneonatal death per 100 was associated with a 0.017 (95% CI: −0.029, −0.0052) z-score decrease in height-for-age when using neighborhood fixed-effects models and 0.03 (95% CI: −0.056, −0.0032) decrease when using sibling fixed-effects. At percentile 75 (ie, 7.8 deaths per 100), an additional postneonatal death per 100 was associated with a 0.0073 (95% CI: −0.014, −0.00056) z-score decrease in height-for-age when using neighborhood fixed effects and a non-statistically significant 0.01 (95% CI: −0.026, 0.0053) z-score decrease when using sibling fixed-effects. At the top of the postneonatal mortality rate distribution—at the percentile 99 (ie, 15.8 deaths per 100)—there was a positive but non-statistically significant association between postneonatal mortality rate and height-for-age, both when using neighborhood (0.0089, 95% CI: −0.0036, 0.021) and sibling (0.022, 95% CI: −0.0039, 0.047) fixed-effects.

**Table 3:** Marginal effects of postneonatal mortality rate on height-for-age and school attendance at different percentiles of postneonatal mortality rate.

Outcome->	Height-for-age z-score		School attendance	
Fixed-effects->	Neighborhood	Sibling	Neighborhood	Sibling
Percentiles of postneonatal mortality rate (per 100)				
p1 (1.4)	−.019***	−.033**	−.0032***	−.002
	[-.033, −.0058]	[-.062, −.0042]	[-.0048, −.0016]	[-.0045, .00043]
p5 (2.0)	−.018***	−.031**	−.0027***	−.0016
	[-.031, −.0055]	[-.059, −.0037]	[-.0042, −.0013]	[-.0039, .00063]
p10 (2.5)	−.017***	−.03**	−.0024***	−.0013
	[-.029, −.0052]	[-.056, −.0032]	[-.0038, −.001]	[-.0034, .00076]
p25 (3.6)	−.015***	−.026**	−.0016***	−.0007
	[-.026, −.0046]	[-.05, −.002]	[-.0028, −.00046]	[-.0025, .0011]
p50 (5.4)	−.012***	−.019*	−.00038	.00033
	[-.02, −.0032]	[-.039, .00042]	[-.0013, .00053]	[-.001, .0017]
p75 (7.8)	−.0073**	−.01	.0012***	.0016***
	[-.014, −.00056]	[-.026, .0053]	[.00047, .0019]	[.0005, .0027]
p90 (10.2)	−.0026	−.0013	.0028***	.0029***
	[-.0091, .0039]	[-.016, .013]	[.002, .0037]	[.0016, .0043]
p95 (11.9)	.00078	.0055	.004***	.0039***
	[-.0068, .0084]	[-.011, .022]	[.0029, .0051]	[.0022, .0056]
p99 (15.8)	.0089	.022*	.0066***	.006***
	[-.0036, .021]	[-.0039, .047]	[.0049, .0084]	[.0033, .0088]

Notes: ***p < 0.01; **p < 0.05; *p < 0.1. Change in outcome for a single increase in postneonatal mortality rate per 100 person-years are shown at different percentiles of postneonatal mortality rate. Since the relationship was nonlinear, the marginal effects vary across the distribution of postneonatal mortality rate. The marginal effects were obtained using the partial derivative of the regression equation with respect to postneonatal mortality rate. The level of postneonatal mortality rate per 100 at each of the percentile is shown in parentheses (considering the pooled samples for both outcomes). 95% confidence intervals adjusted for clustering within primary sampling units are shown in brackets. Postneonatal mortality rate per 100 person-years within a 50 km radius was linked to the period of infancy.

At the bottom of the postneonatal mortality rate distribution, at percentile 10, an additional postneonatal death per 100 was associated with a 0.0024 (95% CI: −0.0038, −0.001) decrease in the probability of attending school when using neighborhood fixed-effects models and a non-statistically significant 0.0013 (95% CI: −0.0034, 0.00076) decrease when applying sibling fixed-effects models. At the top of the postneonatal mortality rate distribution, for example at percentile 90, an increase of a single postneonatal death per 100 was associated with a 0.0028 (95% CI: 0.002, 0.0037) increase in the probability of attending school when using neighborhood fixed-effects and a 0.0029 (95% CI: 0.0016, 0.0043) increase when using sibling fixed-effects.

Survey fixed-effects models indicated a negative effect of postneonatal mortality rate on both height-for-age and school attendance across the whole distribution of postneonatal mortality rate (Supplementary Tables S7–S8 and Figs. S3–S4).

### 4.3 Results by socioeconomic status and sex

**Fig. 3:**
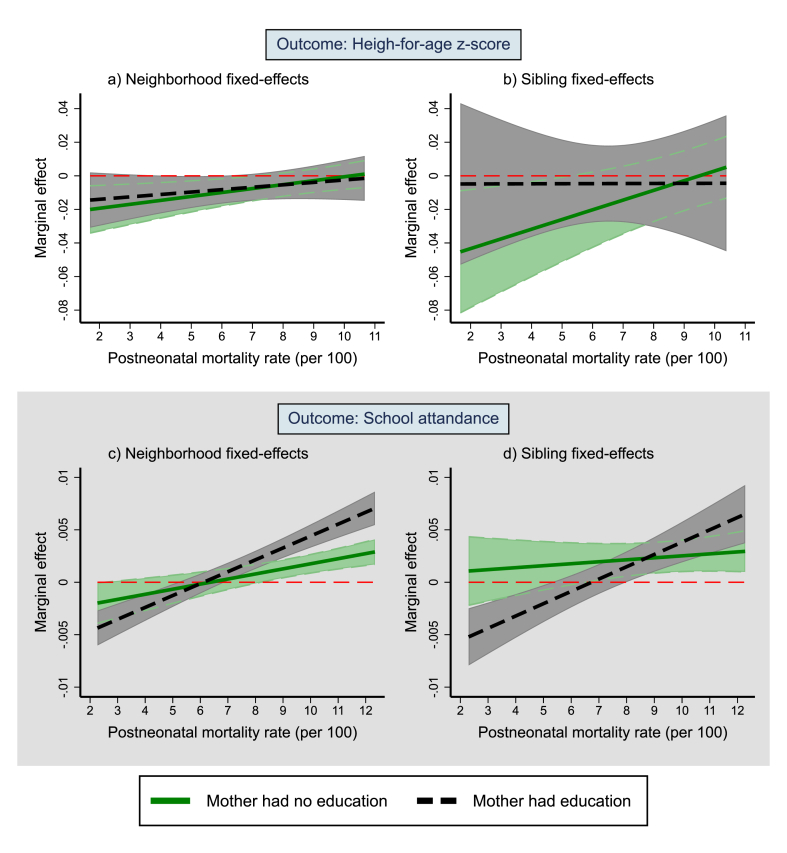
Marginal effects of postneonatal mortality rate on height-for-age and school attendance across the distribution of postneonatal mortality rate: by maternal education. Notes: The values on the y-axis indicate marginal effects: that is, change in the outcome for a single death increase in postneonatal mortality rate per 100 person-years. Since the relationship was nonlinear, the marginal effects vary across the distribution of postneonatal mortality rate. The marginal effects were obtained using the partial derivative of the regression equation with respect to postneonatal mortality rate. The plot was restricted to postneonatal mortality rate between the 5th and 95th percentile (considering the pooled samples for both outcomes). 95% confidence intervals adjusted for clustering within primary sampling units are shown. Postneonatal mortality rate per 100 person-years within a 50 km radius was linked to the period of infancy. See Supplementary Tables S29–S30 and Figs. S24–S25 complete results by maternal education.

When using neighborhood fixed-effects models, the marginal effects of postneonatal mortality rate on height-for-age for children of mothers with education overlapped with that of children of mothers without education across the postneonatal mortality rate distribution (Fig. 3). When using sibling fixed-effects models, children of mothers without education had a stronger marginal effect of postneonatal mortality rate on height-for-age, particularly at the low end of the postneonatal mortality rate distribution, while children of mothers with education had a marginal effect close to zero across the distribution. For school attendance, children of educated mothers had a stronger positive association at the top of the distribution of postneonatal mortality rate and a stronger negative association at the bottom of the distribution.

**Fig. 4:**
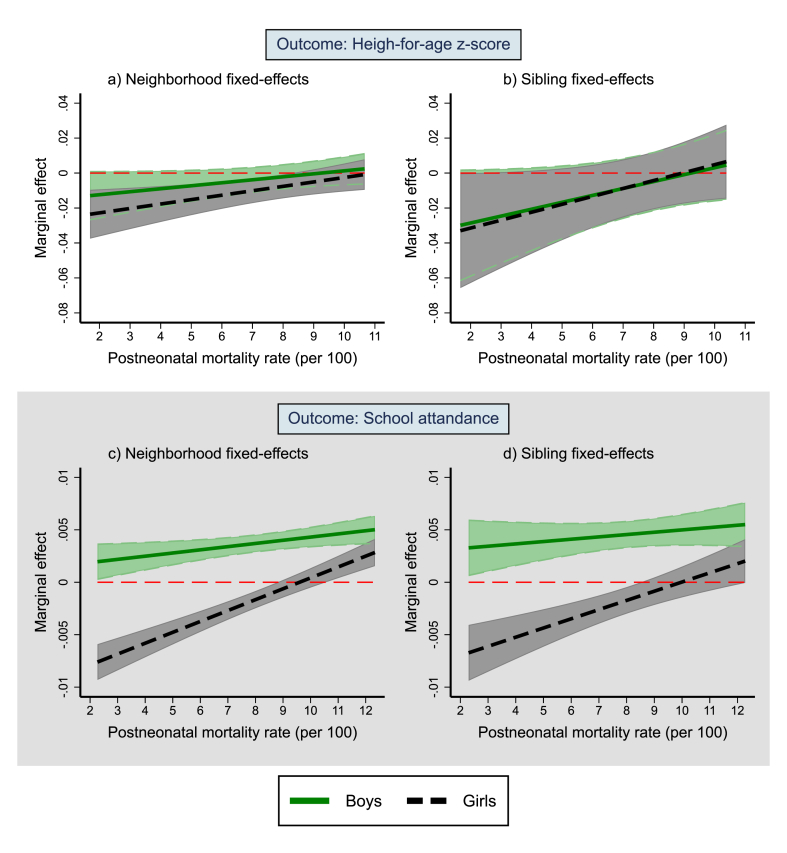
Marginal effects of postneonatal mortality rate on height-for-age and school attendance across the distribution of postneonatal mortality rate: by sex. Notes: The values on the y-axis indicate marginal effects: that is, change in the outcome for a single death increase in postneonatal mortality rate per 100 person-years. Since the relationship was nonlinear, the marginal effects vary across the distribution of postneonatal mortality rate. The marginal effects were obtained using the partial derivative of the regression equation with respect to postneonatal mortality rate. The plot was restricted to postneonatal mortality rate between the 5th and 95th percentile (considering the pooled samples for both outcomes). 95% confidence intervals adjusted for clustering within primary sampling units are shown. Postneonatal mortality rate per 100 person-years within a 50 km radius was linked to the period of infancy. See Supplementary Tables S31–S32 and Figs. S26–S27 complete results by sex.

When using neighborhood fixed-effects, the marginal effect of postneonatal mortality rate on height-for-age was considerably stronger for females, particularly the negative association observed at the bottom of the postneonatal mortality rate distribution (Fig. 4). The interaction terms between postneonatal mortality rate and sex indicated a statistically significant stronger negative association for females at percentile 25 (−0.0095, 95% CI: −0.019, −0.00016; Supplementary Table S31). When using sibling fixed-effects, the marginal effects were very similar between males and females.

For boys, the marginal effects of postneonatal mortality rate on school attendance were positive across the whole distribution of postneonatal mortality rate and increased somewhat as the postneonatal mortality rate increased (although the squared term was only statistically significant when using neighborhood fixed-effects). For females, the marginal effect of postneonatal mortality rate on school attendance was negative at the bottom of the distribution of postneonatal mortality rate, decreased at higher rates of postneonatal mortality, and was positive at high levels of postneonatal mortality rate. Females had a 0.011 (95% CI: −0.016, −0.007) lower probability of attending school (when postneonatal mortality rate was at percentile 25) when estimated using sibling fixed-effects (Supplementary Table S31).

### 4.4 Results from sensitivity analyses

Sensitivity analyses were broadly consistent with the main results: some statistically significant coefficients in the main analyses were not in the sensitivity analyses, although remaining similar in magnitude.

As expected, replacing the postneonatal mortality rate with infant mortality rate indicated much smaller effect sizes, particularly for height-for-age, likely due to less weight on undernutrition and infections in that measure (Supplementary Tables S15–S16 and Figs. S11–S12).

Adding cubed postneonatal mortality rate to the regression equation indicated that a regression with just a linear and squared term approximated the relationships similarly (Supplementary Tables S17–S18 and Figs. S13–S14): However, the increasing positive marginal effects on school attendance diminished somewhat at very high rates of postneonatal mortality.

Indexing postneonatal mortality rate to the 12-month period starting at conception yielded mostly null results (Supplementary Tables S23–S24 and Figs. S19–S20).

Results for school attendance were more similar between neighborhood and sibling fixed-effects models after converting postneonatal mortality rate to natural log scale (Supplementary Tables S27–S28 and Figs. S22–S23).

## 5 Discussion

### 5.1 Summary and interpretation

The findings suggest a negative effect of exposures to adversity in infancy on height-for-age at age 1–4 years, indicating net-scarring effects on human development for those who survive. These effects were strong at the bottom of the distribution of postneonatal mortality rate: For example, at percentile 10 (ie, 2.5 deaths per 100), a 0.03 decline in height-for-age was observed for a further increase of a single postneonatal death per 100 person-years between siblings in infancy—or 1.7% of the sample mean deficit in height-for-age relative to the 2006 World Health Organization growth standard. This effect size is rather small but meaningful considering that the interquartile range of postneonatal mortality rate was 4.2 deaths. To put this effect size into perspective: complementary feeding interventions have resulted in 0.11–0.25 z-score increase in length-for-age (Panjwani & Heidkamp, 2017; Pickering et al., 2019) and eliminating all diarrhea before age two has been suggested to improve length-for-age by 0.13 z-score (Pickering et al., 2019; Richard et al., 2013). (The effect was somewhat smaller when estimated using neighborhood fixed-effects models.) At a higher rate of postneonatal mortality, a further increase in postneonatal mortality rate had a weaker marginal effect on height-for-age, reaching zero above approximately percentile 90 (ie, 10.2 deaths per 100).

For school attendance, the findings only indicated a negative net-scarring effect at the bottom of the distribution of postneonatal mortality rate and only when comparing children within the same neighborhood and not when comparing siblings. The marginal effects of postneonatal mortality rate on school attendance were positive but small above the median postneonatal mortality rate, and similar between neighborhood and sibling fixed-effects models at high rates: For example, at percentile 90, a further increase of a single postneonatal death per 100 person-years between siblings in infancy was associated with a 0.0029 increase in the probability of attending school at age 7–16 years—or about 0.4% of the sample mean school attendance.

The absence of a marginal effect between postneonatal mortality rate and height-for-age at rates of postneonatal mortality that were already relatively high was consistent with the selection mechanism increasing at high levels of adversity (Bozzoli et al., 2009; Deaton, 2007; Gørgens et al., 2012). In this study, selective mortality appears to completely cancel out observable scarring effects on height-for-age at around percentile 90 of postneonatal mortality rate. A study of adult heights in cohorts born earlier than those included in this study, based in an environment with considerably greater adversity, found a positive association between adversity in early-life—measured as the under-five mortality rate in the country and year of birth—indicating that selection dominated over scarring (Deaton, 2007): The relative importance of selection may have decreased for cohorts in this study, who were born more recently and into more favorable environments. However, since this paper studied outcomes observed in childhood rather than adulthood, there may also have been some further selection or variation in catch-up growth before these children reached adulthood (Preston et al., 1998; Steckel & Ziebarth, 2016). There have also been indications that adult height may not adequately reflect childhood morbidity in many sub-Saharan African countries, since adults are relatively tall while child morbidity is high (Akachi & Canning, 2010; Karlsson et al., 2021).

Children of mothers without education had a stronger association between postneonatal mortality rate and height-for-age, and the marginal effect was close to zero across the distribution for children of mothers with education: However, this heterogeneity was only observed when comparing siblings, where all family level factors were taken into account.

The marginal effect on height-for-age was somewhat stronger for girls than boys, particularly at low rates of postneonatal mortality, which may either indicate that girls were more vulnerable to exposures to adversity or that selective mortality cancels out more of the net-scarring effects on boys. The latter interpretation is more consistent with the observation that boys tend to be more sensitive to adverse exposures due to biological frailty (Sawyer, 2012; Wamani et al., 2007). However, the stronger effects on height-for-age for girls were modest and only observed when comparing children within neighborhoods, while the effects for boys and girls were highly similar when comparing siblings.

There was major heterogeneity in the effects of postneonatal mortality rate on school attendance by sex. The effect was positive for boys across the postneonatal mortality rate distribution. For girls, it was negative at the lower end of the distribution and positive at the top of the distribution. (In addition, girls had about 0.03 lower probability of attending school altogether.) Applying the interpretation that selection effects were greater among boys, the positive link between school attendance and postneonatal mortality rate in infancy would suggest that selection effects dominate over any scarring effects, even at relatively low levels of adversity. The increase in the positive marginal effect on school attendance at higher rates of postneonatal mortality was modest for boys, which would suggest that the increase in selection effects at greater levels of adversity was also modest. For girls, the results were consistent with scarring effects dominating over selection effects at low levels of adversity, while selection effects were more important at high levels of adversity.

However, acquired immunity, reduced cohort sizes due to mortality, and increased investments in surviving children or other factors cannot be ruled out as mechanisms causing higher school attendance among those exposed to a higher postneonatal mortality rate in infancy. For example, sons might have received greater investment following times of elevated adversity. In addition, effect sizes were small. Factors such as acquired immunity and greater investments in surviving children following adversity may also be an alternative to selective mortality biasing the effects on height-for-age downward.

Results were mostly consistent between neighborhood and sibling fixed-effects models. Still, differences may be related to omitted family level variables biasing the estimates from the neighborhood-fixed effects models (sensitivity analysis indicated that differences were unrelated to sample restrictions). The differences in results obtained from survey fixed-effects models using spatial and temporal variation were consistent with correlated environments and a more general level of harmful exposures in areas where the average rate of postneonatal mortality was greater, exaggerating the negative effects of exposures to adversity when not accounting for the spatial variation in some form (Preston et al., 1998).

### 5.2 Limitations

A limitation of this paper was the use of a general measure of adversity that captures a host of infectious diseases in interaction with undernutrition. Other harmful exposures such as air pollution or armed conflict cannot be ruled out as contributing to adversity, although, these factors also affect child mortality through undernutrition and infections (Kadir et al., 2019; World Health Organization, 2018). Further, adversity occurring while in utero could correlate with and contribute to postneonatal deaths (although, indexing postneonatal mortality rate to the 12 months following conceptions yielded mostly null results). However, this paper established a nonlinear relationship between adversity reflected in mortality and outcomes for surviving children.

Calculating a relatively high-resolution estimate, such as the postneonatal mortality rate in this paper, requires enough deaths and exposure time to acquire a reliable estimate. Therefore, these estimates were not representative, as areas that were more extensively surveyed, more densely populated, and had higher mortality were more likely to be included in the analyses. These areas were less likely to be rural but also tended to be more disadvantaged overall, when measured as maternal education (but more advantaged when comparisons were made within each survey).

Although the exposure used in this paper was exogenous with respect to child health, its effects were strictly not causal due to misclassification and noise in the adversity measure: First, not all children faced adversity when the postneonatal mortality rate was high, and some children faced adversity even when the postneonatal mortality rate was low, which has similar implications as misclassification (Almond & Mazumder, 2011; Van den Berg et al., 2016). Also, heterogeneity across socioeconomic status may have reflected variation in misclassification since the actual exposure to adversity may have differed. Second, due to a lack of information on migration, the neighborhood where the mother resided at the time of the survey was assumed to be the same as where her children were born when constructing the postneonatal mortality rate, which could cause misclassification if mothers migrated after giving birth. However, for 63% of births in the birth histories, the mother answered a question on “Years lived in place of residence,” and thereof, 45% of births were to children whose mother had never migrated and 81% were to children whose mother had not migrated after giving birth to their first child. Third, the standard errors in the estimation of postneonatal mortality rate added noise to the adversity measures.

Further, fixed-effects models can enhance the problem of misclassification, or measurement errors, most likely biasing estimated effects sizes toward zero (Angrist & Pischke, 2008). Therefore, the effect sizes in this paper may be underestimated. However, sensitivity analyses which restricted estimation of the postneonatal mortality rate to areas with more data (which would reduce standard errors), to a 25-km radius (which would reduce misclassification in actual exposure to adversity), and births ten years before the survey (which reduced noise from migration as well as maternal age censoring) yielded estimates of similar magnitude as the main results.

Finally, selection effects may have biased the observed net-scarring effects downward, even at relatively low rates of postneonatal mortality, which was still high in the context studied here (eg, 2.5 per 100 at percentile 10).

## 6 Conclusions

In sub-Saharan Africa, being exposed to elevated postneonatal mortality rate during infancy negatively affected the observed height-for-age of surviving children at age 1–4 years. However, for increases at rates of postneonatal mortality that were already very high, selective mortality may have canceled out net-scarring effects. School attendance at ages 7–16 was slightly greater for children exposed to increased adversity during infancy when postneonatal mortality rate was already high. Selective mortality may obscure some of the detrimental consequences of adversity in early life when studying the outcomes of the survivors. However, although effect sizes were rather small, particularly for school attendance, they were likely to be underestimated.

## Compliance with ethical standards

This project used publicly accessible secondary data obtained from the Demographic and Health Surveys (DHS) website. The DHS data were not collected specifically for this study and the author had no access to identifiers linked to the data. These activities did not meet the regulatory definition of human subject research. Therefore, an Institutional Review Board (IRB) review was not required. The Harvard Logwood Campus IRB allows researchers to self-determine when their research does not meet the requirements for IRB oversight via guidance online. The ICF IRB and local IRB approved data collection procedures and questionnaires and the U.S. Center for Disease Control and Prevention (CDC) reviewed protocols.

## Contributions

Karlsson was the sole author of this study.

## Funding

None.

## Data availability

The Demographic and Health Surveys are available at https://dhsprogram.com (requiring a simple application).

## Declaration of competing interest

None.
