# Supplementary material for: Scarring and selection effects on children surviving elevated rates of postneonatal mortality in sub-Saharan Africa
Source: SSM Popul Health. 2022 Jul 2;19:101160. doi: 10.1016/j.ssmph.2022.101160 (PMC9283665; doi:10.1016/j.ssmph.2022.101160)
Supplement: Multimedia component 1 [file mmc1.pdf]

SUPPLEMENT

Scarring and selection effects on children surviving elevated rates of postneonatal mortality in sub-Saharan Africa

SUPPLEMENT 1

SUPPLEMENT 1: Causes of postneonatal deaths in sub-Saharan Africa 4

Table S1. Percentage (and rank) of deaths from the top 20 causes among infants after the neonatal period in sub-Saharan Africa: Total and by sub-region 5

SUPPLEMENT 2: Information on data 7

Table S2. Sample size and missing data for the sample used for analyses of height-for-age: pooled and by survey 8

Table S3. Sample size and missing data for the sample used for analyses of school attendance: pooled and by survey 11

Table S4. Linear regressions comparing those with and without missing data for height-for-age and postneonatal mortality rate linked to the height-for-age sample 13

Table S5. Linear regressions comparing those with and without missing data for school attendance and postneonatal mortality rate linked to the school attendance sample 14

Figure S1. Map of the study area: Neighborhoods in the analyses of school attendance 15

Figure S2. Histogram for height-for-age and postneonatal mortality rate 16

SUPPLEMENT 3: Results including survey fixed-effects models 17

Table S6. Descriptive statistics including for full sample used with survey fixed-effects models 18

Table S7. Results from linear regression models: Results including survey fixed-effects models 19

Figure S3. Marginal effects of postneonatal mortality rate on height-for-age across the distribution of postneonatal mortality rate: Results including survey fixed-effects models 20

Figure S4. Marginal effects of postneonatal mortality rate on school attendance across the distribution of postneonatal mortality rate: Results including survey fixed-effects models 21

Table S8. Marginal effects of postneonatal mortality rate on height-for-age and school attendance at different percentiles of postneonatal mortality rate: Results including survey fixed-effects models 22

SUPPLEMENT 4: Sensitivity analyses 23

Table S9. Results from linear regression models: Postneonatal mortality rate with distance restricted to 25 kilometers 24

Figure S5. Marginal effects of postneonatal mortality rate on height-for-age across the distribution of postneonatal mortality rate: Postneonatal mortality rate with distance restricted to 25 kilometers 25

Figure S6. Marginal effects of postneonatal mortality rate on school attendance across the distribution of postneonatal mortality rate: Postneonatal mortality rate with distance restricted to 25 kilometers 26

Table S10. Marginal effects of postneonatal mortality rate on height-for-age and school attendance at different percentiles of postneonatal mortality rate: Postneonatal mortality rate with distance restricted to 25 kilometers 27

Table S11. Results from linear regression models: Postneonatal mortality rate distance weighted 28

Figure S7. Marginal effects of postneonatal mortality rate on height-for-age across the distribution of postneonatal mortality rate: Postneonatal mortality rate distance weighted 29

Figure S8. Marginal effects of postneonatal mortality rate on school attendance across the distribution of postneonatal mortality rate: Postneonatal mortality rate distance weighted 30

Table S12. Marginal effects of postneonatal mortality rate on height-for-age and school attendance at different percentiles of postneonatal mortality rate: Postneonatal mortality rate distance weighted 31

Table S13. Results from linear regression models: Postneonatal mortality rate restricted to at least 4 deaths and 1200 person-months 32

Figure S9. Marginal effects of postneonatal mortality rate on height-for-age across the distribution of postneonatal mortality rate: Postneonatal mortality rate restricted to at least 4 deaths and 1200 person-months 33

Figure S10. Marginal effects of postneonatal mortality rate on school attendance across the distribution of postneonatal mortality rate: Postneonatal mortality rate restricted to at least 4 deaths and 1200 person-months 34

Table S14. Marginal effects of postneonatal mortality rate on height-for-age and school attendance at different percentiles of postneonatal mortality rate: Postneonatal mortality rate restricted to at least 4 deaths and 1200 person-months 35

Table S15. Results from linear regression models: Infant mortality rate (ie, including neonatal deaths) as a measure of adversity 36

Figure S11. Marginal effects of postneonatal mortality rate on height-for-age across the distribution of postneonatal mortality rate: Infant mortality rate (ie, including neonatal deaths) as a measure of adversity 37

Figure S12. Marginal effects of postneonatal mortality rate on school attendance across the distribution of postneonatal mortality rate: Infant mortality rate (ie, including neonatal deaths) as a measure of adversity 38

Table S16. Marginal effects of postneonatal mortality rate on height-for-age and school attendance at different percentiles of postneonatal mortality rate: Infant mortality rate (ie, including neonatal deaths) as a measure of adversity 39

Table S17. Results from linear regression models: Postneonatal mortality rate cubed added to regression models 40

Figure S13. Marginal effects of postneonatal mortality rate on height-for-age across the distribution of postneonatal mortality rate: Postneonatal mortality rate cubed added to regression models 41

Figure S14. Marginal effects of postneonatal mortality rate on school attendance across the distribution of postneonatal mortality rate: Postneonatal mortality rate cubed added to regression models 42

Table S18. Marginal effects of postneonatal mortality rate on height-for-age and school attendance at different percentiles of postneonatal mortality rate: Postneonatal mortality rate cubed added to regression models 43

Table S19. Results from linear regression models: Postneonatal mortality rate restricted to children born 10 years before survey 44

Figure S15. Marginal effects of postneonatal mortality rate on height-for-age across the distribution of postneonatal mortality rate: Postneonatal mortality rate restricted to children born 10 years before survey 45

Figure S16. Marginal effects of postneonatal mortality rate on school attendance across the distribution of postneonatal mortality rate: Postneonatal mortality rate restricted to children born 10 years before survey 46

Table S20. Marginal effects of postneonatal mortality rate on height-for-age and school attendance at different percentiles of postneonatal mortality rate: Postneonatal mortality rate restricted to children born 10 years before survey 47

Table S21. Results from linear regression models: Neighborhood fixed-effects on the sibling sample 48

Figure S17. Marginal effects of postneonatal mortality rate on height-for-age across the distribution of postneonatal mortality rate: Neighborhood fixed-effects on the sibling sample 49

Figure S18. Marginal effects of postneonatal mortality rate on school attendance across the distribution of postneonatal mortality rate: Neighborhood fixed-effects on the sibling sample 50

Table S22. Marginal effects of postneonatal mortality rate on height-for-age and school attendance at different percentiles of postneonatal mortality rate: Neighborhood fixed-effects on the sibling sample 51

Table S23. Results from linear regression models: Postneonatal mortality rate indexed to a 12 month period starting at conception 52

Figure S19. Marginal effects of postneonatal mortality rate on height-for-age across the distribution of postneonatal mortality rate: Postneonatal mortality rate indexed to a 12 month period starting at conception 53

Figure S20. Marginal effects of postneonatal mortality rate on school attendance across the distribution of postneonatal mortality rate: Postneonatal mortality rate indexed to a 12 month period starting at conception 54

Table S24. Marginal effects of postneonatal mortality rate on height-for-age and school attendance at different percentiles of postneonatal mortality rate: Postneonatal mortality rate indexed to a 12 month period starting at conception 55

Table S25. Results from linear regression models: logit models instead of linear regressions 56

Figure S21. Marginal effects of postneonatal mortality rate on school attendance across the distribution of postneonatal mortality rate: logit models instead of linear regressions 57

Table S26. Marginal effects of postneonatal mortality rate on school attendance at different percentiles of postneonatal mortality rate: logit models instead of linear regressions 58

Table S27. Results from linear regression models: Postneonatal mortality rate converted to natural log scale 59

Figure S22. Marginal effects of postneonatal mortality rate on height-for-age across the distribution of postneonatal mortality rate: Postneonatal mortality rate converted to natural log scale 60

Figure S23. Marginal effects of postneonatal mortality rate on school attendance across the distribution of postneonatal mortality rate: Postneonatal mortality rate converted to natural log scale 61

Table S28. Marginal effects of postneonatal mortality rate on height-for-age and school attendance at different percentiles of postneonatal mortality rate: Postneonatal mortality rate converted to natural log scale 62

SUPPLEMENT 5: Full results by sex and mother's education attainment 63

Table S29. Results from linear regression models: Postneonatal mortality rate interacted with having a mother with education 64

Figure S24. Marginal effects of postneonatal mortality rate on height-for-age across the distribution of postneonatal mortality rate: by maternal education ... 66

Figure S25. Marginal effects of postneonatal mortality rate on school attendance across the distribution of postneonatal mortality rate: by maternal education 67

Table S30. Marginal effects of postneonatal mortality rate on height-for-age and school attendance at different percentiles of postneonatal mortality rate: by maternal education 68

Table S31. Results from linear regression models: Postneonatal mortality rate interacted with being female 70

Figure S26. Marginal effects of postneonatal mortality rate on height-for-age across the distribution of postneonatal mortality rate: by sex 72

Figure S27. Marginal effects of postneonatal mortality rate on school attendance across the distribution of postneonatal mortality rate: by sex 73

Table S32. Marginal effects of postneonatal mortality rate on height-for-age and school attendance at different percentiles of postneonatal mortality rate: by sex 74

SUPPLEMENT 1: Causes of postneonatal deaths in sub-Saharan Africa

Table S1. Percentage (and rank) of deaths from the top 20 causes among infants after the neonatal period in sub-Saharan Africa: Total and by sub-region

Region->	Total	Central	Eastern	Southern	Western
Year: 2019					
Diarrheal diseases	22.0 (1)	22.0 (2)	16.8 (2)	19.0 (2)	25.1 (1)
Lower respiratory infections	21.1 (2)	13.4 (3)	19.5 (1)	27.1 (1)	23.2 (2)
Malaria	15.0 (3)	27.1 (1)	14.2 (3)	1.7 (13)	14.0 (3)
Meningitis	4.5 (4)	2.1 (8)	3.3 (7)	1.9 (12)	5.7 (4)
Protein-energy malnutrition	3.8 (5)	4.0 (5)	5.7 (4)	7.0 (3)	2.5 (6)
Whooping cough	3.8 (6)	5.2 (4)	4.1 (5)	3.7 (5)	3.3 (5)
Congenital heart anomalies	2.7 (7)	1.9 (9)	3.6 (6)	2.1 (10)	2.3 (8)
Other congenital birth defects	2.0 (8)	1.5 (12)	2.4 (9)	1.4 (15)	1.9 (10)
Drug-susceptible tuberculosis	2.0 (9)	3.7 (6)	2.1 (12)	3.7 (6)	1.6 (12)
Neural tube defects	2.0 (10)	1.6 (11)	1.7 (13)	0.6 (22)	2.3 (7)
Measles	1.9 (11)	1.8 (10)	2.3 (10)	1.1 (17)	1.8 (11)
Syphilis	1.8 (12)	2.1 (7)	2.9 (8)	2.9 (8)	1.0 (15)
Sudden infant death syndrome	1.4 (13)	1.0 (13)	1.5 (14)	1.7 (14)	1.5 (13)
Invasive Non-typhoidal Salmonella (iNTS)	1.4 (14)	0.9 (16)	0.4 (26)	0.4 (29)	2.1 (9)
HIV/AIDS resulting in other diseases	1.3 (15)	0.9 (15)	2.1 (11)	5.3 (4)	0.6 (17)
Digestive congenital anomalies	1.1 (16)	0.9 (14)	1.2 (18)	0.7 (20)	1.1 (14)
Other neonatal disorders	0.9 (17)	0.6 (18)	1.4 (16)	2.0 (11)	0.5 (19)
Neonatal sepsis and other neonatal infections	0.8 (18)	0.5 (20)	1.5 (15)	1.3 (16)	0.4 (24)
Paralytic ileus and intestinal obstruction	0.7 (19)	0.5 (19)	0.7 (22)	0.3 (34)	0.8 (16)
Neonatal preterm birth	0.7 (20)	0.7 (17)	0.8 (20)	2.9 (7)	0.4 (20)
Year: 2010					
Diarrheal diseases	23.7 (1)	24.1 (1)	17.2 (2)	22.0 (2)	28.4 (1)
Lower respiratory infections	20.3 (2)	15.6 (3)	20.6 (1)	23.8 (1)	21.2 (2)
Malaria	15.7 (3)	23.1 (2)	12.9 (3)	2.0 (9)	16.5 (3)
Meningitis	4.8 (4)	2.9 (7)	4.2 (5)	2.1 (8)	6.0 (4)
Protein-energy malnutrition	4.8 (5)	5.4 (4)	7.8 (4)	5.6 (5)	2.3 (6)
Whooping cough	3.0 (6)	3.6 (6)	3.6 (6)	2.5 (7)	2.5 (5)
HIV/AIDS resulting in other diseases	2.6 (7)	1.5 (10)	3.5 (7)	11.3 (3)	1.5 (12)
Measles	2.5 (8)	2.0 (8)	3.4 (8)	1.8 (11)	2.1 (7)
Drug-susceptible tuberculosis	2.4 (9)	4.8 (5)	2.4 (10)	4.0 (6)	1.6 (11)
Congenital heart anomalies	2.0 (10)	1.3 (11)	2.7 (9)	1.3 (15)	1.7 (10)
Neural tube defects	1.6 (11)	1.5 (9)	1.6 (14)	0.3 (25)	1.8 (9)
Other congenital birth defects	1.6 (12)	1.2 (14)	1.9 (11)	0.9 (16)	1.5 (13)
Invasive Non-typhoidal Salmonella (iNTS)	1.3 (13)	1.3 (12)	0.3 (27)	0.2 (35)	2.0 (8)
Sudden infant death syndrome	1.2 (14)	0.9 (15)	1.3 (15)	1.3 (14)	1.2 (14)
HIV/AIDS - Drug-susceptible Tuberculosis	1.2 (15)	0.8 (16)	1.8 (12)	6.6 (4)	0.4 (18)
Syphilis	1.1 (16)	1.3 (13)	1.7 (13)	1.5 (13)	0.6 (16)
Digestive congenital anomalies	0.9 (17)	0.7 (17)	1.0 (18)	0.5 (21)	0.9 (15)
Other neonatal disorders	0.7 (18)	0.5 (19)	1.1 (17)	1.6 (12)	0.4 (20)
Neonatal preterm birth	0.6 (19)	0.7 (18)	0.8 (20)	2.0 (10)	0.3 (22)
Neonatal sepsis and other neonatal infections	0.6 (20)	0.3 (26)	1.1 (16)	0.8 (17)	0.3 (26)
Year: 2000					
Diarrheal diseases	24.7 (1)	26.0 (1)	19.9 (1)	21.8 (1)	29.0 (1)
Lower respiratory infections	20.1 (2)	16.9 (3)	19.6 (2)	18.5 (3)	21.7 (2)
Malaria	14.3 (3)	20.0 (2)	14.3 (3)	1.8 (9)	13.7 (3)
Measles	5.8 (4)	3.4 (7)	6.4 (5)	0.5 (19)	6.5 (4)
Protein-energy malnutrition	5.1 (5)	6.4 (4)	7.2 (4)	3.2 (5)	3.0 (6)
Meningitis	4.2 (6)	3.0 (8)	3.5 (7)	1.5 (10)	5.4 (5)
HIV/AIDS resulting in other diseases	3.7 (7)	1.3 (9)	5.1 (6)	20.8 (2)	1.7 (9)
Whooping cough	2.8 (8)	3.4 (6)	2.9 (8)	1.9 (8)	2.6 (7)
Drug-susceptible tuberculosis	2.6 (9)	5.1 (5)	2.4 (10)	3.1 (6)	2.0 (8)
HIV/AIDS - Drug-susceptible Tuberculosis	2.0 (10)	0.9 (14)	2.7 (9)	12.7 (4)	0.6 (16)
Congenital heart anomalies	1.3 (11)	0.9 (13)	1.6 (11)	0.8 (15)	1.2 (12)
Neural tube defects	1.2 (12)	1.2 (10)	1.1 (14)	0.2 (29)	1.4 (10)
Other congenital birth defects	1.2 (13)	1.0 (12)	1.3 (12)	0.7 (16)	1.2 (13)
Sudden infant death syndrome	0.9 (14)	0.8 (16)	0.9 (15)	1.0 (13)	1.0 (14)
Syphilis	0.9 (15)	1.2 (11)	1.2 (13)	1.3 (11)	0.5 (17)
Invasive Non-typhoidal Salmonella (iNTS)	0.8 (16)	0.8 (15)	0.4 (22)	0.2 (23)	1.2 (11)
Digestive congenital anomalies	0.6 (17)	0.6 (17)	0.6 (19)	0.3 (20)	0.6 (15)
Other neonatal disorders	0.6 (18)	0.5 (19)	0.7 (17)	2.1 (7)	0.4 (19)
Neonatal preterm birth	0.5 (19)	0.6 (18)	0.6 (18)	1.1 (12)	0.2 (23)
Neonatal sepsis and other neonatal infections	0.4 (20)	0.2 (29)	0.7 (16)	0.6 (17)	0.2 (26)
Year: 1990					

Region->	Total	Central	Eastern	Southern	Western
Diarrheal diseases	25.4 (1)	20.0 (1)	20.2 (2)	28.6 (1)	32.7 (1)
Lower respiratory infections	21.3 (2)	19.4 (3)	21.6 (1)	26.2 (2)	21.2 (2)
Malaria	12.6 (3)	19.9 (2)	12.5 (3)	1.4 (14)	11.4 (3)
Measles	7.4 (4)	5.5 (5)	7.8 (4)	5.5 (3)	7.8 (4)
Protein-energy malnutrition	5.7 (5)	6.9 (4)	7.7 (5)	5.4 (4)	3.2 (6)
Meningitis	4.2 (6)	3.2 (8)	3.8 (6)	2.2 (10)	5.1 (5)
Whooping cough	2.9 (7)	3.4 (7)	3.0 (7)	1.8 (11)	2.6 (7)
Drug-susceptible tuberculosis	2.8 (8)	5.3 (6)	2.5 (8)	4.5 (5)	2.1 (8)
HIV/AIDS resulting in other diseases	1.5 (9)	0.9 (14)	2.4 (9)	3.1 (6)	0.6 (15)
Congenital heart anomalies	1.4 (10)	1.1 (12)	1.6 (11)	1.1 (15)	1.1 (10)
Neural tube defects	1.3 (11)	1.4 (9)	1.2 (14)	0.3 (27)	1.5 (9)
Other congenital birth defects	1.2 (12)	1.2 (10)	1.4 (12)	0.9 (16)	1.1 (11)
HIV/AIDS - Drug-susceptible Tuberculosis	1.0 (13)	0.7 (16)	1.7 (10)	2.4 (9)	0.3 (20)
Syphilis	1.0 (14)	1.1 (11)	1.3 (13)	2.4 (8)	0.5 (16)
Sudden infant death syndrome	1.0 (15)	0.9 (13)	1.0 (15)	1.5 (13)	0.9 (12)
Other neonatal disorders	0.7 (16)	0.6 (18)	0.7 (16)	2.5 (7)	0.5 (17)
Digestive congenital anomalies	0.6 (17)	0.7 (17)	0.6 (18)	0.4 (20)	0.6 (14)
Invasive Non-typhoidal Salmonella (iNTS)	0.6 (18)	0.7 (15)	0.3 (23)	0.4 (21)	0.8 (13)
Neonatal preterm birth	0.5 (19)	0.6 (19)	0.7 (17)	1.7 (12)	0.3 (22)
Neonatal sepsis and other neonatal infections	0.4 (20)	0.2 (29)	0.6 (20)	0.7 (18)	0.2 (25)

Notes: Rank for each cause within each year is shown in parentheses. Source: Global Burden of Disease Study, Institute for Health Metrics and Evaluation.

SUPPLEMENT 2: Information on data

Table S2. Sample size and missing data for the sample used for analyses of height-for-age: pooled and by survey

	Survey year	Total observations	Missing heigh-for-age	Insufficient data for postneonatal mortality	Remaining sample for survey fixed-effects	Single observation in neighborhood	Remaining sample for neighborhood fixed-effects	Single observation for sibling	Remaining sample for sibling fixed-effects
Pooled		444,925	27,326	218,182	199,417	2,874	196,543	141,603	57,814
Angola	2015–2016	5,211	220	4,756	235	53	182	233	2
Benin	1996	1,693	73	1,053	567	9	558	565	2
Benin	2001	3,516	92	540	2,884	5	2,879	2,037	847
Benin	2011–2012	9,651	1,232	1,280	7,139	10	7,129	4,899	2,240
Benin	2017–2018	9,424	152	3,889	5,383	20	5,363	3,531	1,852
Burkina Faso	1992–1993	3,992	545	330	3,117	7	3,110	2,178	939
Burkina Faso	1998–1999	3,933	355	503	3,075	2	3,073	2,263	812
Burkina Faso	2003	7,038	467	957	5,614	2	5,612	4,223	1,391
Burkina Faso	2010	5,350	129	3,627	1,594	57	1,537	1,406	188
Burundi	2010–2011	2,849	107	12	2,730	1	2,729	1,461	1,269
Burundi	2016–2017	4,843	39	322	4,482	4	4,478	2,680	1,802
Cameroon	1991	2,305	258	950	1,097	6	1,091	678	419
Cameroon	2004	2,677	160	886	1,631	52	1,579	1,038	593
Cameroon	2011	4,033	100	2,141	1,792	57	1,735	1,273	519
Cameroon	2018–2019	3,596	79	3,372	145	30	115	130	15
Central African Republic	1994–1995	1,649	136	1,221	292	4	288	278	14
Chad	2014–2015	8,460	424	5,519	2,517	20	2,497	1,710	807
Comoros	2012	2,234	224	1,719	291	26	265	261	30
Congo Democratic Republic	2007	3,034	351	2,076	607	7	600	410	197
Congo Democratic Republic	2013–2014	6,621	230	6,020	371	15	356	266	105
Cote d'Ivoire	1994	2,371	157	1,482	732	23	709	726	6
Cote d'Ivoire	1998–1999	1,351	150	800	401	7	394	339	62
Cote d'Ivoire	2011–2012	2,649	173	2,281	195	30	165	171	24
Ethiopia	1992	7,336	238	2,774	4,324	20	4,304	3,064	1,260
Ethiopia	1997	3,509	307	1,796	1,406	68	1,338	982	424
Ethiopia	2003	8,241	533	5,710	1,998	44	1,954	1,376	622
Ethiopia	2008	7,669	550	6,224	895	18	877	595	300
Ethiopia	2011	4,221	101	3,660	460	4	456	370	90
Gabon	2012	2,690	78	2,529	83	17	66	79	4
Ghana	1993–1994	1,315	84	713	518	68	450	514	4
Ghana	1998–1999	2,395	187	1,265	943	42	901	805	138
Ghana	2003	2,653	185	1,762	706	67	639	604	102
Ghana	2008	2,043	125	1,696	222	60	162	218	4
Ghana	2014	2,158	31	2,113	14	9	5	14	0
Guinea	1999	3,942	461	519	2,962	2	2,960	2,295	667
Guinea	2005	2,151	102	631	1,418	16	1,402	1,139	279
Guinea	2012	2,502	53	1,337	1,112	24	1,088	922	190
Guinea	2018	2,815	58	2,459	298	51	247	260	38
Kenya	2008–2009	4,250	166	2,182	1,902	29	1,873	1,275	627
Kenya	2014	15,450	395	11,267	3,788	145	3,643	3,006	782

	Survey year	Total observations	Missing heigh-for-age	Insufficient data for postneonatal mortality	Remaining sample for survey fixed-effects	Single observation in neighborhood	Remaining sample for neighborhood fixed-effects	Single observation for sibling	Remaining sample for sibling fixed-effects
Lesotho	2004–2005	1,167	126	107	934	87	847	757	177
Lesotho	2009–2010	1,324	60	48	1,216	70	1,146	958	258
Lesotho	2014	1,034	28	757	249	91	158	235	14
Liberia	2006–2007	3,651	244	182	3,225	5	3,220	2,266	959
Liberia	2013	2,522	92	775	1,655	21	1,634	1,204	451
Liberia	2019–2020	1,957	88	1,497	372	51	321	312	60
Madagascar	1997	2,128	199	1,329	600	12	588	576	24
Madagascar	2008–2009	4,412	261	3,942	209	43	166	201	8
Malawi	2000	7,522	362	25	7,135	0	7,135	4,757	2,378
Malawi	2004–2005	7,251	842	111	6,298	4	6,294	4,209	2,089
Malawi	2010	4,095	280	447	3,368	92	3,276	2,215	1,153
Malawi	2015–2016	4,325	155	2,537	1,633	121	1,512	1,400	233
Mali	1995–1996	3,223	260	254	2,709	5	2,704	2,635	74
Mali	2001	8,036	746	1,597	5,693	2	5,691	3,856	1,837
Mali	2006	9,244	484	3,622	5,138	4	5,134	3,460	1,678
Mali	2012–2013	3,852	196	2,511	1,145	37	1,108	814	331
Mali	2018	7,063	207	5,880	976	11	965	754	222
Mozambique	2011	7,582	194	6,160	1,228	16	1,212	1,070	158
Namibia	2000	2,331	101	1,770	460	12	448	355	105
Namibia	2006–2007	2,934	153	1,957	824	34	790	708	116
Namibia	2013	1,426	64	1,331	31	25	6	31	0
Niger	1992	4,209	638	585	2,986	1	2,985	1,838	1,148
Niger	1998	2,667	218	729	1,720	11	1,709	1,684	36
Niger	2012	4,318	449	2,518	1,351	32	1,319	1,024	327
Nigeria	1990	5,274	772	1,824	2,678	10	2,668	1,763	915
Nigeria	2003	3,733	304	757	2,672	21	2,651	1,770	902
Nigeria	2008	18,599	2,597	4,074	11,928	22	11,906	7,774	4,154
Nigeria	2013	21,368	1,507	9,147	10,714	36	10,678	7,096	3,618
Nigeria	2018	9,270	242	6,996	2,032	142	1,890	1,550	482
Rwanda	2005	2,983	99	29	2,855	3	2,852	1,603	1,252
Rwanda	2010–2011	3,347	17	20	3,310	6	3,304	2,101	1,209
Rwanda	2014–2015	2,840	37	306	2,497	23	2,474	1,849	648
Rwanda	2019–2020	3,055	7	977	2,071	33	2,038	1,594	477
Senegal	1992–1993	3,972	440	177	3,355	9	3,346	2,002	1,353
Senegal	2005	2,475	257	218	2,000	28	1,972	1,243	757
Senegal	2010–2011	3,330	337	749	2,244	24	2,220	1,516	728
Senegal	2012–2013	4,833	157	1,951	2,725	3	2,722	1,884	841
Senegal	2018	4,942	102	4,625	215	11	204	203	12
Senegal	2019	4,442	107	4,215	120	15	105	118	2
Sierra Leone	2008	1,812	119	15	1,678	22	1,656	1,190	488
Sierra Leone	2013	3,802	395	48	3,359	10	3,349	2,449	910
Sierra Leone	2019	3,482	293	692	2,497	42	2,455	1,909	588
Swaziland	2006–2007	1,678	81	89	1,508	24	1,484	1,126	382
Tanzania	1999	2,022	68	1,123	831	2	829	548	283
Tanzania	2009–2010	5,584	243	4,260	1,081	14	1,067	699	382

	Survey year	Total observations	Missing heigh-for-age	Insufficient data for postneonatal mortality	Remaining sample for survey fixed-effects	Single observation in neighborhood	Remaining sample for neighborhood fixed-effects	Single observation for sibling	Remaining sample for sibling fixed-effects
Tanzania	2015–2016	7,230	166	6,949	115	15	100	115	0
Togo	1988	1,165	61	99	1,005	14	991	985	20
Togo	1998	2,441	120	487	1,834	29	1,805	1,806	28
Togo	2013–2014	2,589	41	1,412	1,136	34	1,102	928	208
Uganda	2000–2001	4,517	485	655	3,377	5	3,372	1,822	1,555
Uganda	2006	1,959	96	343	1,520	27	1,493	849	671
Uganda	2011	1,703	88	603	1,012	78	934	630	382
Uganda	2016	3,545	72	3,047	426	85	341	346	80
Zambia	2007	4,356	236	2,583	1,537	14	1,523	1,124	413
Zambia	2013–2014	9,818	500	7,276	2,042	51	1,991	1,653	389
Zambia	2018–2019	7,189	275	6,847	67	17	50	67	0
Zimbabwe	1999	2,408	242	1,752	414	18	396	358	56
Zimbabwe	2005–2006	3,483	323	2,414	746	24	722	669	77
Zimbabwe	2010–2011	3,461	281	2,594	586	25	561	538	48
Zimbabwe	2015	4,125	205	3,785	135	20	115	133	2

Notes: Total observations refer to children under five years born to an interviewed respondent, both present in the interviewed household. Insufficient data for postneonatal mortality rate refers to children in neighborhoods that were not exposed to at least 5 deaths and 600 person-months (ie, 50 person-years). Those with missing information on height-for-age were excluded before counting observations with missing postneonatal mortality rate. Single observations within neighborhoods were excluded when using neighborhood fixed-effects models and children without a sibling with valid data were excluded when using sibling fixed-effects models.

Table S3. Sample size and missing data for the sample used for analyses of school attendance: pooled and by survey

	Survey year	Total observations	Missing school attendance	Insufficient data for postneonatal mortality	Remaining sample for survey fixed-effects	Single observation in neighborhood	Remaining sample for neighborhood fixed-effects	Single observation for sibling	Remaining sample for sibling fixed-effects
Pooled		740,671	851	297,415	442,405	1,246	441,159	118,575	323,830
Angola	2015–2016	11,215	0	8,953	2,262	44	2,218	825	1,437
Benin	2017–2018	11,826	0	2,952	8,874	9	8,865	2,528	6,346
Burkina Faso	2003	10,811	10	1,172	9,629	1	9,628	2,213	7,416
Burkina Faso	2010	15,150	15	3,508	11,627	5	11,622	2,822	8,805
Burundi	2010–2011	7,115	4	11	7,100	0	7,100	1,137	5,963
Burundi	2016–2017	15,701	0	120	15,581	0	15,581	2,114	13,467
Cameroon	2004	6,895	13	3,662	3,220	26	3,194	1,177	2,043
Cameroon	2011	9,966	14	3,870	6,082	13	6,069	1,861	4,221
Cameroon	2018–2019	9,060	0	4,949	4,111	14	4,097	1,341	2,770
Chad	2014–2015	21,030	38	14,265	6,727	7	6,720	1,998	4,729
Comoros	2012	3,724	14	3,081	629	36	593	391	238
Congo Democratic Republic	2007	7,656	6	6,202	1,448	0	1,448	478	970
Congo Democratic Republic	2013–2014	15,886	17	13,501	2,368	7	2,361	675	1,693
Cote d'Ivoire	2011–2012	5,726	8	5,201	517	16	501	352	165
Ethiopia	1992	11,969	4	6,078	5,887	17	5,870	1,732	4,155
Ethiopia	1997	12,574	27	5,099	7,448	18	7,430	1,931	5,517
Ethiopia	2003	13,888	3	6,948	6,937	15	6,922	1,925	5,012
Ethiopia	2008	14,005	0	9,322	4,683	58	4,625	1,554	3,129
Ethiopia	2011	8,124	0	5,824	2,300	35	2,265	768	1,532
Gabon	2012	5,048	10	4,716	322	14	308	278	44
Ghana	2003	4,073	4	2,196	1,873	36	1,837	748	1,125
Ghana	2008	3,158	1	1,946	1,211	60	1,151	540	671
Ghana	2014	6,497	0	5,221	1,276	38	1,238	749	527
Guinea	2005	6,566	12	1,233	5,321	1	5,320	1,598	3,723
Guinea	2012	7,171	2	1,871	5,298	2	5,296	1,645	3,653
Guinea	2018	7,937	0	3,835	4,102	14	4,088	1,678	2,424
Kenya	2008–2009	6,619	7	3,747	2,865	21	2,844	1,002	1,863
Kenya	2014	28,925	10	17,167	11,748	119	11,629	3,604	8,144
Lesotho	2004–2005	4,562	2	1,324	3,236	20	3,216	1,182	2,054
Lesotho	2014	3,548	0	108	3,440	13	3,427	1,217	2,223
Liberia	2006–2007	4,422	42	281	4,099	2	4,097	1,273	2,826
Liberia	2013	6,085	8	146	5,931	1	5,930	1,840	4,091
Liberia	2019–2020	5,537	0	1,227	4,310	9	4,301	1,530	2,780
Madagascar	2008–2009	15,244	31	12,496	2,717	17	2,700	1,086	1,631
Malawi	2000	9,411	7	176	9,228	1	9,227	2,037	7,191
Malawi	2010	20,632	12	743	19,877	1	19,876	3,943	15,934
Malawi	2015–2016	22,073	0	1,704	20,369	5	20,364	4,183	16,186
Mali	2001	11,430	27	2,829	8,574	0	8,574	1,970	6,604
Mali	2006	13,304	39	2,876	10,389	2	10,387	2,145	8,244
Mali	2012–2013	10,413	0	2,686	7,727	2	7,725	1,753	5,974

	Survey year	Total observations	Missing school attendance	Insufficient data for postneonatal mortality	Remaining sample for survey fixed-effects	Single observation in neighborhood	Remaining sample for neighborhood fixed-effects	Single observation for sibling	Remaining sample for sibling fixed-effects
Mali	2018	9,853	0	5,739	4,114	19	4,095	1,318	2,796
Mozambique	2011	10,108	2	8,359	1,747	22	1,725	813	934
Namibia	2000	3,240	21	2,859	360	16	344	200	160
Namibia	2006–2007	4,190	23	3,234	933	32	901	456	477
Namibia	2013	3,551	7	2,778	766	55	711	489	277
Niger	2012	11,874	14	5,711	6,149	8	6,141	1,502	4,647
Nigeria	2003	5,265	11	2,104	3,150	18	3,132	1,014	2,136
Nigeria	2008	25,096	60	7,318	17,718	14	17,704	5,060	12,658
Nigeria	2013	30,249	58	7,061	23,130	8	23,122	6,041	17,089
Nigeria	2018	34,155	0	11,003	23,152	46	23,106	5,884	17,268
Rwanda	2005	8,186	18	76	8,092	0	8,092	1,611	6,481
Rwanda	2010–2011	9,920	6	0	9,914	0	9,914	1,731	8,183
Rwanda	2014–2015	9,986	5	3	9,978	0	9,978	1,721	8,257
Rwanda	2019–2020	10,446	0	360	10,086	0	10,086	2,225	7,861
Senegal	2005	10,149	36	976	9,137	4	9,133	2,071	7,066
Senegal	2012–2013	6,084	0	525	5,559	1	5,558	1,150	4,409
Sierra Leone	2008	4,761	23	94	4,644	0	4,644	1,456	3,188
Sierra Leone	2013	9,835	7	29	9,799	1	9,798	3,107	6,692
Sierra Leone	2019	9,077	0	501	8,576	2	8,574	2,999	5,577
Swaziland	2006–2007	2,751	5	1,358	1,388	42	1,346	674	714
Tanzania	2007–2008	7,423	65	4,472	2,886	20	2,866	658	2,228
Tanzania	2009–2010	7,996	42	5,413	2,541	15	2,526	666	1,875
Tanzania	2015–2016	10,257	0	8,084	2,173	34	2,139	742	1,431
Togo	2013–2014	7,508	3	3,221	4,284	15	4,269	1,545	2,739
Uganda	2006	7,507	7	1,576	5,924	2	5,922	1,184	4,740
Uganda	2016	14,619	0	3,532	11,087	8	11,079	2,657	8,430
Zambia	2007	5,471	3	3,459	2,009	11	1,998	743	1,266
Zambia	2013–2014	14,483	34	9,096	5,353	24	5,329	1,879	3,474
Zambia	2018–2019	11,829	0	8,482	3,347	26	3,321	1,305	2,042
Zimbabwe	1999	4,032	10	3,938	84	20	64	74	10
Zimbabwe	2005–2006	5,280	4	4,659	617	28	589	463	154
Zimbabwe	2010–2011	5,160	0	4,046	1,114	34	1,080	611	503
Zimbabwe	2015	5,354	0	4,103	1,251	22	1,229	703	548

Notes: Total observations refer to children aged 7–16 years born to an interviewed respondent, both present in the interviewed household. Insufficient data for postneonatal mortality rate refers to children in neighborhoods that were not exposed to at least 5 deaths and 600 person-months (ie, 50 person-years). Those with missing information on school attendance were excluded before counting observations with missing postneonatal mortality rate. Single observations within neighborhoods were excluded when using neighborhood fixed-effects models and children without a sibling with valid data were excluded when using sibling fixed-effects models.

Table S4. Linear regressions comparing those with and without missing data for height-for-age and postneonatal mortality rate linked to the height-for-age sample

Outcome->	Missing height-for-age				Missing Postneonatal mortality rate			
Fixed-effects->	None	Survey	Neighborhood	Sibling	None	Survey	Neighborhood	Sibling
Independent variables								
Child's age (months)	.00015***	.000098***	.000095***	.000021	.00019***	-.00021***	-.00031***	-.00042***
	[.000082, .00021]	[.000048, .00015]	[.000042, .00015]	[-.000072, .00011]	[.000063, .00032]	[-.00031, -.0001]	[-.00041, -.00021]	[-.00059, -.00025]
Mother's age (years)	-.00072***	-.00071***	-.00059***	-.00008	-.0008***	-.00043***	-.00025***	.0053***
	[-.00089, -.00055]	[-.00082, -.00061]	[-.0007, -.00047]	[-.0012, .0011]	[-.0011, -.00054]	[-.00062, -.00024]	[-.00038, -.00013]	[.0033, .0074]
Number of siblings	-.0071***	-.0018***	-.0016***		-.0064***	.00059*	-.0011***	
	[-.0078, -.0064]	[-.0021, -.0015]	[-.002, -.0013]		[-.0073, -.0055]	[-.000028, .0012]	[-.0015, -.00073]	
Child's year of birth	-.0016***	-.001***	-.00075**	-.00018	.018***	.0038***	.0037***	.0049***
	[-.0018, -.0014]	[-.0016, -.00045]	[-.0014, -.00013]	[-.0013, .00094]	[.017, .018]	[.0026, .0051]	[.0026, .0049]	[.0028, .0069]
Mother's education (years)	-.000048	-.00055***	.00022		.0075***	-.0068***	.00031**	
	[-.00044, .00035]	[-.00079, -.00031]	[-.000046, .00049]		[.0067, .0083]	[-.0075, -.0062]	[5.1e-06, .00061]	
Rural resident	.0077***	-.0053***			.051***	.084***		
	[.0021, .013]	[-.0074, -.0032]			[.041, .061]	[.076, .091]		
Postneonatal mortality rate (per 100)	.0027***	.0014***	-.000013	-.00052				
	[.0023, .003]	[.00097, .0017]	[-.00062, .00059]	[-.0021, .001]				
Height-for-age z-score					.014***	-.0038***	.00026	-.0014

Notes: ***p<0.01; **p<0.05; *p<0.1. Linear regression coefficients are shown. Each coefficient was obtained from a separate regression and was unadjusted, except for fixed-effects indicated in the second row. 95% confidence intervals (shown in brackets) and p-values were adjusted for clustering within primary sampling units.

Table S5. Linear regressions comparing those with and without missing data for school attendance and postneonatal mortality rate linked to the school attendance sample

Outcome->	Missing school attendance				Missing Postneonatal mortality rate			
Fixed-effects->	None	Survey	Neighborhood	Sibling	None	Survey	Neighborhood	Sibling
Independent variables								
Child's age (months)	7.8e-06	5.1e-06***	5.3e-06***	8.0e-06***	-.00038***	-.00041***	-.0003***	-.0003***
	[-7.0e-06, .000023]	[2.2e-06, 8.1e-06]	[2.2e-06, 8.4e-06]	[3.8e-06, .000012]	[-.00044, -.00033]	[-.00046, -.00037]	[-.00035, -.00026]	[-.00036, -.00024]
Mother's age (years)	.00021***	-.000016**	-.000014*	-.000093***	-.0017***	-.00035***	.00023***	.0036***
	[.00007, .00034]	[-.00003, -2.6e-06]	[-.000028, 4.2e-07]	[-.00014, -.000043]	[-.002, -.0014]	[-.00057, -.00014]	[.00013, .00034]	[.0029, .0044]
Number of siblings	.002***	1.5e-06	-5.7e-06		-.00084	.00096**	-.00051***	
	[.0015, .0025]	[-.000036, .000039]	[-.000047, .000036]		[-.0019, .00023]	[.00018, .0017]	[-.00079, -.00022]	
Child's year of birth	-.0023***	-.000068***	-.000068***	-.000097***	-.0005	.0053***	.0036***	.0035***
	[-.0025, -.0021]	[-.0001, -.000032]	[-.0001, -.000031]	[-.00015, -.000047]	[-.0012, .00022]	[.0047, .0059]	[.0031, .0041]	[.0028, .0043]
Mother's education (years)	-.0022***	-.000023*	-1.6e-06		.0017***	-.0089***	.00018	
	[-.0026, -.0018]	[-.000049, 2.1e-06]	[-.00004, .000037]		[.00085, .0026]	[-.0096, -.0082]	[-.000039, .00039]	
Rural resident	.0045	-.000033			.05***	.1***		
	[-.00086, .0098]	[-.00026, .0002]			[.039, .06]	[.096, .11]		
Postneonatal mortality rate (per 100)	.000018***	-.000014	-.000019	-7.2e-06				
	[5.4e-06, .00003]	[-.000035, 6.5e-06]	[-.000044, 5.9e-06]	[-.000045, .000031]				
School attendance					-.036***	-.067***	.002*	.00087

Notes: ***p<0.01; **p<0.05; *p<0.1. Linear regression coefficients are shown. Each coefficient was obtained from a separate regression and was unadjusted, except for fixed-effects indicated in the second row. 95% confidence intervals (shown in brackets) and p-values were adjusted for clustering within primary sampling units.

Figure S1. Map of the study area: Neighborhoods in the analyses of school attendance

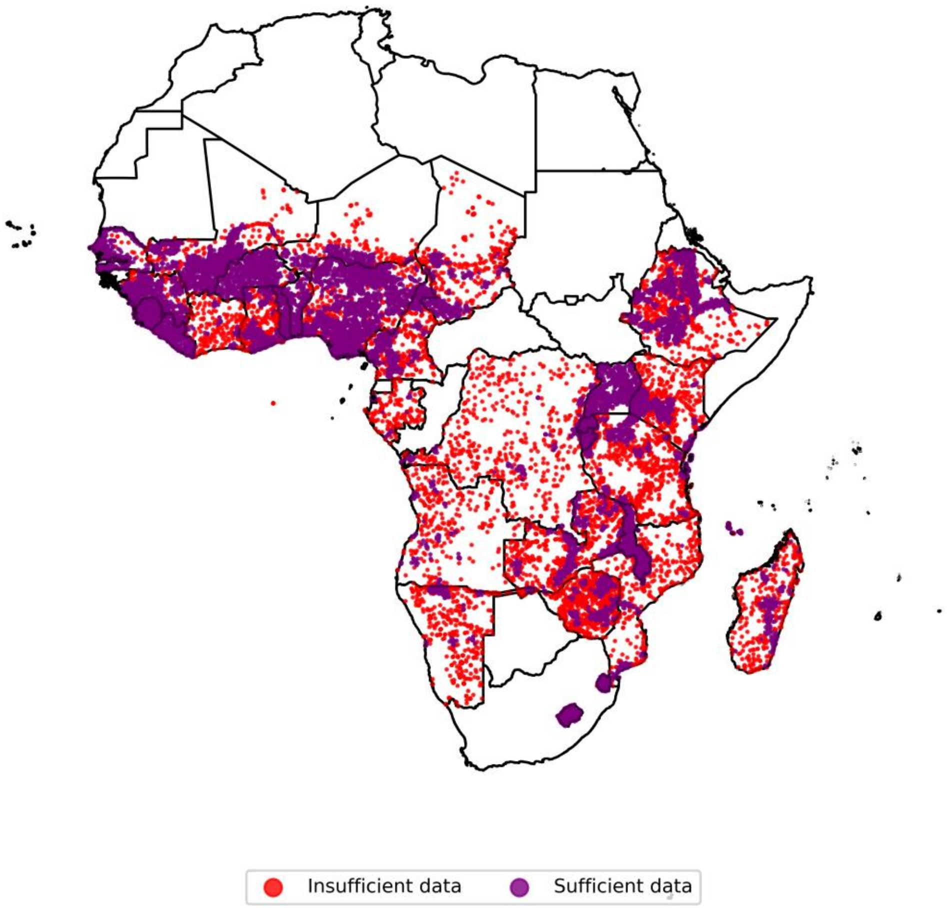

Notes: Markers indicate neighborhoods (ie, primary sampling units). Insufficient data refers to neighborhoods where no children were exposed to at least 5 postneonatal deaths and 600 person-months over the period of infancy in a 50 kilometer radius and were therefore excluded from analyses.

Figure S2. Histogram for height-for-age and postneonatal mortality rate

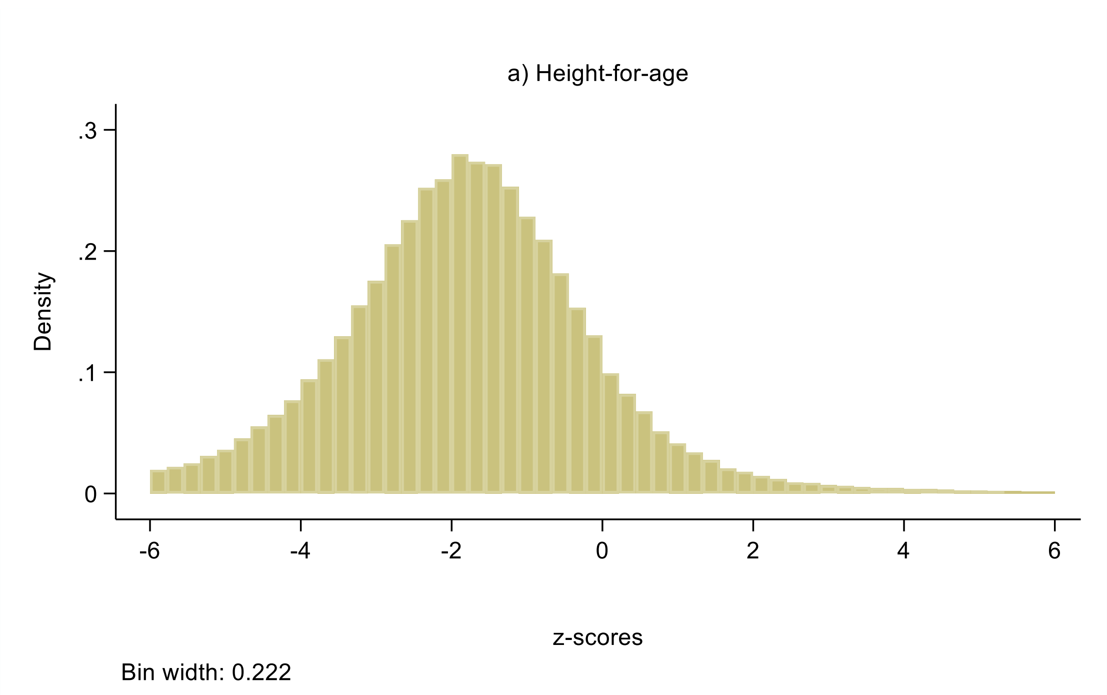

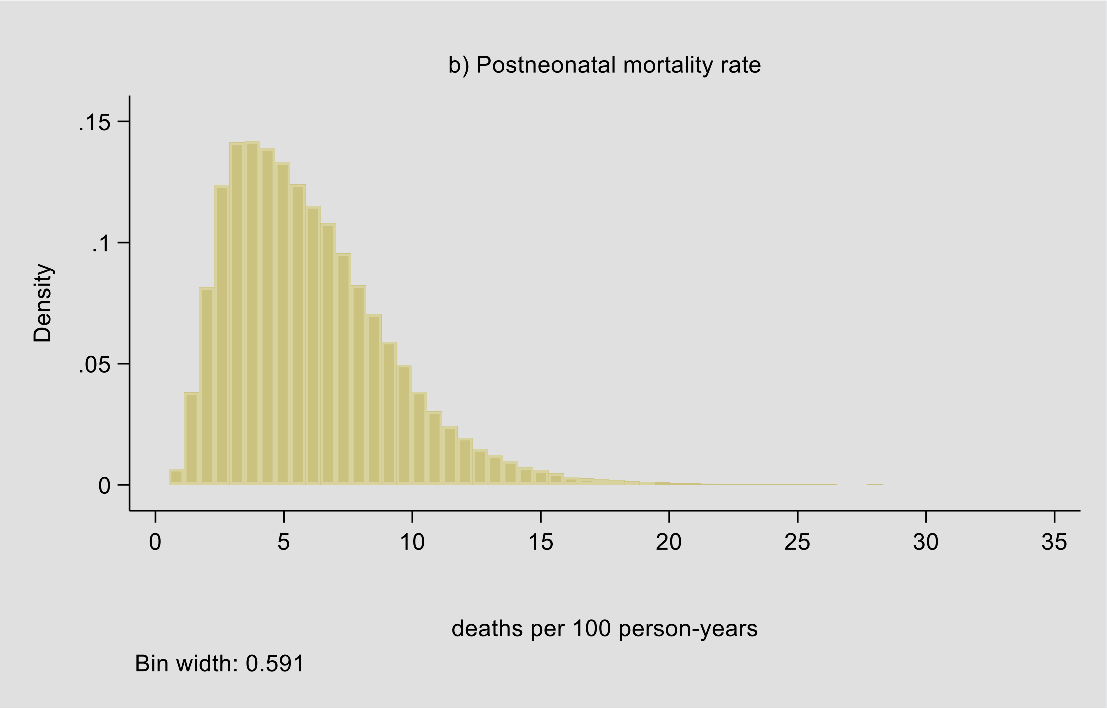

Notes: Height-for-age shows z-score deviations from the median of the 2006 World Health Organization growth standard. Postneonatal mortality rate was restricted to 50 kilometer radius and linked to the period of infancy and refers to the pooled samples for both outcomes (before excluding neighborhoods with a single observation and children without a sibling with valid data). The number of bins used was determined as 10*ln(N)/ln(10) where N is number of valid observations for each measure.

SUPPLEMENT 3: Results including survey fixed-effects models

Table S6. Descriptive statistics including for full sample used with survey fixed-effects models

Sample for outcome->	Height-for-age			School attendance		
Sample for fixed-effects->	Survey	Neighborhood	Sibling	Survey	Neighborhood	Sibling
Variables						
Height-for-age z-score	-1.8	-1.8	-1.75			
	[-1.81, -1.8]	[-1.81, -1.8]	[-1.77, -1.7]			
School attendance				.761	.761	.759
				[.757, .77]	[.757, .77]	[.755, .76]
Postneonatal mortality rate (per 100)	5.24	5.25	5.1	6.35	6.35	6.37
	[5.2, 5.3]	[5.2, 5.3]	[5.05, 5.2]	[6.31, 6.4]	[6.31, 6.4]	[6.33, 6.4]
Mother's education (years)	3.32	3.29	3.1	3.34	3.33	3.1
	[3.27, 3.4]	[3.24, 3.3]	[3.03, 3.2]	[3.29, 3.4]	[3.29, 3.4]	[3.05, 3.1]
Mother's age at birth (years)	27.3	27.3	27	26	26	26.4
	[27.3, 27]	[27.3, 27]	[26.9, 27]	[26, 26]	[26, 26]	[26.4, 26]
Birth interval (months)	38.5	38.4	30.2	35.9	35.9	33.7
	[38.4, 39]	[38.3, 39]	[30.1, 30]	[35.8, 36]	[35.8, 36]	[33.6, 34]
Firstborn	.197	.197	.118	.218	.218	.148
	[.195, .2]	[.195, .2]	[.115, .12]	[.217, .22]	[.216, .22]	[.147, .15]
Birth order	3.8	3.81	3.94	3.46	3.46	3.71
	[3.79, 3.8]	[3.8, 3.8]	[3.91, 4]	[3.45, 3.5]	[3.45, 3.5]	[3.69, 3.7]
Number of siblings	4.22	4.23	4.6	5.9	5.91	6.35
	[4.2, 4.2]	[4.21, 4.2]	[4.57, 4.6]	[5.88, 5.9]	[5.89, 5.9]	[6.33, 6.4]
Age (months)	34.2	34.1	35.3	135	135	138
	[34.1, 34]	[34.1, 34]	[35.2, 35]	[135, 135]	[135, 135]	[138, 138]
Female	.497	.497	.494	.489	.489	.485
	[.494, .5]	[.494, .5]	[.49, .5]	[.487, .49]	[.487, .49]	[.483, .49]
Twin	.029	.0294	.0146	.0255	.0255	.0156
	[.0281, .03]	[.0284, .03]	[.0136, .016]	[.0248, .026]	[.0249, .026]	[.0152, .016]
Mother had education	.49	.486	.48	.502	.501	.485
	[.484, .5]	[.48, .49]	[.471, .49]	[.496, .51]	[.496, .51]	[.479, .49]
Child's year of birth	2004	2004	2004	2000	2000	2000
	[2003, 2004]	[2003, 2004]	[2003, 2004]	[2000, 2000]	[2000, 2000]	[1999, 2000]
Rural resident	.695	.697	.719	.708	.708	.723
	[.688, .7]	[.69, .7]	[.71, .73]	[.702, .71]	[.702, .71]	[.716, .73]
Observations	199,417	196,543	57,814	442,405	441,159	323,830
Fixed-effects	100	23,257	28,603	73	25,719	127,515
Surveys	100	100	96	73	73	73
Countries	33	33	33	32	32	32

Notes: 95% confidence intervals (shown in brackets) were adjusted for clustering within primary sampling units. Descriptive statistics for birth interval excluded firstborns (who were however included in the analyses using dummy variable adjustment). Separate samples were used for each outcome (height-for-age and school attendance) indicated in the first row of each column. Analyses using neighborhood fixed-effects models excluded neighborhoods with a single observation and sibling fixed-effects models excluded children without a sibling with valid data: the same exclusions were applied in this table indicated in the second row of each column. Postneonatal mortality rate refers to deaths per 100 person-years (ie, deaths per 1200 person months of exposure). Postneonatal mortality rate was restricted to 50 kilometer radius and linked to the period of infancy.

Table S7. Results from linear regression models: Results including survey fixed-effects models

Outcome->	Height-for-age z-score			School attendance		
Fixed-effects->	Survey	Neighborhood	Sibling	Survey	Neighborhood	Sibling
Independent variables						
Postneonatal mortality rate (per 100)	-.06***	-.015***	-.026**	-.021***	-.0016***	-.0007
	[-.068, -.052]	[-.026, -.0046]	[-.05, -.002]	[-.022, -.019]	[-.0028, -.00046]	[-.0025, .0011]
Postneonatal mortality rate (per 100) squared	.0019***	.0011**	.0021**	.00081***	.00034***	.00028***
	[.0012, .0026]	[.00021, .0019]	[.00033, .0039]	[.00068, .00094]	[.00023, .00044]	[.00011, .00044]
Firstborn	.22***	.2***	.24***	.00087	-.000099	-.015***
	[.19, .25]	[.17, .24]	[.14, .33]	[-.0035, .0053]	[-.0041, .0039]	[-.022, -.0085]
Birth interval (months)	.0047***	.0047***	.007***	.00016***	.00006*	-.00024***
	[.0043, .0051]	[.0042, .0051]	[.005, .009]	[.00009, .00023]	[-2.0e-06, .00012]	[-.00038, -.000098]
Birth order	-.22***	-.23***	-1.1***	.01***	.00075	-.015***
	[-.24, -.2]	[-.25, -.21]	[-1.2, -.98]	[.0087, .012]	[-.00066, .0022]	[-.021, -.0095]
Age (months)	-.055***	-.058***	-.087***	.014***	.014***	.014***
	[-.058, -.052]	[-.061, -.054]	[-.095, -.08]	[.014, .015]	[.013, .014]	[.014, .015]
Age (months) squared	.0007***	.00072***	.00073***	-.000049***	-.000049***	-.000053***
	[.00066, .00074]	[.00068, .00077]	[.00063, .00083]	[-.00005, -.000048]	[-.00005, -.000048]	[-.000055, -.000052]
Female	.16***	.16***	.17***	-.02***	-.022***	-.028***
	[.14, .17]	[.14, .17]	[.13, .21]	[-.023, -.018]	[-.024, -.02]	[-.031, -.024]
Mother's age at birth (years)	.065***	.047***		.0032***	-.0017**	
	[.055, .074]	[.037, .057]		[.0013, .0051]	[-.0034, -.000035]	
Mother's age at birth (years) squared	-.0007***	-.0005***		-.000054***	.000022	
	[-.00085, -.00054]	[-.00067, -.00032]		[-.000089, -.000018]	[-9.8e-06, .000053]	
Mother's education (years)	.073***	.035***		.024***	.0091***	
	[.071, .076]	[.032, .038]		[.023, .024]	[.0087, .0095]	
Number of siblings	.18***	.2***		-.016***	-.0048***	
	[.16, .2]	[.18, .22]		[-.018, -.015]	[-.0059, -.0038]	
Twin	-.58***	-.62***	-.7***	.025***	.0095**	.011
	[-.64, -.53]	[-.68, -.56]	[-.88, -.52]	[.017, .033]	[.0018, .017]	[-.0032, .026]
Constant	-1.7***	-1.8***	-1.7***	.8***	.76***	.76***
	[-1.7, -1.7]	[-1.8, -1.8]	[-1.8, -1.7]	[.79, .8]	[.76, .76]	[.75, .76]
R squared	0.093	0.267	0.699	0.222	0.433	0.706
Observations	199,417	196,543	57,814	442,405	441,159	323,830

Notes: ***p<0.01; **p<0.05; *p<0.1. Linear regression coefficients are shown. Postneonatal mortality rate was centered around percentile 25 (considering the pooled samples for both outcomes) and all covariates were mean-centered (using means for all valid observations in each analysis): therefore, the constant shows the mean outcome when covariates were at their means and postneonatal mortality rate was at percentile 25, and the coefficient of the linear term for postneonatal mortality rate shows the marginal effect at percentile 25. 95% confidence intervals (shown in brackets) and p-values were adjusted for clustering within primary sampling units. Postneonatal mortality rate per 100 person-years within a 50 kilometer radius was linked to the period of infancy.

Figure S3. Marginal effects of postneonatal mortality rate on height-for-age across the distribution of postneonatal mortality rate: Results including survey fixed-effects models

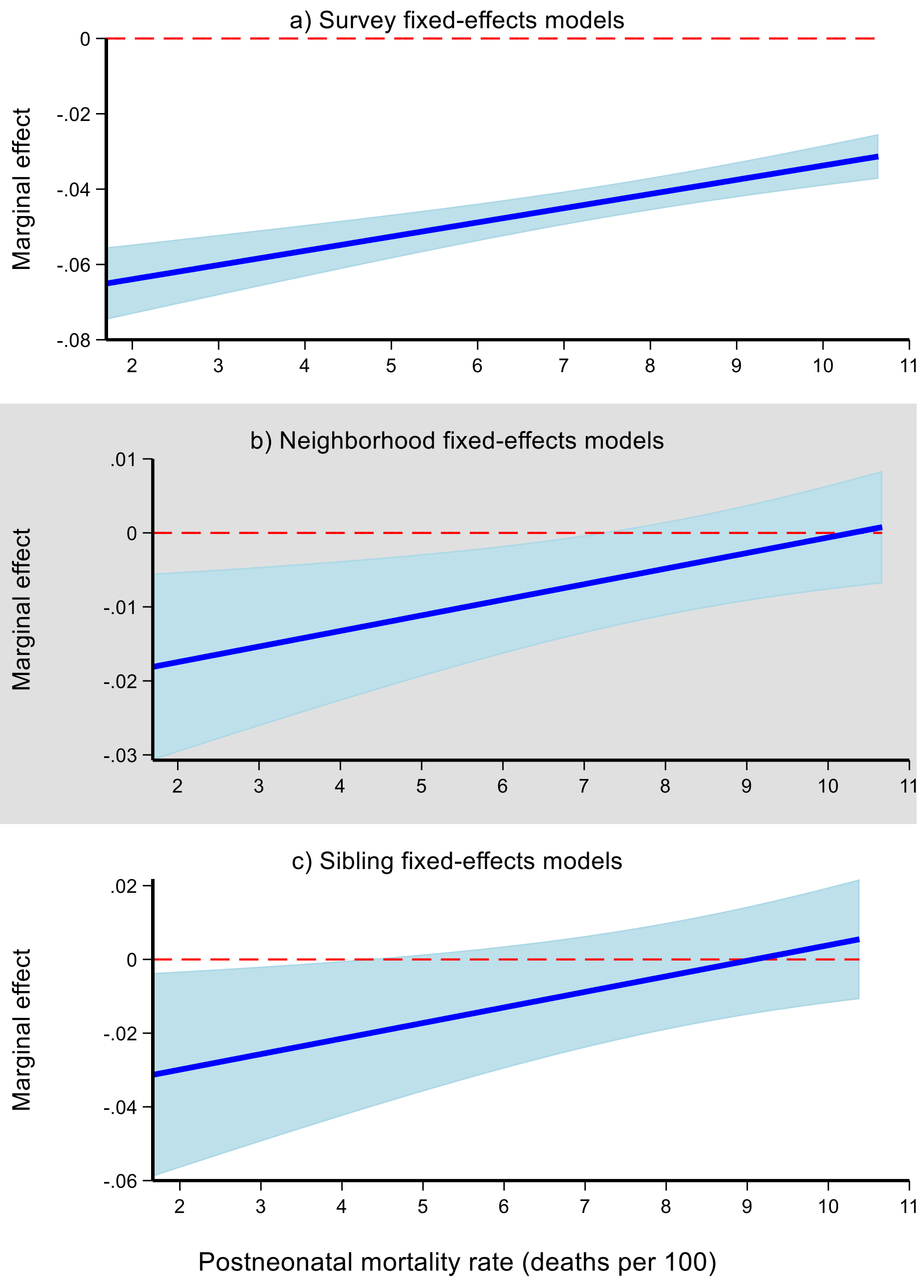

Notes: The values on the y-axis indicate marginal effects: that is, change in the outcome for a single death increase in postneonatal mortality rate per 100 person-years. Since the relationship was nonlinear, the marginal effects vary across the distribution of postneonatal mortality rate. The marginal effects were obtained using the partial derivative of the regression equation with respect to postneonatal mortality rate. The plot was restricted to postneonatal mortality rate between the 5th and 95th percentile (considering the pooled samples for both outcomes). 95% confidence intervals adjusted for clustering within primary sampling units are shown. Postneonatal mortality rate per 100 person-years within a 50 kilometer radius was linked to the period of infancy.

Figure S4. Marginal effects of postneonatal mortality rate on school attendance across the distribution of postneonatal mortality rate: Results including survey fixed-effects models

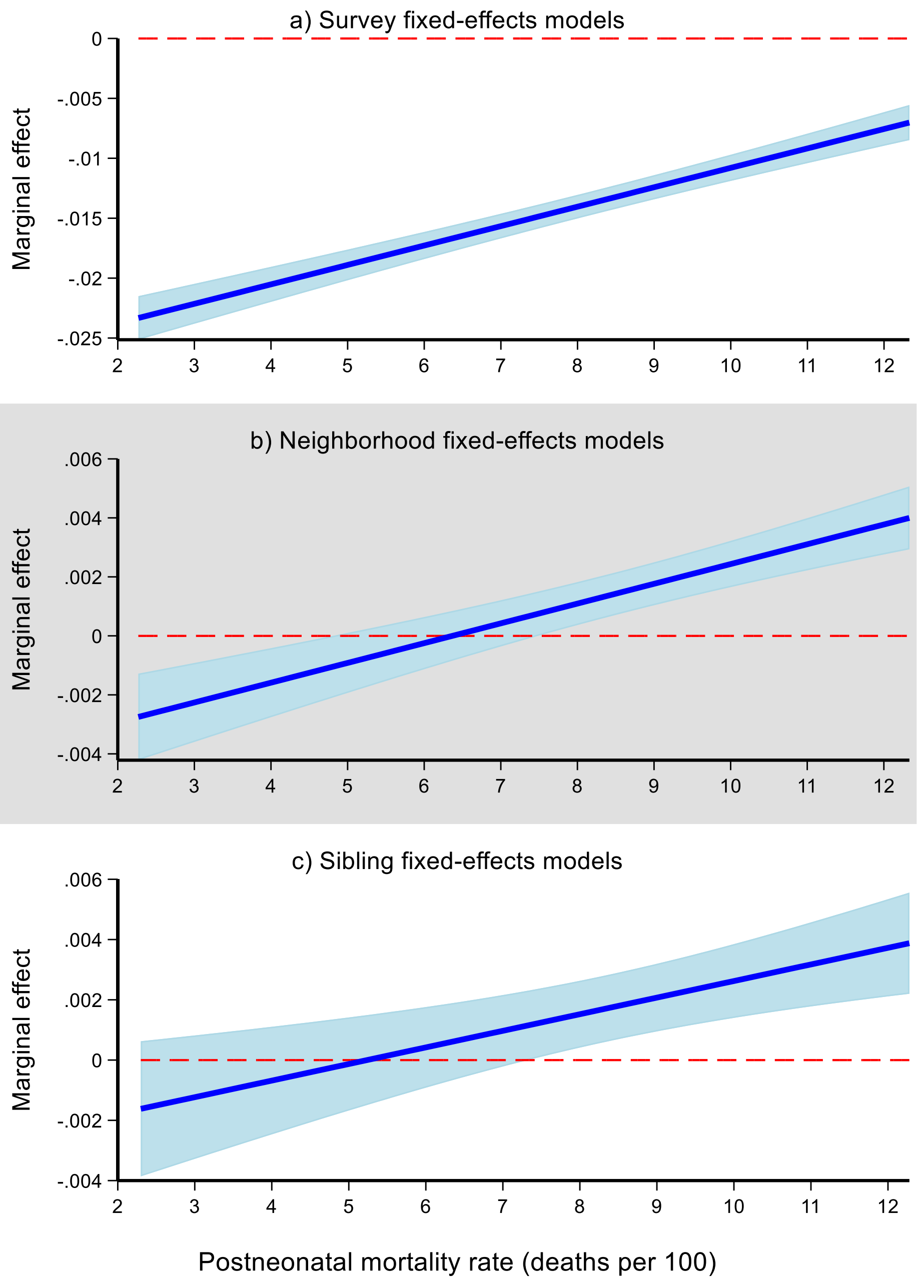

Notes: The values on the y-axis indicate marginal effects: that is, change in the outcome for a single death increase in postneonatal mortality rate per 100 person-years. Since the relationship was nonlinear, the marginal effects vary across the distribution of postneonatal mortality rate. The marginal effects were obtained using the partial derivative of the regression equation with respect to postneonatal mortality rate. The plot was restricted to postneonatal mortality rate between the 5th and 95th percentile (considering the pooled samples for both outcomes). 95% confidence intervals adjusted for clustering within primary sampling units are shown. Postneonatal mortality rate per 100 person-years within a 50 kilometer radius was linked to the period of infancy.

Table S8. Marginal effects of postneonatal mortality rate on height-for-age and school attendance at different percentiles of postneonatal mortality rate: Results including survey fixed-effects models

Outcome->	Height-for-age z-score			School attendance		
Fixed-effects->	Survey	Neighborhood	Sibling	Survey	Neighborhood	Sibling
Percentiles of postneonatal mortality rate (per 100)						
p1 (1.4)	-.067***	-.019***	-.033**	-.025***	-.0032***	-.002
	[-.077, -.057]	[-.033, -.0058]	[-.062, -.0042]	[-.027, -.023]	[-.0048, -.0016]	[-.0045, .00043]
p5 (2.0)	-.065***	-.018***	-.031**	-.023***	-.0027***	-.0016
	[-.075, -.055]	[-.031, -.0055]	[-.059, -.0037]	[-.025, -.022]	[-.0042, -.0013]	[-.0039, .00063]
p10 (2.5)	-.063***	-.017***	-.03**	-.023***	-.0024***	-.0013
	[-.073, -.054]	[-.029, -.0052]	[-.056, -.0032]	[-.024, -.021]	[-.0038, -.001]	[-.0034, .00076]
p25 (3.6)	-.06***	-.015***	-.026**	-.021***	-.0016***	-.0007
	[-.068, -.052]	[-.026, -.0046]	[-.05, -.002]	[-.022, -.019]	[-.0028, -.00046]	[-.0025, .0011]
p50 (5.4)	-.054***	-.012***	-.019*	-.018***	-.00038	.00033
	[-.06, -.048]	[-.02, -.0032]	[-.039, .00042]	[-.019, -.016]	[-.0013, .00053]	[-.001, .0017]
p75 (7.8)	-.046***	-.0073**	-.01	-.014***	.0012***	.0016***
	[-.05, -.041]	[-.014, -.00056]	[-.026, .0053]	[-.015, -.013]	[.00047, .0019]	[.0005, .0027]
p90 (10.2)	-.037***	-.0026	-.0013	-.0098***	.0028***	.0029***
	[-.042, -.033]	[-.0091, .0039]	[-.016, .013]	[-.011, -.0087]	[.002, .0037]	[.0016, .0043]
p95 (11.9)	-.031***	.00078	.0055	-.007***	.004***	.0039***
	[-.037, -.025]	[-.0068, .0084]	[-.011, .022]	[-.0085, -.0056]	[.0029, .0051]	[.0022, .0056]
p99 (15.8)	-.017***	.0089	.022*	-.00067	.0066***	.006***
	[-.027, -.0063]	[-.0036, .021]	[-.0039, .047]	[-.003, .0017]	[.0049, .0084]	[.0033, .0088]

Notes: ***p<0.01; **p<0.05; *p<0.1. Change in outcome for a single increase in postneonatal mortality rate per 100 person-years are shown at different percentiles of postneonatal mortality rate. Since the relationship was nonlinear, the marginal effects vary across the distribution of postneonatal mortality rate. The marginal effects were obtained using the partial derivative of the regression equation with respect to postneonatal mortality rate. The level of postneonatal mortality rate per 100 at each of the percentile is shown in parentheses (considering the pooled samples for both outcomes). 95% confidence intervals adjusted for clustering within primary sampling units are shown in brackets. Postneonatal mortality rate per 100 person-years within a 50 kilometer radius was linked to the period of infancy.

SUPPLEMENT 4: Sensitivity analyses

Table S9. Results from linear regression models: Postneonatal mortality rate with distance restricted to 25 kilometers

Outcome->	Height-for-age z-score			School attendance		
Fixed-effects->	Survey	Neighborhood	Sibling	Survey	Neighborhood	Sibling
Independent variables						
Postneonatal mortality rate (per 100)	-.063***	-.014*	-.019	-.016***	-.00082	-.001
	[-.074, -.052]	[-.029, .001]	[-.054, .015]	[-.018, -.014]	[-.0023, .00066]	[-.0034, .0013]
Postneonatal mortality rate (per 100) squared	.0018***	.0013**	.0019	.00054***	.00023***	.00023**
	[.00081, .0027]	[.00023, .0024]	[-.00057, .0044]	[.00039, .00068]	[.00011, .00034]	[.000041, .00042]
Firstborn	.22***	.19***	.21***	.00024	-.0018	-.019***
	[.18, .26]	[.14, .23]	[.06, .36]	[-.0053, .0058]	[-.0072, .0036]	[-.028, -.0094]
Birth interval (months)	.0047***	.0046***	.0056***	.000079*	.000011	-.00026**
	[.0041, .0053]	[.0039, .0053]	[.0024, .0087]	[-7.9e-06, .00017]	[-.000073, .000095]	[-.00046, -.000062]
Birth order	-.17***	-.19***	-1***	.0086***	7.5e-06	-.018***
	[-.19, -.14]	[-.22, -.16]	[-1.2, -.83]	[.0065, .011]	[-.0019, .0019]	[-.026, -.0091]
Age (months)	-.052***	-.054***	-.08***	.016***	.015***	.016***
	[-.057, -.047]	[-.06, -.049]	[-.093, -.068]	[.015, .016]	[.015, .015]	[.015, .017]
Age (months) squared	.00068***	.00068***	.00064***	-.000053***	-.000054***	-.000058***
	[.00061, .00074]	[.00061, .00076]	[.00049, .0008]	[-.000055, -.000051]	[-.000055, -.000052]	[-.000061, -.000056]
Female	.16***	.16***	.17***	-.012***	-.012***	-.015***
	[.14, .18]	[.14, .18]	[.11, .24]	[-.015, -.0087]	[-.015, -.0085]	[-.02, -.0096]
Mother's age at birth (years)	.063***	.046***		.0022*	-.0015	
	[.049, .077]	[.03, .061]		[-.00021, .0046]	[-.0038, .00082]	
Mother's age at birth (years) squared	-.00072***	-.00048***		-.000038*	.000023	
	[-.00095, -.00049]	[-.00075, -.00021]		[-.000083, 6.5e-06]	[-.00002, .000066]	
Mother's education (years)	.068***	.036***		.019***	.0086***	
	[.065, .072]	[.031, .04]		[.018, .019]	[.0081, .0091]	
Number of siblings	.13***	.16***		-.015***	-.0054***	
	[.11, .15]	[.13, .19]		[-.016, -.013]	[-.0068, -.004]	
Twin	-.6***	-.61***	-.67***	.023***	.0067	.0095
	[-.68, -.52]	[-.7, -.52]	[-.96, -.39]	[.012, .034]	[-.0036, .017]	[-.01, .029]
Constant	-1.6***	-1.8***	-1.7***	.86***	.82***	.83***
	[-1.7, -1.6]	[-1.8, -1.7]	[-1.8, -1.7]	[.85, .86]	[.82, .83]	[.83, .83]
R squared	0.106	0.292	0.705	0.163	0.357	0.658
Observations	89,974	87,752	22,767	230,519	229,270	156,910

Notes: ***p<0.01; **p<0.05; *p<0.1. Linear regression coefficients are shown. Postneonatal mortality rate was centered around percentile 25 (considering the pooled samples for both outcomes) and all covariates were mean-centered (using means for all valid observations in each analysis): therefore, the constant shows the mean outcome when covariates were at their means and postneonatal mortality rate was at percentile 25, and the coefficient of the linear term for postneonatal mortality rate shows the marginal effect at percentile 25. 95% confidence intervals (shown in brackets) and p-values were adjusted for clustering within primary sampling units. Postneonatal mortality rate per 100 person-years within a 25 kilometer radius was linked to the period of infancy.

Figure S5. Marginal effects of postneonatal mortality rate on height-for-age across the distribution of postneonatal mortality rate: Postneonatal mortality rate with distance restricted to 25 kilometers

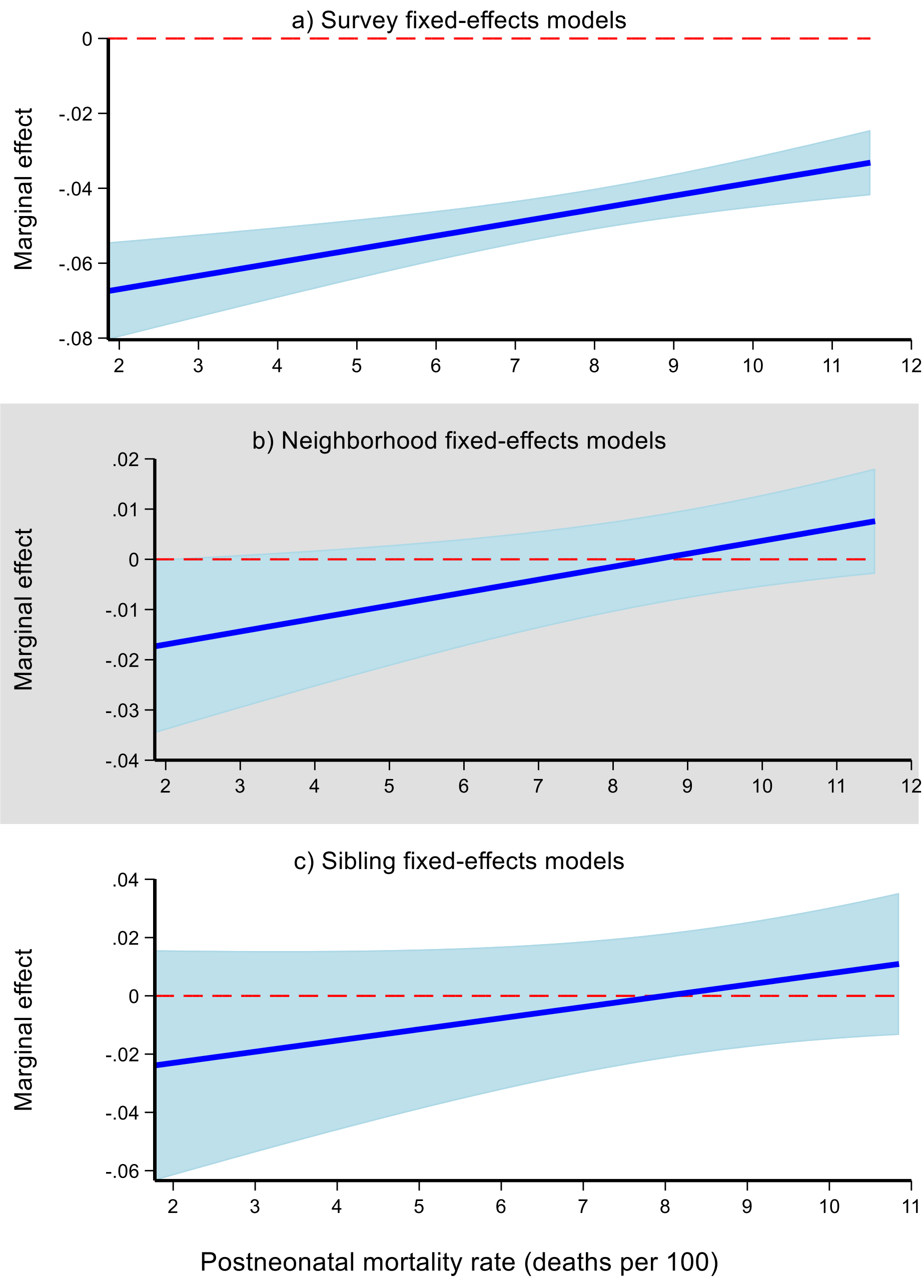

Notes: The values on the y-axis indicate marginal effects: that is, change in the outcome for a single death increase in postneonatal mortality rate per 100 person-years. Since the relationship was nonlinear, the marginal effects vary across the distribution of postneonatal mortality rate. The marginal effects were obtained using the partial derivative of the regression equation with respect to postneonatal mortality rate. The plot was restricted to postneonatal mortality rate between the 5th and 95th percentile (considering the pooled samples for both outcomes). 95% confidence intervals adjusted for clustering within primary sampling units are shown. Postneonatal mortality rate per 100 person-years within a 25 kilometer radius was linked to the period of infancy.

Figure S6. Marginal effects of postneonatal mortality rate on school attendance across the distribution of postneonatal mortality rate: Postneonatal mortality rate with distance restricted to 25 kilometers

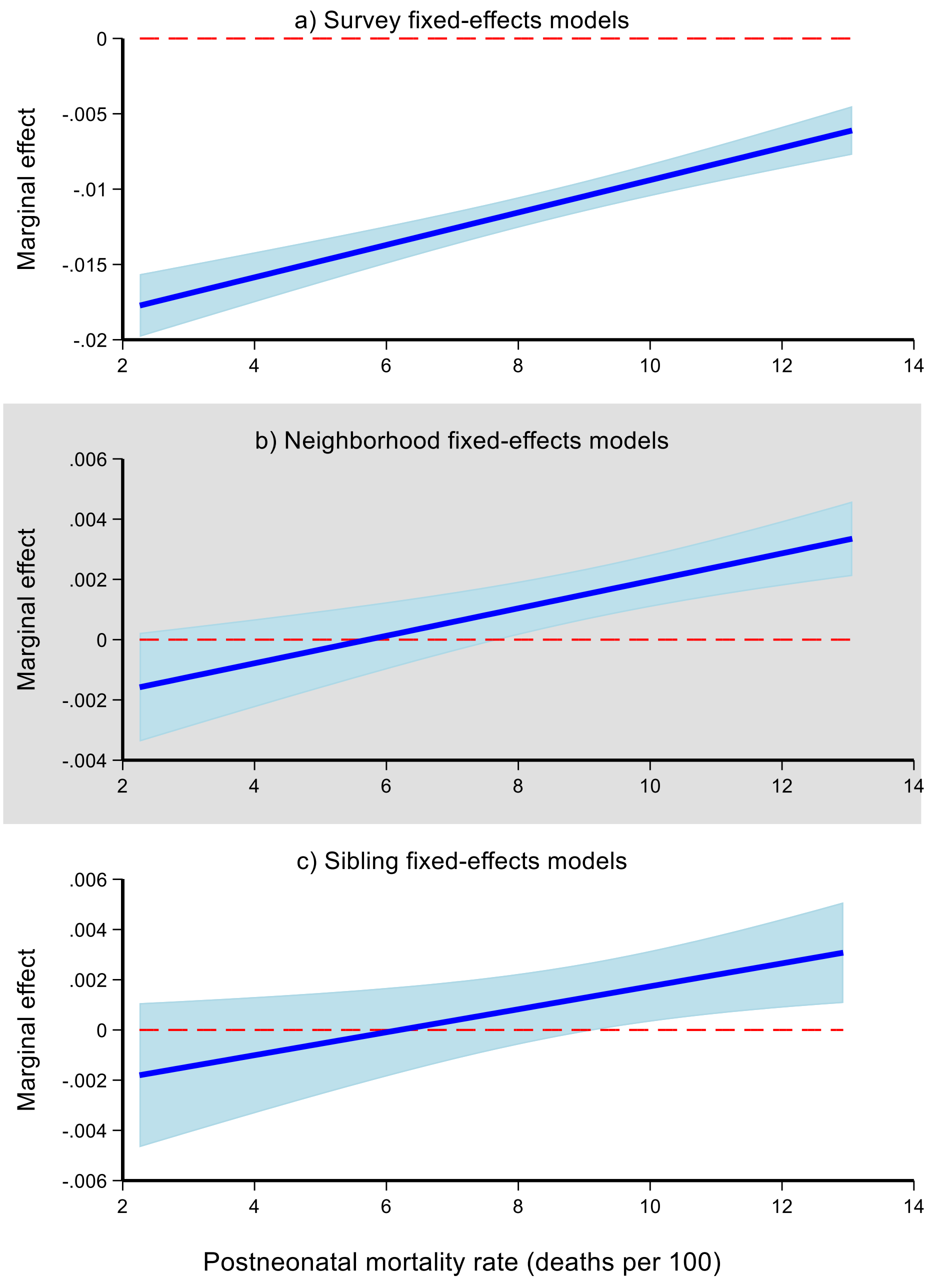

Notes: The values on the y-axis indicate marginal effects: that is, change in the outcome for a single death increase in postneonatal mortality rate per 100 person-years. Since the relationship was nonlinear, the marginal effects vary across the distribution of postneonatal mortality rate. The marginal effects were obtained using the partial derivative of the regression equation with respect to postneonatal mortality rate. The plot was restricted to postneonatal mortality rate between the 5th and 95th percentile (considering the pooled samples for both outcomes). 95% confidence intervals adjusted for clustering within primary sampling units are shown. Postneonatal mortality rate per 100 person-years within a 25 kilometer radius was linked to the period of infancy.

Table S10. Marginal effects of postneonatal mortality rate on height-for-age and school attendance at different percentiles of postneonatal mortality rate: Postneonatal mortality rate with distance restricted to 25 kilometers

Outcome->	Height-for-age z-score			School attendance		
Fixed-effects->	Survey	Neighborhood	Sibling	Survey	Neighborhood	Sibling
Percentiles of postneonatal mortality rate (per 100)						
p1 (1.5)	-.069***	-.019**	-.025	-.018***	-.0019*	-.0021
	[-.083, -.055]	[-.037, -.0004]	[-.067, .016]	[-.021, -.016]	[-.0038, .00006]	[-.0052, .00098]
p5 (2.1)	-.067***	-.017**	-.024	-.018***	-.0016*	-.0018
	[-.08, -.054]	[-.035, -.000046]	[-.063, .016]	[-.02, -.016]	[-.0034, .00023]	[-.0047, .0011]
p10 (2.6)	-.066***	-.016*	-.022	-.017***	-.0014	-.0016
	[-.078, -.054]	[-.033, .00024]	[-.06, .016]	[-.019, -.015]	[-.0031, .00035]	[-.0043, .0011]
p25 (3.7)	-.063***	-.014*	-.019	-.016***	-.00082	-.001
	[-.074, -.052]	[-.029, .001]	[-.054, .015]	[-.018, -.014]	[-.0023, .00066]	[-.0034, .0013]
p50 (5.6)	-.057***	-.0096	-.013	-.014***	.000075	-.00018
	[-.065, -.049]	[-.022, .0027]	[-.041, .016]	[-.015, -.013]	[-.0011, .0012]	[-.002, .0016]
p75 (8.1)	-.048***	-.0035	-.0048	-.011***	.0012***	.00096
	[-.054, -.043]	[-.013, .006]	[-.028, .018]	[-.012, -.01]	[.00039, .0021]	[-.00043, .0023]
p90 (10.8)	-.04***	.0029	.0046	-.0082***	.0025***	.0022***
	[-.046, -.033]	[-.0061, .012]	[-.017, .026]	[-.0094, -.0069]	[.0015, .0034]	[.00065, .0038]
p95 (12.7)	-.033***	.0076	.011	-.0061***	.0034***	.0031***
	[-.042, -.024]	[-.0029, .018]	[-.013, .035]	[-.0077, -.0045]	[.0021, .0046]	[.0011, .0051]
p99 (17.0)	-.018**	.019**	.027	-.0015	.0053***	.005***
	[-.034, -.0021]	[.0014, .036]	[-.012, .066]	[-.0042, .0012]	[.0032, .0074]	[.0017, .0083]

Notes: ***p<0.01; **p<0.05; *p<0.1. Change in outcome for a single increase in postneonatal mortality rate per 100 person-years are shown at different percentiles of postneonatal mortality rate. Since the relationship was nonlinear, the marginal effects vary across the distribution of postneonatal mortality rate. The marginal effects were obtained using the partial derivative of the regression equation with respect to postneonatal mortality rate. The level of postneonatal mortality rate per 100 at each of the percentile is shown in parentheses (considering the pooled samples for both outcomes). 95% confidence intervals adjusted for clustering within primary sampling units are shown in brackets. Postneonatal mortality rate per 100 person-years within a 25 kilometer radius was linked to the period of infancy.

Table S11. Results from linear regression models: Postneonatal mortality rate distance weighted

Outcome->	Height-for-age z-score			School attendance		
Fixed-effects->	Survey	Neighborhood	Sibling	Survey	Neighborhood	Sibling
Independent variables						
Postneonatal mortality rate (per 100)	-.055***	-.01**	-.017	-.019***	-.0014**	-.00048
	[-.062, -.047]	[-.02, -.00065]	[-.039, .0043]	[-.02, -.017]	[-.0025, -.00027]	[-.0021, .0012]
Postneonatal mortality rate (per 100) squared	.0015***	.0006	.0016**	.00067***	.00028***	.00022***
	[.0009, .0022]	[-.00012, .0013]	[.000023, .0032]	[.00055, .00079]	[.00018, .00037]	[.000072, .00036]
Firstborn	.22***	.2***	.24***	.0009	-.00011	-.015***
	[.19, .25]	[.17, .24]	[.14, .33]	[-.0035, .0053]	[-.0042, .0039]	[-.022, -.0085]
Birth interval (months)	.0047***	.0047***	.007***	.00016***	.000061*	-.00024***
	[.0043, .0051]	[.0042, .0051]	[.0051, .009]	[.00009, .00023]	[-1.8e-06, .00012]	[-.00038, -.000097]
Birth order	-.22***	-.23***	-1.1***	.01***	.00075	-.015***
	[-.24, -.2]	[-.25, -.21]	[-1.2, -.98]	[.0088, .012]	[-.00066, .0022]	[-.021, -.0095]
Age (months)	-.055***	-.058***	-.088***	.014***	.014***	.014***
	[-.058, -.052]	[-.061, -.054]	[-.096, -.08]	[.014, .015]	[.013, .014]	[.014, .015]
Age (months) squared	.0007***	.00072***	.00073***	-.000049***	-.000049***	-.000053***
	[.00066, .00074]	[.00068, .00077]	[.00063, .00083]	[-.00005, -.000048]	[-.00005, -.000048]	[-.000055, -.000052]
Female	.16***	.16***	.17***	-.02***	-.022***	-.028***
	[.14, .17]	[.14, .17]	[.13, .21]	[-.023, -.018]	[-.024, -.02]	[-.031, -.024]
Mother's age at birth (years)	.065***	.047***		.0033***	-.0017**	
	[.055, .074]	[.037, .057]		[.0014, .0052]	[-.0034, -.00004]	
Mother's age at birth (years) squared	-.0007***	-.0005***		-.000055***	.000022	
	[-.00085, -.00054]	[-.00067, -.00032]		[-.00009, -.000019]	[-9.7e-06, .000053]	
Mother's education (years)	.074***	.035***		.024***	.0091***	
	[.071, .076]	[.032, .038]		[.023, .024]	[.0087, .0095]	
Number of siblings	.18***	.2***		-.016***	-.0048***	
	[.16, .2]	[.18, .22]		[-.018, -.015]	[-.0059, -.0038]	
Twin	-.58***	-.62***	-.7***	.025***	.0095**	.011
	[-.64, -.53]	[-.68, -.56]	[-.88, -.52]	[.017, .033]	[.0018, .017]	[-.0032, .026]
Constant	-1.7***	-1.8***	-1.7***	.8***	.76***	.76***
	[-1.7, -1.7]	[-1.8, -1.8]	[-1.8, -1.7]	[.79, .8]	[.76, .76]	[.75, .76]
R squared	0.093	0.267	0.699	0.222	0.433	0.706
Observations	199,417	196,543	57,814	442,405	441,159	323,830

Notes: ***p<0.01; **p<0.05; *p<0.1. Linear regression coefficients are shown. Postneonatal mortality rate was centered around percentile 25 (considering the pooled samples for both outcomes) and all covariates were mean-centered (using means for all valid observations in each analysis): therefore, the constant shows the mean outcome when covariates were at their means and postneonatal mortality rate was at percentile 25, and the coefficient of the linear term for postneonatal mortality rate shows the marginal effect at percentile 25. 95% confidence intervals (shown in brackets) and p-values were adjusted for clustering within primary sampling units. Postneonatal mortality rate per 100 person-years within a 50 kilometer radius was linked to the period of infancy.

Figure S7. Marginal effects of postneonatal mortality rate on height-for-age across the distribution of postneonatal mortality rate: Postneonatal mortality rate distance weighted

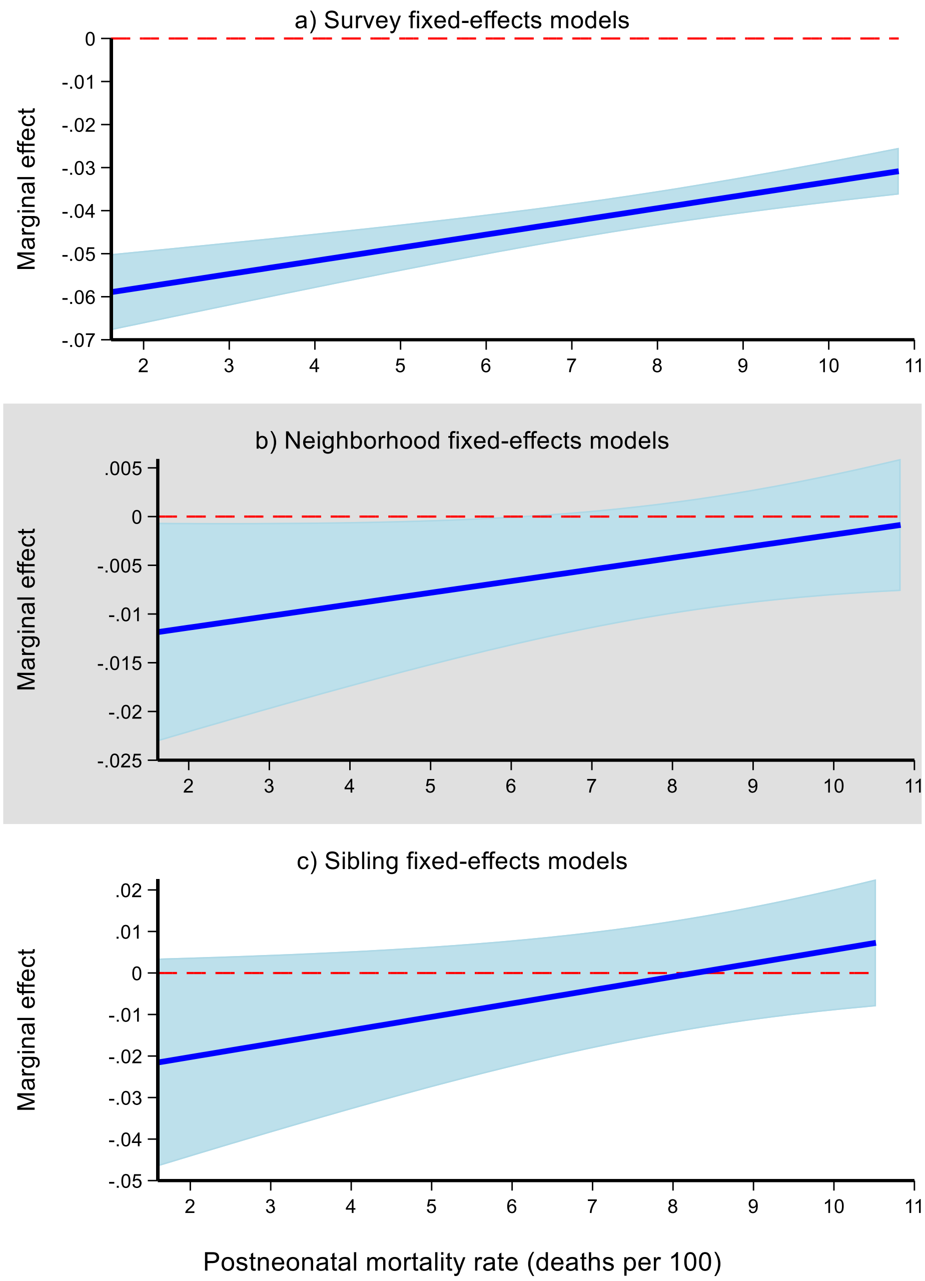

Notes: The values on the y-axis indicate marginal effects: that is, change in the outcome for a single death increase in postneonatal mortality rate per 100 person-years. Since the relationship was nonlinear, the marginal effects vary across the distribution of postneonatal mortality rate. The marginal effects were obtained using the partial derivative of the regression equation with respect to postneonatal mortality rate. The plot was restricted to postneonatal mortality rate between the 5th and 95th percentile (considering the pooled samples for both outcomes). 95% confidence intervals adjusted for clustering within primary sampling units are shown. Postneonatal mortality rate per 100 person-years within a 50 kilometer radius was linked to the period of infancy.

Figure S8. Marginal effects of postneonatal mortality rate on school attendance across the distribution of postneonatal mortality rate: Postneonatal mortality rate distance weighted

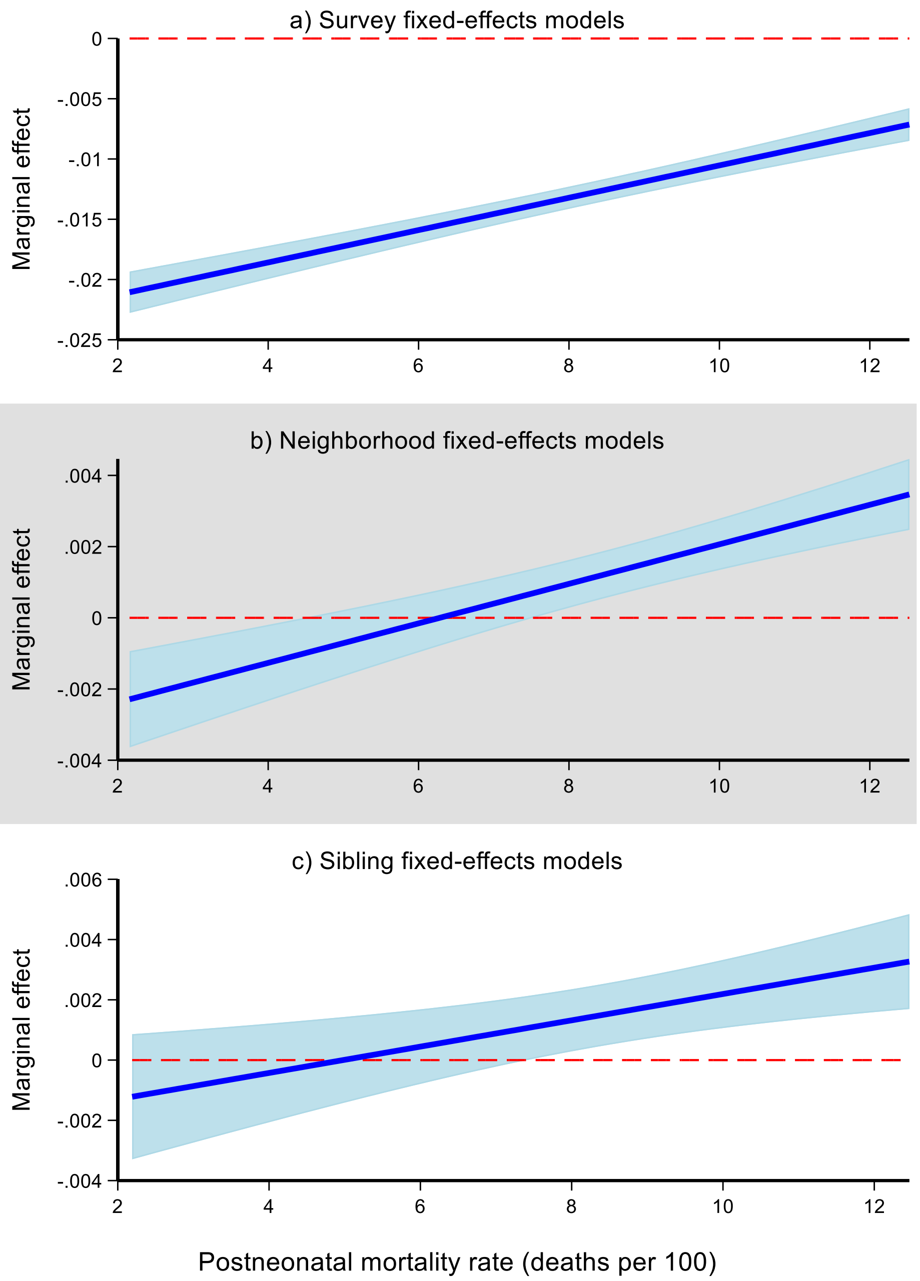

Notes: The values on the y-axis indicate marginal effects: that is, change in the outcome for a single death increase in postneonatal mortality rate per 100 person-years. Since the relationship was nonlinear, the marginal effects vary across the distribution of postneonatal mortality rate. The marginal effects were obtained using the partial derivative of the regression equation with respect to postneonatal mortality rate. The plot was restricted to postneonatal mortality rate between the 5th and 95th percentile (considering the pooled samples for both outcomes). 95% confidence intervals adjusted for clustering within primary sampling units are shown. Postneonatal mortality rate per 100 person-years within a 50 kilometer radius was linked to the period of infancy.

Table S12. Marginal effects of postneonatal mortality rate on height-for-age and school attendance at different percentiles of postneonatal mortality rate: Postneonatal mortality rate distance weighted

Outcome->	Height-for-age z-score			School attendance		
Fixed-effects->	Survey	Neighborhood	Sibling	Survey	Neighborhood	Sibling
Percentiles of postneonatal mortality rate (per 100)						
p1 (1.2)	-.061***	-.012**	-.023*	-.022***	-.0027***	-.0015
	[-.07, -.051]	[-.024, -.00061]	[-.049, .0032]	[-.024, -.02]	[-.0042, -.0012]	[-.0038, .00072]
p5 (1.9)	-.059***	-.012**	-.022*	-.021***	-.0023***	-.0012
	[-.068, -.05]	[-.023, -.00064]	[-.047, .0035]	[-.023, -.019]	[-.0036, -.00094]	[-.0033, .00086]
p10 (2.4)	-.058***	-.011**	-.02*	-.02***	-.002***	-.00099
	[-.066, -.049]	[-.022, -.00065]	[-.044, .0037]	[-.022, -.019]	[-.0033, -.00074]	[-.0029, .00095]
p25 (3.5)	-.055***	-.01**	-.017	-.019***	-.0014**	-.00048
	[-.062, -.047]	[-.02, -.00065]	[-.039, .0043]	[-.02, -.017]	[-.0025, -.00027]	[-.0021, .0012]
p50 (5.4)	-.05***	-.0083**	-.012	-.016***	-.00031	.00034
	[-.056, -.044]	[-.016, -.00047]	[-.03, .0057]	[-.017, -.015]	[-.0012, .00053]	[-.00094, .0016]
p75 (7.7)	-.043***	-.0057*	-.0054	-.013***	.001***	.0014***
	[-.047, -.039]	[-.012, .00045]	[-.02, .009]	[-.014, -.012]	[.00035, .0017]	[.00034, .0024]
p90 (10.3)	-.036***	-.0029	.002	-.0096***	.0025***	.0025***
	[-.04, -.032]	[-.0087, .003]	[-.012, .016]	[-.011, -.0085]	[.0017, .0032]	[.0013, .0037]
p95 (12.1)	-.031***	-.00086	.0073	-.0071***	.0035***	.0033***
	[-.036, -.025]	[-.0076, .0059]	[-.008, .023]	[-.0085, -.0058]	[.0025, .0045]	[.0017, .0048]
p99 (16.2)	-.018***	.0041	.02	-.0016	.0058***	.005***
	[-.028, -.0083]	[-.0071, .015]	[-.0039, .045]	[-.0038, .00058]	[.0041, .0074]	[.0024, .0076]

Notes: ***p<0.01; **p<0.05; *p<0.1. Change in outcome for a single increase in postneonatal mortality rate per 100 person-years are shown at different percentiles of postneonatal mortality rate. Since the relationship was nonlinear, the marginal effects vary across the distribution of postneonatal mortality rate. The marginal effects were obtained using the partial derivative of the regression equation with respect to postneonatal mortality rate. The level of postneonatal mortality rate per 100 at each of the percentile is shown in parentheses (considering the pooled samples for both outcomes). 95% confidence intervals adjusted for clustering within primary sampling units are shown in brackets. Postneonatal mortality rate per 100 person-years within a 50 kilometer radius was linked to the period of infancy.

Table S13. Results from linear regression models: Postneonatal mortality rate restricted to at least 4 deaths and 1200 person-months

Outcome->	Height-for-age z-score			School attendance		
Fixed-effects->	Survey	Neighborhood	Sibling	Survey	Neighborhood	Sibling
Independent variables						
Postneonatal mortality rate (per 100)	-.078***	-.014*	-.025	-.026***	-.0039***	-.0031**
	[-.09, -.067]	[-.029, .0016]	[-.059, .0081]	[-.028, -.024]	[-.0055, -.0022]	[-.0055, -.00062]
Postneonatal mortality rate (per 100) squared	.0026***	.0011*	.0024	.0013***	.00069***	.00064***
	[.0015, .0037]	[-.0001, .0024]	[-.00049, .0053]	[.0011, .0015]	[.00053, .00084]	[.0004, .00089]
Firstborn	.23***	.2***	.25***	.0021	.001	-.022***
	[.19, .26]	[.16, .24]	[.13, .37]	[-.0029, .0072]	[-.0037, .0057]	[-.03, -.013]
Birth interval (months)	.005***	.0048***	.0066***	.00012***	.000059	-.00017**
	[.0044, .0055]	[.0042, .0054]	[.0041, .009]	[.000044, .0002]	[-.000015, .00013]	[-.00034, -1.0e-06]
Birth order	-.21***	-.21***	-1.1***	.0093***	.000091	-.018***
	[-.23, -.19]	[-.24, -.19]	[-1.3, -.96]	[.0074, .011]	[-.0016, .0017]	[-.025, -.011]
Age (months)	-.052***	-.056***	-.099***	.015***	.014***	.015***
	[-.056, -.048]	[-.06, -.052]	[-.11, -.086]	[.015, .015]	[.014, .015]	[.015, .016]
Age (months) squared	.00066***	.0007***	.00074***	-.000051***	-.000051***	-.000057***
	[.00061, .00072]	[.00064, .00076]	[.00062, .00087]	[-.000052, -.000049]	[-.000053, -.00005]	[-.000059, -.000054]
Female	.15***	.16***	.17***	-.018***	-.019***	-.023***
	[.14, .17]	[.14, .18]	[.12, .22]	[-.021, -.015]	[-.022, -.017]	[-.028, -.019]
Mother's age at birth (years)	.063***	.044***	-.061	.0036***	-.0014	-.0082*
	[.051, .074]	[.031, .057]	[-.18, .063]	[.0014, .0058]	[-.0034, .00059]	[-.017, .00044]
Mother's age at birth (years) squared	-.00069***	-.00048***	-.0012	-.000059***	.000018	.00018***
	[-.00089, -.0005]	[-.0007, -.00026]	[-.0027, .00036]	[-.0001, -.000019]	[-.00002, .000055]	[.00009, .00027]
Mother's education (years)	.073***	.035***		.023***	.009***	
	[.069, .076]	[.031, .038]		[.022, .023]	[.0085, .0094]	
Number of siblings	.17***	.19***		-.015***	-.0044***	
	[.15, .19]	[.17, .21]		[-.016, -.014]	[-.0057, -.0032]	
Twin	-.62***	-.64***	-.74***	.026***	.01**	.0092
	[-.68, -.55]	[-.72, -.57]	[-.96, -.52]	[.016, .035]	[.001, .019]	[-.0077, .026]
Constant	-1.7***	-1.8***	-1.8***	.82***	.78***	.78***
	[-1.7, -1.7]	[-1.8, -1.8]	[-1.8, -1.7]	[.82, .83]	[.78, .78]	[.78, .78]
R squared	0.092	0.268	0.704	0.211	0.414	0.695
Observations	129,032	127,003	36,927	313,182	311,949	223,941

Notes: ***p<0.01; **p<0.05; *p<0.1. Linear regression coefficients are shown. Postneonatal mortality rate was centered around percentile 25 (considering the pooled samples for both outcomes) and all covariates were mean-centered (using means for all valid observations in each analysis): therefore, the constant shows the mean outcome when covariates were at their means and postneonatal mortality rate was at percentile 25, and the coefficient of the linear term for postneonatal mortality rate shows the marginal effect at percentile 25. 95% confidence intervals (shown in brackets) and p-values were adjusted for clustering within primary sampling units. Postneonatal mortality rate per 100 person-years within a 50 kilometer radius was linked to the period of infancy.

Figure S9. Marginal effects of postneonatal mortality rate on height-for-age across the distribution of postneonatal mortality rate: Postneonatal mortality rate restricted to at least 4 deaths and 1200 person-months

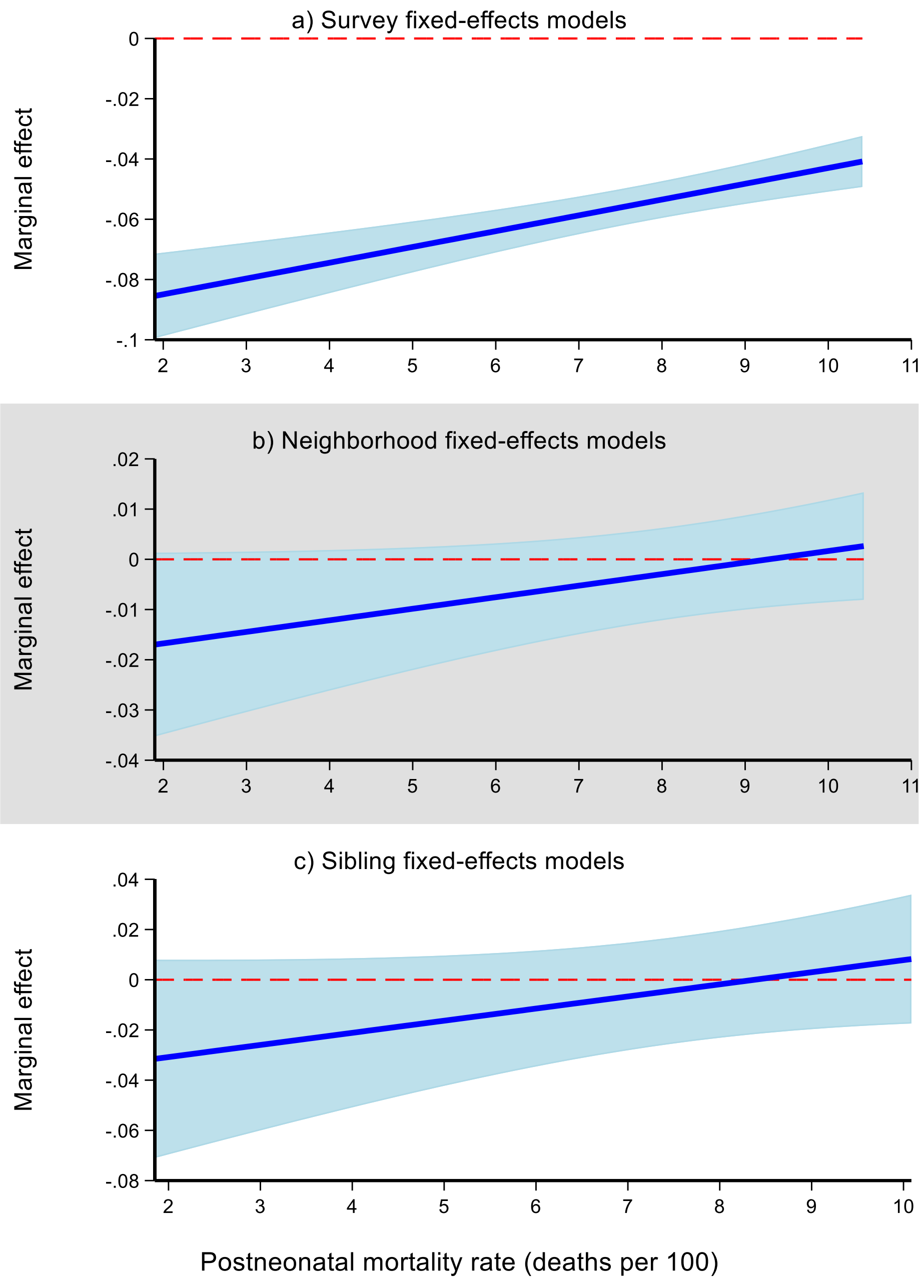

Notes: The values on the y-axis indicate marginal effects: that is, change in the outcome for a single death increase in postneonatal mortality rate per 100 person-years. Since the relationship was nonlinear, the marginal effects vary across the distribution of postneonatal mortality rate. The marginal effects were obtained using the partial derivative of the regression equation with respect to postneonatal mortality rate. The plot was restricted to postneonatal mortality rate between the 5th and 95th percentile (considering the pooled samples for both outcomes). 95% confidence intervals adjusted for clustering within primary sampling units are shown. Postneonatal mortality rate per 100 person-years within a 50 kilometer radius was linked to the period of infancy.

Figure S10. Marginal effects of postneonatal mortality rate on school attendance across the distribution of postneonatal mortality rate: Postneonatal mortality rate restricted to at least 4 deaths and 1200 person-months

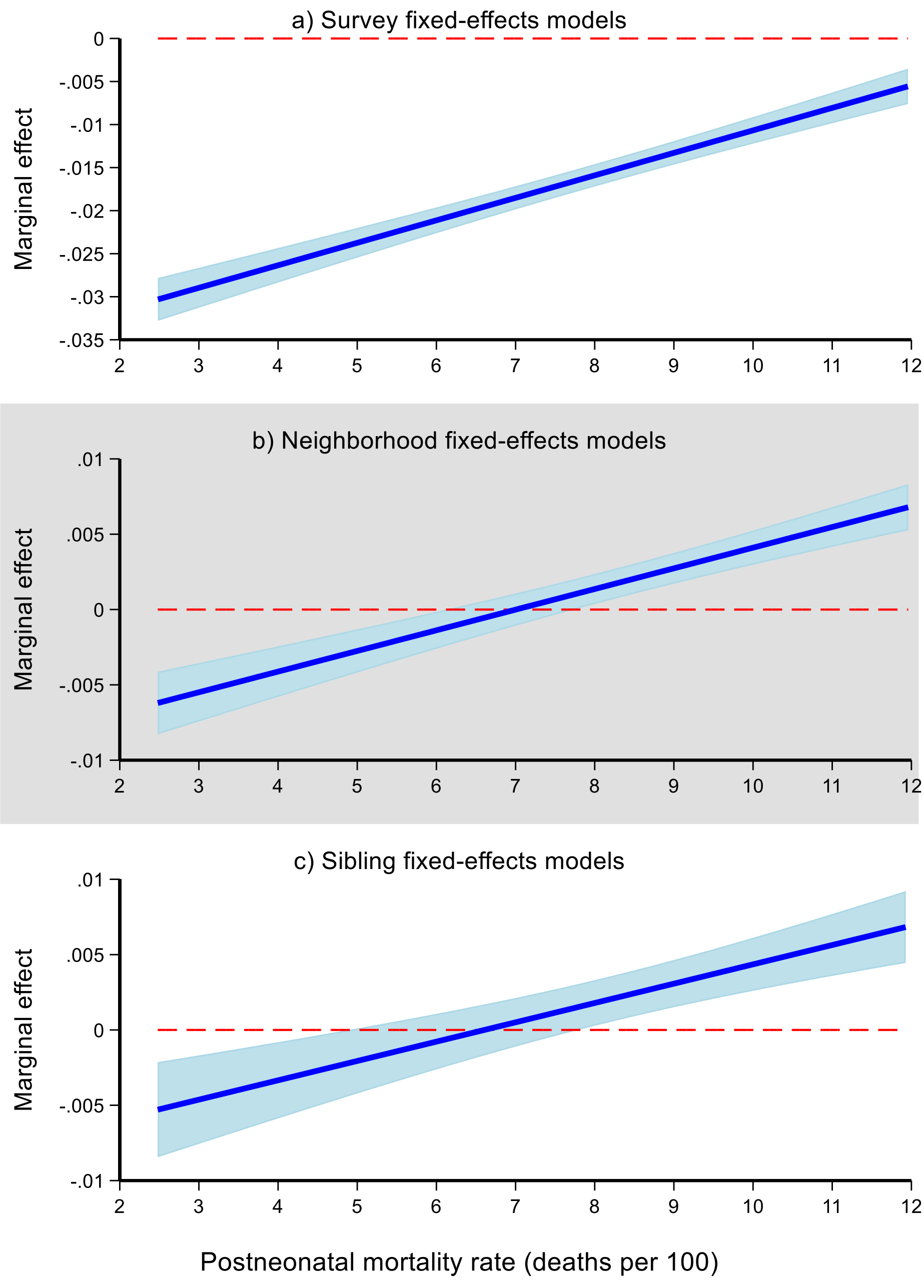

Notes: The values on the y-axis indicate marginal effects: that is, change in the outcome for a single death increase in postneonatal mortality rate per 100 person-years. Since the relationship was nonlinear, the marginal effects vary across the distribution of postneonatal mortality rate. The marginal effects were obtained using the partial derivative of the regression equation with respect to postneonatal mortality rate. The plot was restricted to postneonatal mortality rate between the 5th and 95th percentile (considering the pooled samples for both outcomes). 95% confidence intervals adjusted for clustering within primary sampling units are shown. Postneonatal mortality rate per 100 person-years within a 50 kilometer radius was linked to the period of infancy.

Table S14. Marginal effects of postneonatal mortality rate on height-for-age and school attendance at different percentiles of postneonatal mortality rate: Postneonatal mortality rate restricted to at least 4 deaths and 1200 person-months

Outcome->	Height-for-age z-score			School attendance		
Fixed-effects->	Survey	Neighborhood	Sibling	Survey	Neighborhood	Sibling
Percentiles of postneonatal mortality rate (per 100)						
p1 (1.5)	-.088***	-.018*	-.034	-.032***	-.0073***	-.0063***
	[-.1, -.073]	[-.038, .0013]	[-.076, .008]	[-.035, -.03]	[-.0096, -.005]	[-.0098, -.0028]
p5 (2.3)	-.085***	-.017*	-.032	-.03***	-.0062***	-.0053***
	[-.099, -.071]	[-.035, .0013]	[-.071, .008]	[-.033, -.028]	[-.0083, -.0041]	[-.0084, -.0021]
p10 (2.8)	-.083***	-.016*	-.03	-.029***	-.0055***	-.0046***
	[-.096, -.07]	[-.033, .0014]	[-.067, .008]	[-.031, -.027]	[-.0074, -.0036]	[-.0075, -.0017]
p25 (3.9)	-.078***	-.014*	-.025	-.026***	-.0039***	-.0031**
	[-.09, -.067]	[-.029, .0016]	[-.059, .0081]	[-.028, -.024]	[-.0055, -.0022]	[-.0055, -.00062]
p50 (5.7)	-.069***	-.0099	-.017	-.021***	-.0013**	-.00072
	[-.078, -.061]	[-.022, .0023]	[-.044, .0093]	[-.022, -.02]	[-.0025, -.0001]	[-.0025, .0011]
p75 (7.9)	-.058***	-.0051	-.0075	-.015***	.0017***	.002***
	[-.065, -.052]	[-.015, .0045]	[-.029, .014]	[-.017, -.014]	[.00067, .0027]	[.00052, .0035]
p90 (10.1)	-.048***	-.00059	.0017	-.0096***	.0047***	.0049***
	[-.055, -.041]	[-.01, .0088]	[-.02, .024]	[-.011, -.008]	[.0035, .0059]	[.003, .0067]
p95 (11.6)	-.041***	.0026	.0082	-.0056***	.0068***	.0068***
	[-.049, -.032]	[-.0081, .013]	[-.017, .034]	[-.0076, -.0035]	[.0053, .0083]	[.0045, .0092]
p99 (15.0)	-.023***	.011	.024	.0031*	.011***	.011***
	[-.038, -.0082]	[-.0063, .027]	[-.016, .064]	[-.000036, .0063]	[.0089, .014]	[.0073, .015]

Notes: ***p<0.01; **p<0.05; *p<0.1. Change in outcome for a single increase in postneonatal mortality rate per 100 person-years are shown at different percentiles of postneonatal mortality rate. Since the relationship was nonlinear, the marginal effects vary across the distribution of postneonatal mortality rate. The marginal effects were obtained using the partial derivative of the regression equation with respect to postneonatal mortality rate. The level of postneonatal mortality rate per 100 at each of the percentile is shown in parentheses (considering the pooled samples for both outcomes). 95% confidence intervals adjusted for clustering within primary sampling units are shown in brackets. Postneonatal mortality rate per 100 person-years within a 50 kilometer radius was linked to the period of infancy.

Table S15. Results from linear regression models: Infant mortality rate (ie, including neonatal deaths) as a measure of adversity

Outcome->	Height-for-age z-score			School attendance		
Fixed-effects->	Survey	Neighborhood	Sibling	Survey	Neighborhood	Sibling
Independent variables						
Postneonatal mortality rate (per 100)	-.034***	-.0081**	-.006	-.011***	-.0025***	-.002***
	[-.039, -.029]	[-.015, -.0017]	[-.021, .009]	[-.012, -.01]	[-.0033, -.0018]	[-.0031, -.00088]
Postneonatal mortality rate (per 100) squared	.00046**	.00049**	.00077	.0002***	.00023***	.0002***
	[.000095, .00083]	[.000043, .00094]	[-.00028, .0018]	[.00012, .00028]	[.00018, .00029]	[.00012, .00029]
Firstborn	.22***	.2***	.21***	.0031	.001	-.014***
	[.2, .24]	[.18, .23]	[.13, .29]	[-.00093, .0071]	[-.0027, .0047]	[-.02, -.0078]
Birth interval (months)	.0046***	.0045***	.0066***	.00019***	.000084***	-.0002***
	[.0043, .005]	[.0041, .0049]	[.005, .0082]	[.00013, .00025]	[.000028, .00014]	[-.00032, -.000081]
Birth order	-.21***	-.22***	-1.1***	.011***	.00034	-.015***
	[-.23, -.2]	[-.23, -.2]	[-1.2, -.99]	[.0093, .012]	[-.00095, .0016]	[-.02, -.0094]
Age (months)	-.055***	-.058***	-.088***	.014***	.014***	.014***
	[-.058, -.053]	[-.061, -.055]	[-.095, -.082]	[.014, .014]	[.013, .014]	[.014, .015]
Age (months) squared	.00071***	.00073***	.00075***	-.000048***	-.000048***	-.000052***
	[.00068, .00075]	[.0007, .00077]	[.00067, .00083]	[-.000049, -.000047]	[-.000049, -.000047]	[-.000054, -.00005]
Female	.16***	.16***	.16***	-.02***	-.021***	-.026***
	[.15, .17]	[.15, .17]	[.13, .2]	[-.022, -.017]	[-.023, -.019]	[-.03, -.023]
Mother's age at birth (years)	.065***	.046***		.0036***	-.0014*	
	[.057, .073]	[.037, .055]		[.0019, .0054]	[-.003, .00011]	
Mother's age at birth (years) squared	-.00071***	-.00049***		-.000059***	.000018	
	[-.00084, -.00057]	[-.00064, -.00035]		[-.000092, -.000027]	[-.000011, .000046]	
Mother's education (years)	.072***	.033***		.024***	.0089***	
	[.07, .074]	[.031, .036]		[.023, .025]	[.0086, .0093]	
Number of siblings	.17***	.2***		-.017***	-.0046***	
	[.16, .18]	[.18, .21]		[-.018, -.016]	[-.0055, -.0036]	
Twin	-.57***	-.61***	-.77***	.023***	.0089**	.012*
	[-.62, -.53]	[-.66, -.57]	[-.91, -.62]	[.016, .031]	[.0019, .016]	[-.00088, .024]
Constant	-1.7***	-1.7***	-1.7***	.79***	.76***	.76***
	[-1.7, -1.6]	[-1.7, -1.7]	[-1.7, -1.7]	[.79, .79]	[.76, .76]	[.76, .76]
R squared	0.095	0.265	0.700	0.221	0.433	0.707
Observations	272,877	270,570	84,689	535,978	535,283	405,824

Notes: ***p<0.01; **p<0.05; *p<0.1. Linear regression coefficients are shown. Infant mortality rate was centered around percentile 25 (considering the pooled samples for both outcomes) and all covariates were mean-centered (using means for all valid observations in each analysis): therefore, the constant shows the mean outcome when covariates were at their means and infant mortality rate was at percentile 25, and the coefficient of the linear term for infant mortality rate shows the marginal effect at percentile 25. 95% confidence intervals (shown in brackets) and p-values were adjusted for clustering within primary sampling units. Infant mortality rate per 100 person-years within a 50 kilometer radius was linked to the period of infancy.

Figure S11. Marginal effects of postneonatal mortality rate on height-for-age across the distribution of postneonatal mortality rate: Infant mortality rate (ie, including neonatal deaths) as a measure of adversity

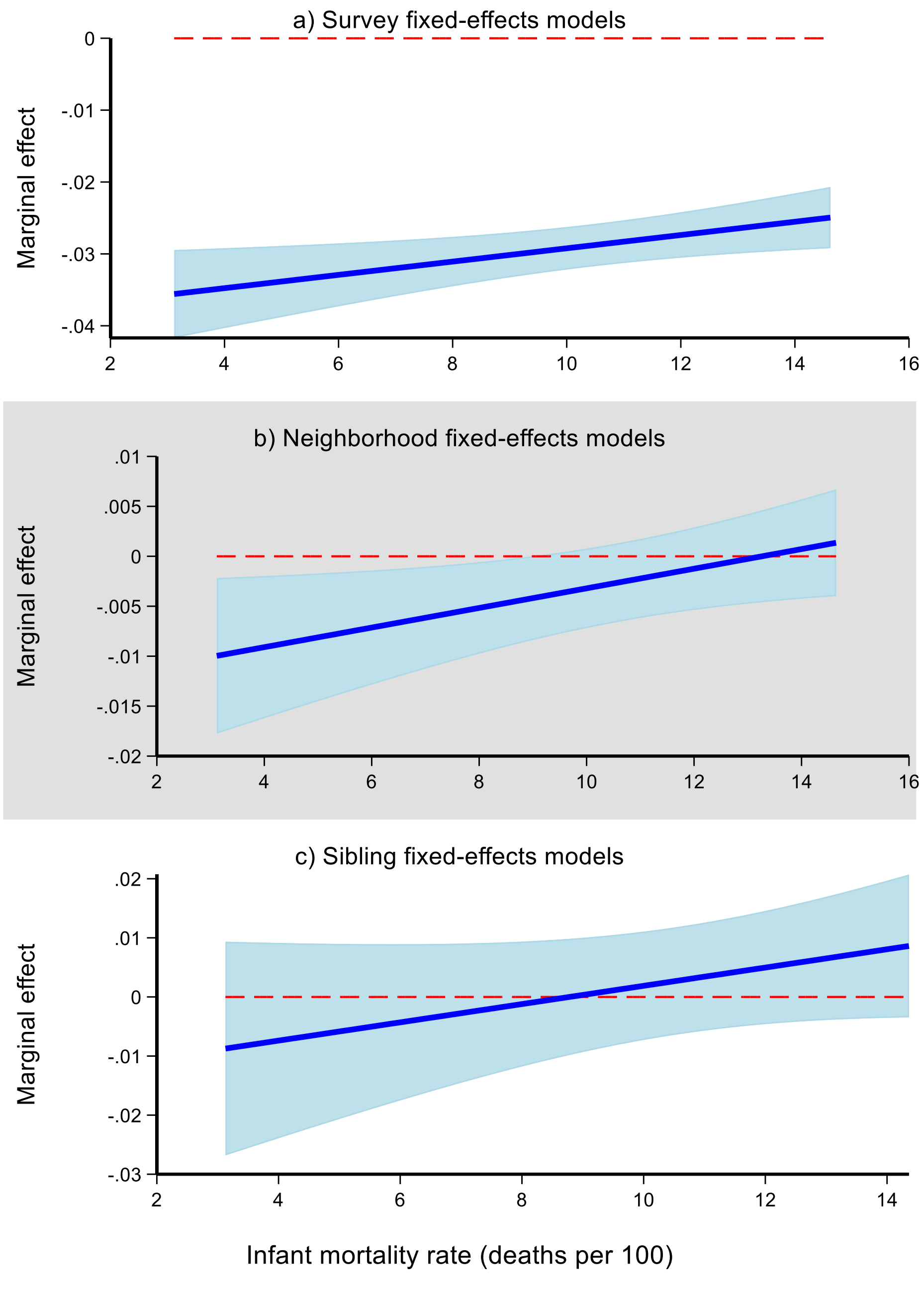

Notes: The values on the y-axis indicate marginal effects: that is, change in the outcome for a single death increase in infant mortality rate per 100 person-years. Since the relationship was nonlinear, the marginal effects vary across the distribution of infant mortality rate. The marginal effects were obtained using the partial derivative of the regression equation with respect to postneonatal mortality rate. The plot was restricted to infant mortality rate between the 5th and 95th percentile (considering the pooled samples for both outcomes). 95% confidence intervals adjusted for clustering within primary sampling units are shown. Infant mortality rate per 100 person-years within a 50 kilometer radius was linked to the period of infancy.

Figure S12. Marginal effects of postneonatal mortality rate on school attendance across the distribution of postneonatal mortality rate: Infant mortality rate (ie, including neonatal deaths) as a measure of adversity

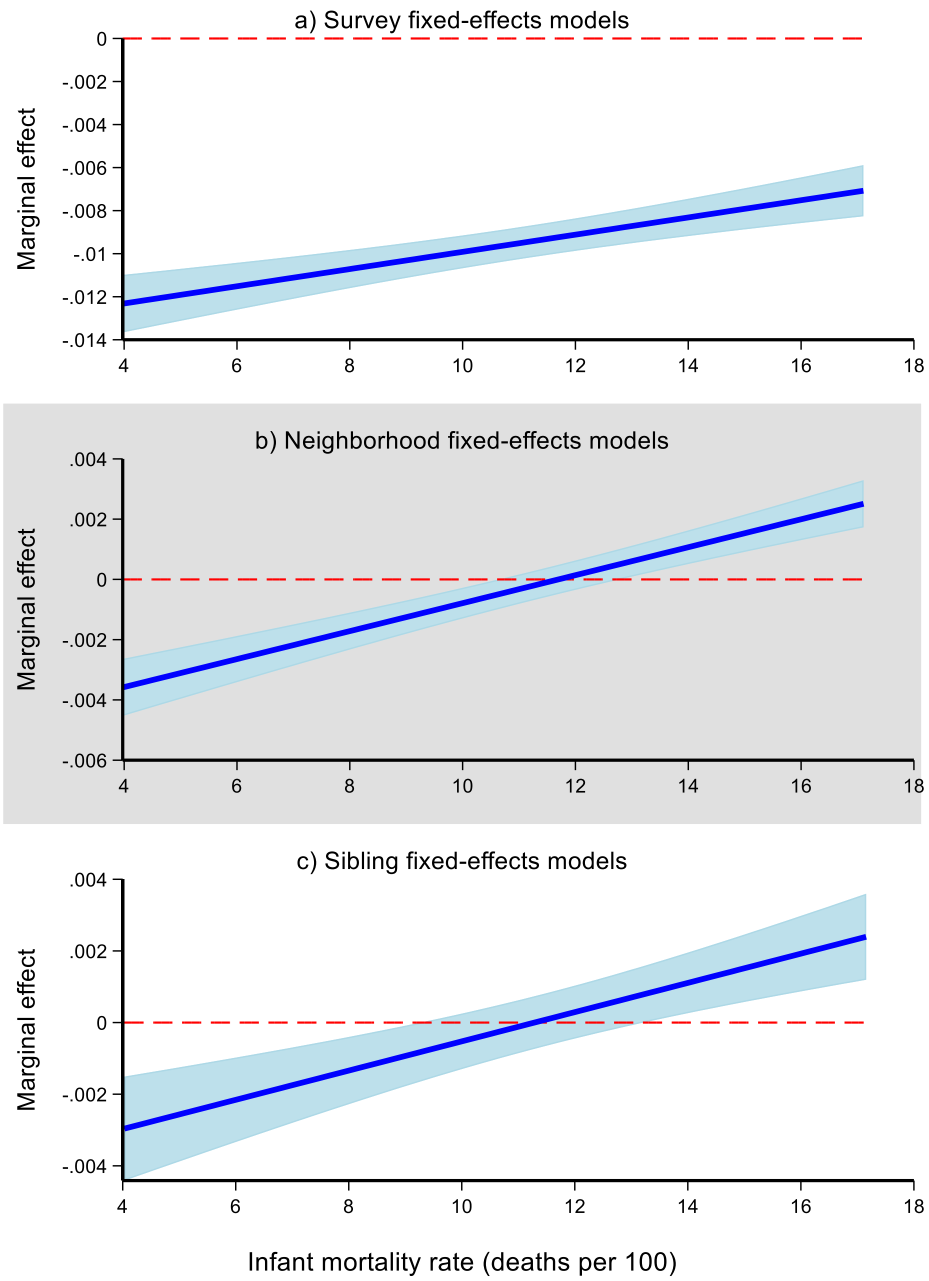

Notes: The values on the y-axis indicate marginal effects: that is, change in the outcome for a single death increase in infant mortality rate per 100 person-years. Since the relationship was nonlinear, the marginal effects vary across the distribution of infant mortality rate. The marginal effects were obtained using the partial derivative of the regression equation with respect to postneonatal mortality rate. The plot was restricted to infant mortality rate between the 5th and 95th percentile (considering the pooled samples for both outcomes). 95% confidence intervals adjusted for clustering within primary sampling units are shown. Infant mortality rate per 100 person-years within a 50 kilometer radius was linked to the period of infancy.

Table S16. Marginal effects of postneonatal mortality rate on height-for-age and school attendance at different percentiles of postneonatal mortality rate: Infant mortality rate (ie, including neonatal deaths) as a measure of adversity

Outcome->	Height-for-age z-score			School attendance		
Fixed-effects->	Survey	Neighborhood	Sibling	Survey	Neighborhood	Sibling
Percentiles of infant mortality rate (per 100)						
p1 (2.6)	-.036***	-.011**	-.01	-.013***	-.0041***	-.0034***
	[-.043, -.03]	[-.019, -.0023]	[-.03, .0096]	[-.014, -.011]	[-.0051, -.003]	[-.005, -.0018]
p5 (3.6)	-.036***	-.01**	-.0087	-.012***	-.0036***	-.003***
	[-.042, -.029]	[-.018, -.0022]	[-.027, .0094]	[-.014, -.011]	[-.0045, -.0026]	[-.0044, -.0015]
p10 (4.3)	-.035***	-.0094**	-.0079	-.012***	-.0032***	-.0027***
	[-.041, -.029]	[-.017, -.002]	[-.025, .0092]	[-.013, -.011]	[-.0041, -.0024]	[-.004, -.0013]
p25 (5.8)	-.034***	-.0081**	-.006	-.011***	-.0025***	-.002***
	[-.039, -.029]	[-.015, -.0017]	[-.021, .009]	[-.012, -.01]	[-.0033, -.0018]	[-.0031, -.00088]
p50 (8.1)	-.032***	-.0061**	-.0028	-.01***	-.0014***	-.0011**
	[-.036, -.028]	[-.011, -.001]	[-.015, .009]	[-.011, -.0096]	[-.002, -.00086]	[-.0019, -.00018]
p75 (11.0)	-.03***	-.0035*	.0012	-.0093***	-.000028	.00018
	[-.032, -.027]	[-.0075, .00052]	[-.0081, .011]	[-.01, -.0085]	[-.00051, .00046]	[-.00056, .00091]
p90 (14.2)	-.027***	-.00066	.0056	-.008***	.0015***	.0015***
	[-.03, -.024]	[-.005, .0037]	[-.0043, .015]	[-.0089, -.007]	[.00087, .0021]	[.00056, .0024]
p95 (16.5)	-.025***	.0014	.0086	-.0071***	.0025***	.0024***
	[-.029, -.021]	[-.004, .0067]	[-.0035, .021]	[-.0083, -.0059]	[.0017, .0033]	[.0012, .0036]
p99 (21.2)	-.021***	.0059	.016	-.0052***	.0047***	.0043***
	[-.028, -.013]	[-.0028, .015]	[-.0041, .035]	[-.007, -.0034]	[.0035, .0059]	[.0024, .0062]

Notes: ***p<0.01; **p<0.05; *p<0.1. Change in outcome for a single increase in infant mortality rate per 100 person-years are shown at different percentiles of infant mortality rate. Since the relationship was nonlinear, the marginal effects vary across the distribution of infant mortality rate. The marginal effects were obtained using the partial derivative of the regression equation with respect to infant mortality rate. The level of infant mortality rate per 100 at each of the percentile is shown in parentheses (considering the pooled samples for both outcomes). 95% confidence intervals adjusted for clustering within primary sampling units are shown in brackets. Infant mortality rate per 100 person-years within a 50 kilometer radius was linked to the period of infancy.

Table S17. Results from linear regression models: Postneonatal mortality rate cubed added to regression models

Outcome->	Height-for-age z-score			School attendance		
Fixed-effects->	Survey	Neighborhood	Sibling	Survey	Neighborhood	Sibling
Independent variables						
Postneonatal mortality rate (per 100)	-.064***	-.016**	-.028*	-.023***	-.0038***	-.0036***
	[-.074, -.054]	[-.03, -.0025]	[-.062, .0049]	[-.025, -.022]	[-.0052, -.0023]	[-.0057, -.0015]
Postneonatal mortality rate (per 100) squared	.0028***	.0013	.0026	.0015***	.00089***	.0011***
	[.00096, .0047]	[-.0009, .0035]	[-.0026, .0079]	[.0012, .0019]	[.00063, .0011]	[.00066, .0015]
Postneonatal mortality rate (per 100) cubed	-.000046	-.000011	-.000024	-.000039***	-.000028***	-.00004***
	[-.00014, .000043]	[-.0001, .000082]	[-.00023, .00018]	[-.000054, -.000023]	[-.000041, -.000015]	[-.00006, -.00002]
Firstborn	.22***	.2***	.24***	.00086	-.00009	-.015***
	[.19, .25]	[.17, .24]	[.14, .33]	[-.0035, .0052]	[-.0041, .004]	[-.022, -.0083]
Birth interval (months)	.0047***	.0047***	.007***	.00016***	.00006*	-.00024***
	[.0043, .0051]	[.0042, .0051]	[.005, .009]	[.00009, .00023]	[-2.4e-06, .00012]	[-.00038, -.000098]
Birth order	-.22***	-.23***	-1.1***	.01***	.00078	-.015***
	[-.24, -.2]	[-.25, -.21]	[-1.2, -.98]	[.0088, .012]	[-.00063, .0022]	[-.021, -.0093]
Age (months)	-.055***	-.058***	-.088***	.015***	.014***	.015***
	[-.058, -.052]	[-.061, -.054]	[-.095, -.08]	[.014, .015]	[.013, .014]	[.014, .015]
Age (months) squared	.0007***	.00072***	.00073***	-.000049***	-.000049***	-.000053***
	[.00066, .00074]	[.00068, .00077]	[.00063, .00083]	[-.00005, -.000048]	[-.000051, -.000048]	[-.000055, -.000052]
Female	.16***	.16***	.17***	-.02***	-.022***	-.028***
	[.14, .17]	[.14, .17]	[.13, .21]	[-.023, -.018]	[-.024, -.02]	[-.031, -.024]
Mother's age at birth (years)	.065***	.047***		.0032***	-.0017**	
	[.055, .074]	[.037, .057]		[.0013, .0051]	[-.0034, -9.9e-06]	
Mother's age at birth (years) squared	-.0007***	-.0005***		-.000054***	.000021	
	[-.00085, -.00054]	[-.00067, -.00032]		[-.000089, -.000018]	[-.00001, .000053]	
Mother's education (years)	.073***	.035***		.024***	.0091***	
	[.071, .076]	[.032, .038]		[.023, .024]	[.0087, .0095]	
Number of siblings	.18***	.2***		-.016***	-.0049***	
	[.16, .2]	[.18, .22]		[-.018, -.015]	[-.0059, -.0038]	
Twin	-.58***	-.62***	-.7***	.025***	.0095**	.011
	[-.64, -.53]	[-.68, -.56]	[-.88, -.53]	[.017, .033]	[.0018, .017]	[-.0031, .026]
Constant	-1.7***	-1.8***	-1.7***	.8***	.76***	.76***
	[-1.7, -1.7]	[-1.8, -1.8]	[-1.8, -1.7]	[.79, .8]	[.76, .76]	[.75, .76]
R squared	0.093	0.267	0.699	0.222	0.433	0.706
Observations	199,417	196,543	57,814	442,405	441,159	323,830

Notes: ***p<0.01; **p<0.05; *p<0.1. Linear regression coefficients are shown. Postneonatal mortality rate was centered around percentile 25 (considering the pooled samples for both outcomes) and all covariates were mean-centered (using means for all valid observations in each analysis): therefore, the constant shows the mean outcome when covariates were at their means and postneonatal mortality rate was at percentile 25, and the coefficient of the linear term for postneonatal mortality rate shows the marginal effect at percentile 25. 95% confidence intervals (shown in brackets) and p-values were adjusted for clustering within primary sampling units. Postneonatal mortality rate per 100 person-years within a 50 kilometer radius was linked to the period of infancy.

Figure S13. Marginal effects of postneonatal mortality rate on height-for-age across the distribution of postneonatal mortality rate: Postneonatal mortality rate cubed added to regression models

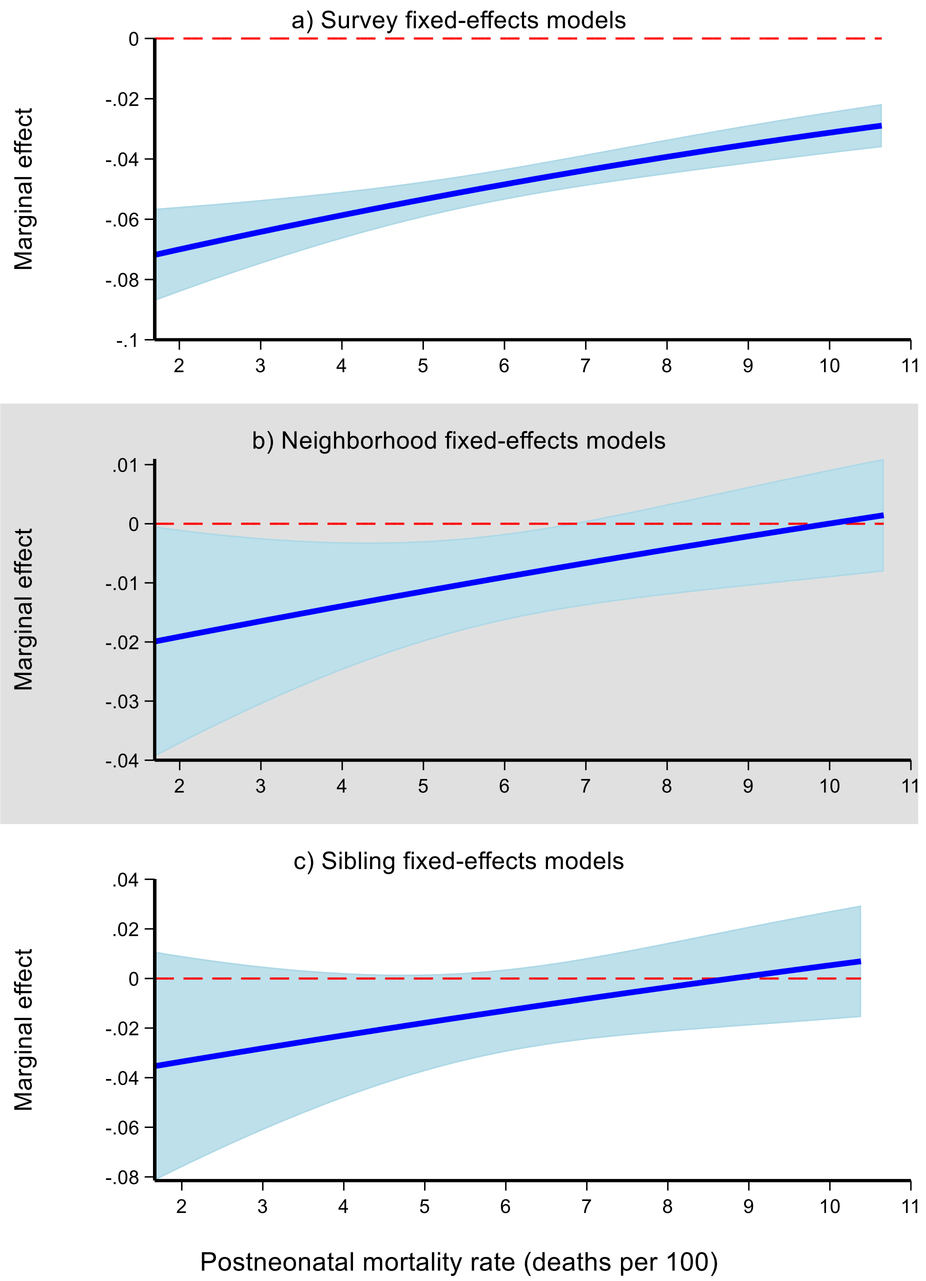

Notes: The values on the y-axis indicate marginal effects: that is, change in the outcome for a single death increase in postneonatal mortality rate per 100 person-years. Since the relationship was nonlinear, the marginal effects vary across the distribution of postneonatal mortality rate. The marginal effects were obtained using the partial derivative of the regression equation with respect to postneonatal mortality rate. The plot was restricted to postneonatal mortality rate between the 5th and 95th percentile (considering the pooled samples for both outcomes). 95% confidence intervals adjusted for clustering within primary sampling units are shown. Postneonatal mortality rate per 100 person-years within a 50 kilometer radius was linked to the period of infancy.

Figure S14. Marginal effects of postneonatal mortality rate on school attendance across the distribution of postneonatal mortality rate: Postneonatal mortality rate cubed added to regression models

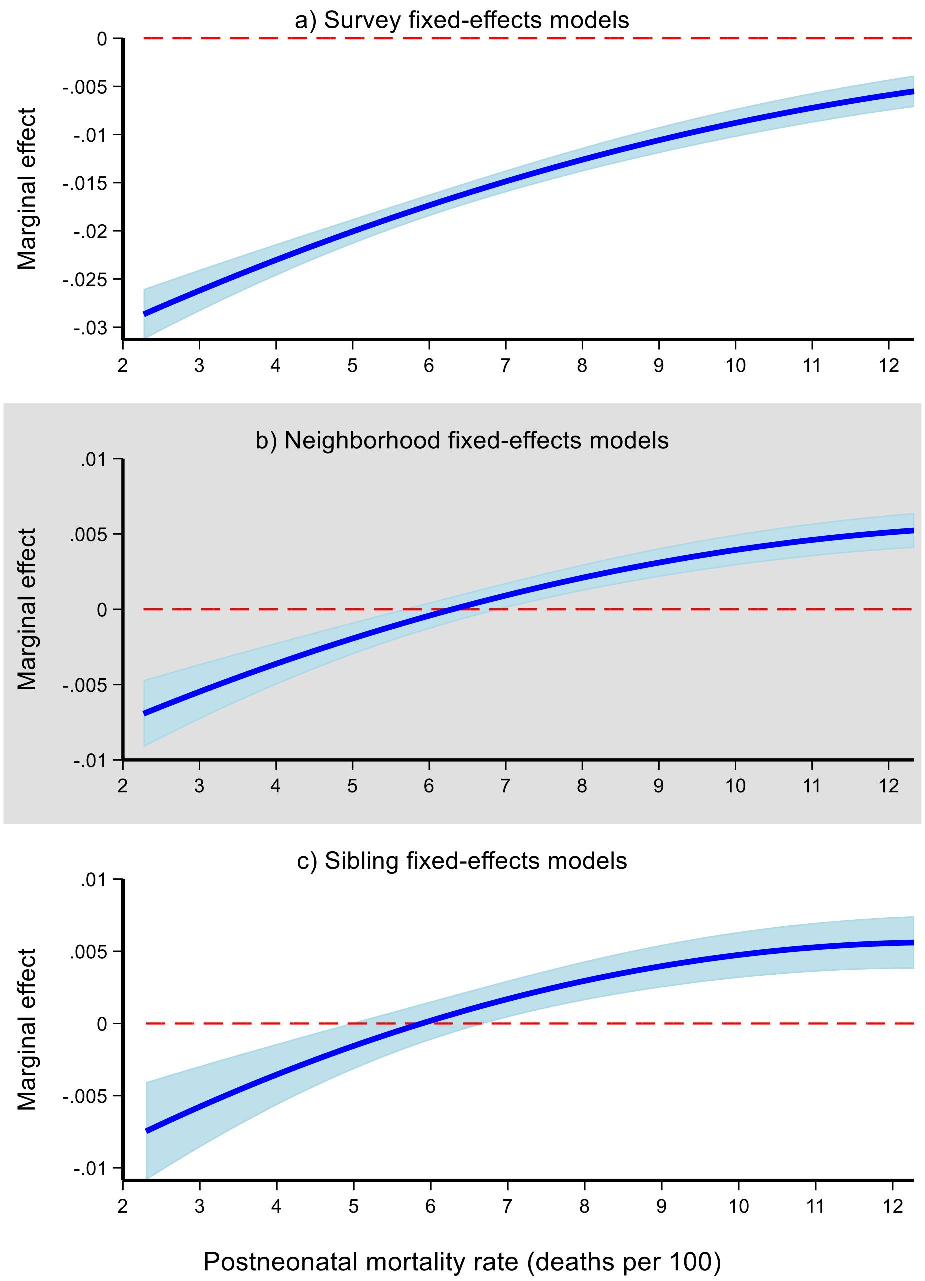

Notes: The values on the y-axis indicate marginal effects: that is, change in the outcome for a single death increase in postneonatal mortality rate per 100 person-years. Since the relationship was nonlinear, the marginal effects vary across the distribution of postneonatal mortality rate. The marginal effects were obtained using the partial derivative of the regression equation with respect to postneonatal mortality rate. The plot was restricted to postneonatal mortality rate between the 5th and 95th percentile (considering the pooled samples for both outcomes). 95% confidence intervals adjusted for clustering within primary sampling units are shown. Postneonatal mortality rate per 100 person-years within a 50 kilometer radius was linked to the period of infancy.

Table S18. Marginal effects of postneonatal mortality rate on height-for-age and school attendance at different percentiles of postneonatal mortality rate: Postneonatal mortality rate cubed added to regression models

Outcome->	Height-for-age z-score			School attendance		
Fixed-effects->	Survey	Neighborhood	Sibling	Survey	Neighborhood	Sibling
Percentiles of postneonatal mortality rate (per 100)						
p1 (1.4)	-.075***	-.021*	-.038	-.031***	-.0085***	-.0094***
	[-.092, -.057]	[-.043, .00061]	[-.089, .013]	[-.034, -.028]	[-.011, -.0058]	[-.014, -.0053]
p5 (2.0)	-.072***	-.02**	-.035	-.029***	-.0069***	-.0075***
	[-.087, -.057]	[-.039, -.00044]	[-.081, .011]	[-.031, -.026]	[-.0092, -.0047]	[-.011, -.0041]
p10 (2.5)	-.069***	-.019**	-.033	-.027***	-.0059***	-.0062***
	[-.083, -.056]	[-.036, -.0012]	[-.075, .0088]	[-.029, -.025]	[-.0079, -.004]	[-.0092, -.0033]
p25 (3.6)	-.064***	-.016**	-.028*	-.023***	-.0038***	-.0036***
	[-.074, -.054]	[-.03, -.0025]	[-.062, .0049]	[-.025, -.022]	[-.0052, -.0023]	[-.0057, -.0015]
p50 (5.4)	-.055***	-.012***	-.02*	-.018***	-.00071	-.000065
	[-.062, -.049]	[-.021, -.0031]	[-.042, .0016]	[-.019, -.017]	[-.0016, .00019]	[-.0014, .0013]
p75 (7.8)	-.044***	-.007*	-.0098	-.012***	.0023***	.0031***
	[-.05, -.039]	[-.014, .00011]	[-.026, .0064]	[-.014, -.011]	[.0014, .0031]	[.0018, .0045]
p90 (10.2)	-.035***	-.002	.000032	-.0078***	.0044***	.0051***
	[-.041, -.029]	[-.01, .0064]	[-.019, .02]	[-.0093, -.0063]	[.0033, .0054]	[.0034, .0067]
p95 (11.9)	-.029***	.0014	.007	-.0055***	.0052***	.0056***
	[-.036, -.022]	[-.0081, .011]	[-.016, .029]	[-.0071, -.0039]	[.0041, .0064]	[.0038, .0074]
p99 (15.8)	-.017***	.009	.022	-.0028**	.0053***	.0041***
	[-.028, -.0066]	[-.0035, .022]	[-.0047, .049]	[-.0052, -.00044]	[.0034, .0072]	[.0012, .0071]

Notes: ***p<0.01; **p<0.05; *p<0.1. Change in outcome for a single increase in postneonatal mortality rate per 100 person-years are shown at different percentiles of postneonatal mortality rate. Since the relationship was nonlinear, the marginal effects vary across the distribution of postneonatal mortality rate. The marginal effects were obtained using the partial derivative of the regression equation with respect to postneonatal mortality rate. The level of postneonatal mortality rate per 100 at each of the percentile is shown in parentheses (considering the pooled samples for both outcomes). 95% confidence intervals adjusted for clustering within primary sampling units are shown in brackets. Postneonatal mortality rate per 100 person-years within a 50 kilometer radius was linked to the period of infancy.

Table S19. Results from linear regression models: Postneonatal mortality rate restricted to children born 10 years before survey

Outcome->	Height-for-age z-score			School attendance		
Fixed-effects->	Survey	Neighborhood	Sibling	Survey	Neighborhood	Sibling
Independent variables						
Postneonatal mortality rate (per 100)	-.055***	-.016***	-.025**	-.02***	-.0025***	-.0015
	[-.063, -.047]	[-.028, -.0048]	[-.049, -.000024]	[-.022, -.018]	[-.0038, -.0012]	[-.0037, .00063]
Postneonatal mortality rate (per 100) squared	.0018***	.00089**	.0019**	.00085***	.00033***	.00029***
	[.0011, .0025]	[.000032, .0017]	[.00013, .0036]	[.00073, .00098]	[.00023, .00044]	[.00011, .00047]
Firstborn	.22***	.2***	.21***	.0055**	.0029	-.018***
	[.19, .25]	[.17, .24]	[.11, .31]	[.00021, .011]	[-.0021, .0079]	[-.027, -.0086]
Birth interval (months)	.0046***	.0046***	.0063***	.00021***	.00012***	-.00027***
	[.0041, .0051]	[.0041, .0051]	[.0042, .0085]	[.00013, .00029]	[.000043, .00019]	[-.00045, -.000089]
Birth order	-.2***	-.21***	-1.1***	.0086***	-.0005	-.022***
	[-.22, -.18]	[-.23, -.18]	[-1.2, -.96]	[.0067, .011]	[-.0023, .0013]	[-.03, -.014]
Age (months)	-.053***	-.055***	-.084***	.016***	.014***	.015***
	[-.056, -.05]	[-.059, -.051]	[-.093, -.075]	[.015, .016]	[.014, .015]	[.014, .015]
Age (months) squared	.00068***	.0007***	.00069***	-.000053***	-.000053***	-.000056***
	[.00064, .00073]	[.00065, .00075]	[.00058, .0008]	[-.000055, -.000051]	[-.000054, -.000051]	[-.000058, -.000053]
Female	.16***	.16***	.17***	-.016***	-.017***	-.019***
	[.15, .18]	[.15, .18]	[.13, .22]	[-.019, -.013]	[-.02, -.014]	[-.023, -.014]
Mother's age at birth (years)	.065***	.046***		.0051***	-.00084	
	[.055, .075]	[.035, .058]		[.0028, .0073]	[-.0029, .0012]	
Mother's age at birth (years) squared	-.0007***	-.0005***		-.000075***	.00001	
	[-.00088, -.00053]	[-.00069, -.00031]		[-.00012, -.000034]	[-.000028, .000048]	
Mother's education (years)	.074***	.035***		.023***	.0089***	
	[.071, .077]	[.032, .038]		[.022, .024]	[.0085, .0094]	
Number of siblings	.16***	.18***		-.016***	-.0044***	
	[.14, .18]	[.16, .2]		[-.018, -.015]	[-.0057, -.003]	
Twin	-.56***	-.6***	-.74***	.025***	.012**	.01
	[-.62, -.51]	[-.66, -.53]	[-.93, -.54]	[.015, .035]	[.0025, .021]	[-.0081, .029]
Constant	-1.7***	-1.8***	-1.7***	.81***	.78***	.79***
	[-1.7, -1.7]	[-1.8, -1.7]	[-1.8, -1.7]	[.81, .82]	[.77, .78]	[.78, .79]
R squared	0.093	0.273	0.701	0.220	0.435	0.710
Observations	172,149	169,281	47,516	292,280	290,681	184,617

Notes: ***p<0.01; **p<0.05; *p<0.1. Linear regression coefficients are shown. Postneonatal mortality rate was centered around percentile 25 (considering the pooled samples for both outcomes) and all covariates were mean-centered (using means for all valid observations in each analysis): therefore, the constant shows the mean outcome when covariates were at their means and postneonatal mortality rate was at percentile 25, and the coefficient of the linear term for postneonatal mortality rate shows the marginal effect at percentile 25. 95% confidence intervals (shown in brackets) and p-values were adjusted for clustering within primary sampling units. Postneonatal mortality rate per 100 person-years within a 50 kilometer radius was linked to the period of infancy.

Figure S15. Marginal effects of postneonatal mortality rate on height-for-age across the distribution of postneonatal mortality rate: Postneonatal mortality rate restricted to children born 10 years before survey

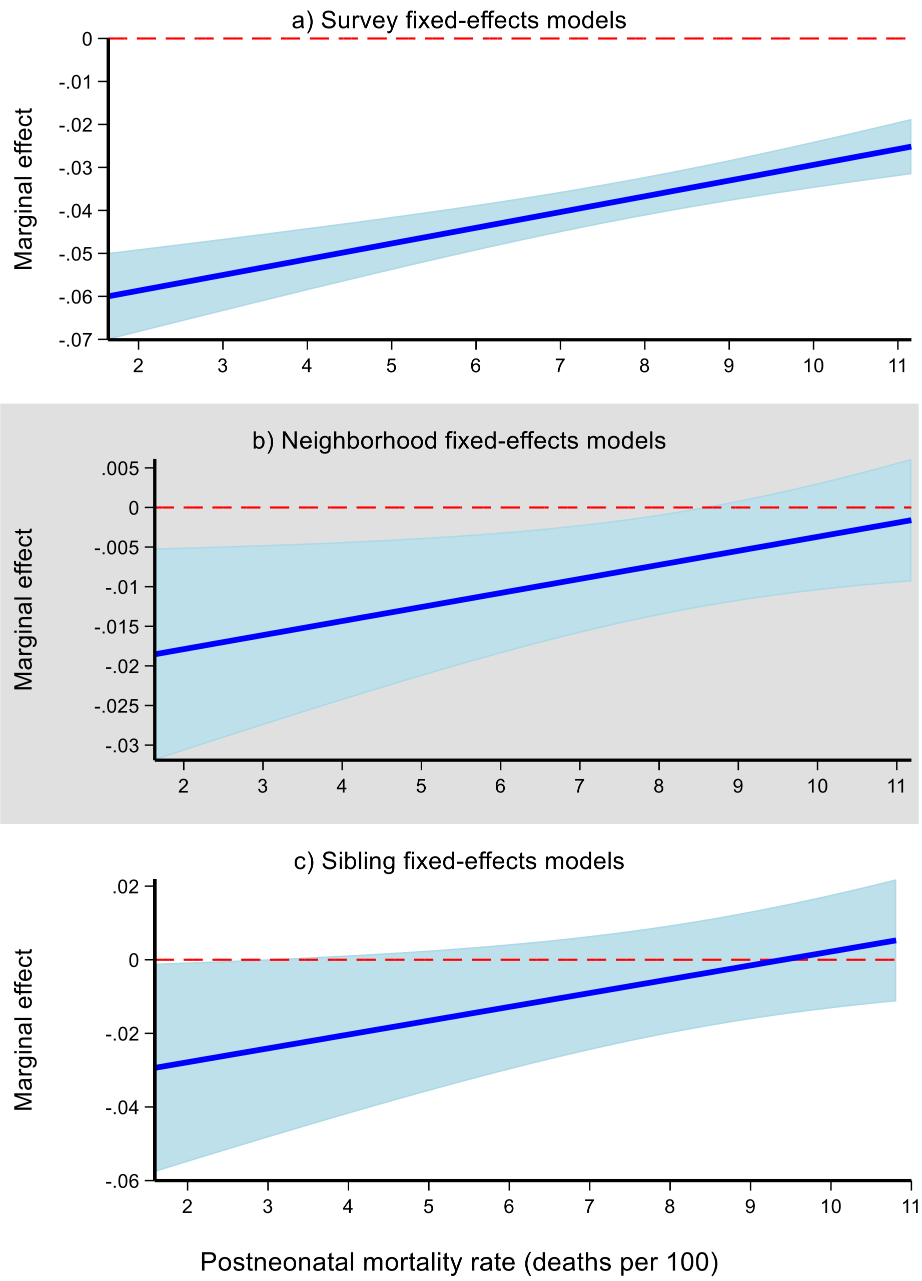

Notes: The values on the y-axis indicate marginal effects: that is, change in the outcome for a single death increase in postneonatal mortality rate per 100 person-years. Since the relationship was nonlinear, the marginal effects vary across the distribution of postneonatal mortality rate. The marginal effects were obtained using the partial derivative of the regression equation with respect to postneonatal mortality rate. The plot was restricted to postneonatal mortality rate between the 5th and 95th percentile (considering the pooled samples for both outcomes). 95% confidence intervals adjusted for clustering within primary sampling units are shown. Postneonatal mortality rate per 100 person-years within a 50 kilometer radius was linked to the period of infancy.

Figure S16. Marginal effects of postneonatal mortality rate on school attendance across the distribution of postneonatal mortality rate: Postneonatal mortality rate restricted to children born 10 years before survey

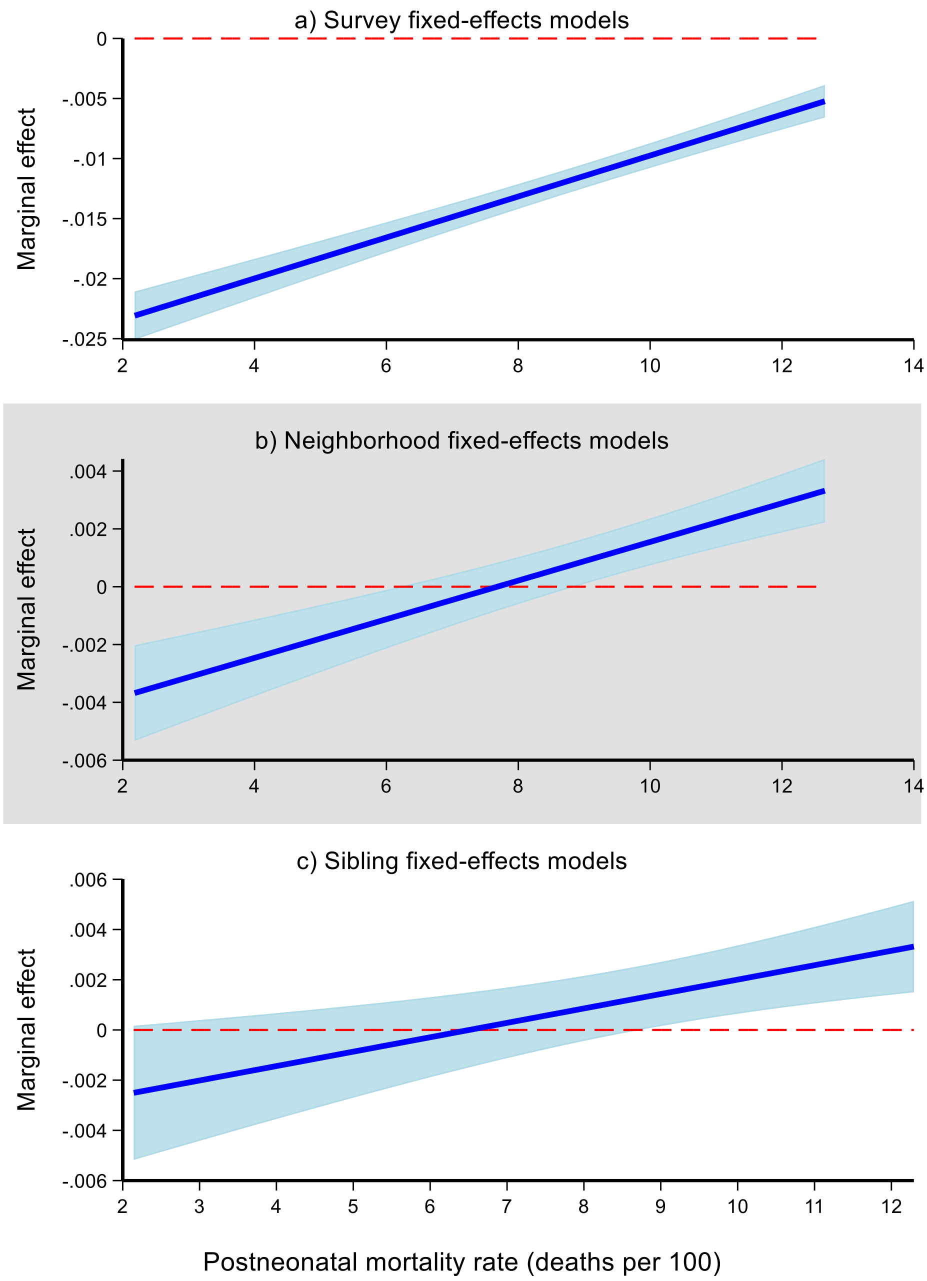

Notes: The values on the y-axis indicate marginal effects: that is, change in the outcome for a single death increase in postneonatal mortality rate per 100 person-years. Since the relationship was nonlinear, the marginal effects vary across the distribution of postneonatal mortality rate. The marginal effects were obtained using the partial derivative of the regression equation with respect to postneonatal mortality rate. The plot was restricted to postneonatal mortality rate between the 5th and 95th percentile (considering the pooled samples for both outcomes). 95% confidence intervals adjusted for clustering within primary sampling units are shown. Postneonatal mortality rate per 100 person-years within a 50 kilometer radius was linked to the period of infancy.

Table S20. Marginal effects of postneonatal mortality rate on height-for-age and school attendance at different percentiles of postneonatal mortality rate: Postneonatal mortality rate restricted to children born 10 years before survey

Outcome->	Height-for-age z-score			School attendance		
Fixed-effects->	Survey	Neighborhood	Sibling	Survey	Neighborhood	Sibling
Percentiles of postneonatal mortality rate (per 100)						
p1 (1.3)	-.062***	-.019***	-.031**	-.024***	-.0042***	-.0029**
	[-.072, -.051]	[-.033, -.0053]	[-.061, -.0014]	[-.027, -.022]	[-.006, -.0024]	[-.0058, -.000025]
p5 (1.9)	-.06***	-.019***	-.029**	-.023***	-.0037***	-.0025*
	[-.07, -.05]	[-.032, -.0052]	[-.058, -.0011]	[-.025, -.021]	[-.0053, -.002]	[-.0052, .00017]
p10 (2.4)	-.059***	-.018***	-.028**	-.022***	-.0033***	-.0022*
	[-.068, -.049]	[-.031, -.0051]	[-.055, -.0008]	[-.024, -.02]	[-.0049, -.0018]	[-.0047, .0003]
p25 (3.5)	-.055***	-.016***	-.025**	-.02***	-.0025***	-.0015
	[-.063, -.047]	[-.028, -.0048]	[-.049, -.000024]	[-.022, -.018]	[-.0038, -.0012]	[-.0037, .00063]
p50 (5.5)	-.049***	-.013***	-.018*	-.017***	-.0012**	-.00044
	[-.055, -.042]	[-.022, -.004]	[-.039, .0018]	[-.018, -.015]	[-.0022, -.00016]	[-.0021, .0012]
p75 (7.9)	-.04***	-.0089**	-.0099	-.013***	.00046	.00093
	[-.045, -.035]	[-.016, -.0021]	[-.026, .0058]	[-.014, -.012]	[-.00033, .0013]	[-.00036, .0022]
p90 (10.4)	-.031***	-.0046	-.00084	-.0082***	.0021***	.0023***
	[-.036, -.026]	[-.011, .0019]	[-.016, .014]	[-.0093, -.0071]	[.0013, .003]	[.00089, .0038]
p95 (12.2)	-.025***	-.0016	.0053	-.0052***	.0033***	.0033***
	[-.032, -.019]	[-.0093, .0061]	[-.011, .022]	[-.0066, -.0039]	[.0022, .0044]	[.0015, .0051]
p99 (15.9)	-.011*	.0055	.02	.0009	.0057***	.0054***
	[-.022, .00074]	[-.0075, .018]	[-.0061, .046]	[-.0012, .003]	[.004, .0074]	[.0025, .0083]

Notes: ***p<0.01; **p<0.05; *p<0.1. Change in outcome for a single increase in postneonatal mortality rate per 100 person-years are shown at different percentiles of postneonatal mortality rate. Since the relationship was nonlinear, the marginal effects vary across the distribution of postneonatal mortality rate. The marginal effects were obtained using the partial derivative of the regression equation with respect to postneonatal mortality rate. The level of postneonatal mortality rate per 100 at each of the percentile is shown in parentheses (considering the pooled samples for both outcomes). 95% confidence intervals adjusted for clustering within primary sampling units are shown in brackets. Postneonatal mortality rate per 100 person-years within a 50 kilometer radius was linked to the period of infancy.

Table S21. Results from linear regression models: Neighborhood fixed-effects on the sibling sample

Outcome->	Height-for-age z-score			School attendance		
Fixed-effects->	Survey	Neighborhood	Neighborhood†	Survey	Neighborhood	Neighborhood†
Independent variables						
Postneonatal mortality rate (per 100)	-.06***	-.015***	-.013	-.021***	-.0016***	-.0018***
	[-.068, -.052]	[-.026, -.0046]	[-.031, .006]	[-.022, -.019]	[-.0028, -.00046]	[-.0032, -.00047]
Postneonatal mortality rate (per 100) squared	.0019***	.0011**	.0011	.00081***	.00034***	.00033***
	[.0012, .0026]	[.00021, .0019]	[-.00032, .0025]	[.00068, .00094]	[.00023, .00044]	[.00021, .00045]
Firstborn	.22***	.2***	.34***	.00087	-.000099	-.0039
	[.19, .25]	[.17, .24]	[.27, .4]	[-.0035, .0053]	[-.0041, .0039]	[-.0089, .0011]
Birth interval (months)	.0047***	.0047***	.0091***	.00016***	.00006*	.000097**
	[.0043, .0051]	[.0042, .0051]	[.0078, .01]	[.00009, .00023]	[-2.0e-06, .00012]	[.000013, .00018]
Birth order	-.22***	-.23***	-.42***	.01***	.00075	.00061
	[-.24, -.2]	[-.25, -.21]	[-.47, -.38]	[.0087, .012]	[-.00066, .0022]	[-.0011, .0023]
Age (months)	-.055***	-.058***	-.054***	.014***	.014***	.014***
	[-.058, -.052]	[-.061, -.054]	[-.06, -.048]	[.014, .015]	[.013, .014]	[.014, .015]
Age (months) squared	.0007***	.00072***	.00061***	-.000049***	-.000049***	-.000052***
	[.00066, .00074]	[.00068, .00077]	[.00053, .00068]	[-.00005, -.000048]	[-.00005, -.000048]	[-.000053, -.00005]
Female	.16***	.16***	.16***	-.02***	-.022***	-.023***
	[.14, .17]	[.14, .17]	[.13, .19]	[-.023, -.018]	[-.024, -.02]	[-.026, -.02]
Mother's age at birth (years)	.065***	.047***	.025*	.0032***	-.0017**	-.0046***
	[.055, .074]	[.037, .057]	[-.0032, .054]	[.0013, .0051]	[-.0034, -.000035]	[-.0068, -.0024]
Mother's age at birth (years) squared	-.0007***	-.0005***	-.000092	-.000054***	.000022	.000071***
	[-.00085, -.00054]	[-.00067, -.00032]	[-.00059, .00041]	[-.000089, -.000018]	[-9.8e-06, .000053]	[.000031, .00011]
Mother's education (years)	.073***	.035***	.03***	.024***	.0091***	.0094***
	[.071, .076]	[.032, .038]	[.022, .038]	[.023, .024]	[.0087, .0095]	[.0089, .0099]
Number of siblings	.18***	.2***	.39***	-.016***	-.0048***	-.0052***
	[.16, .2]	[.18, .22]	[.34, .43]	[-.018, -.015]	[-.0059, -.0038]	[-.0066, -.0039]
Twin	-.58***	-.62***	-.67***	.025***	.0095**	.013***
	[-.64, -.53]	[-.68, -.56]	[-.76, -.58]	[.017, .033]	[.0018, .017]	[.0043, .021]
Constant	-1.7***	-1.8***	-1.8***	.8***	.76***	.76***
	[-1.7, -1.7]	[-1.8, -1.8]	[-1.8, -1.7]	[.79, .8]	[.76, .76]	[.76, .76]
R squared	0.093	0.267	0.396	0.222	0.433	0.440
Observations	199,417	196,543	61,851	442,405	441,159	329,141

Notes: ***p<0.01; **p<0.05; *p<0.1. Linear regression coefficients are shown. Postneonatal mortality rate was centered around percentile 25 (considering the pooled samples for both outcomes) and all covariates were mean-centered (using means for all valid observations in each analysis): therefore, the constant shows the mean outcome when covariates were at their means and postneonatal mortality rate was at percentile 25, and the coefficient of the linear term for postneonatal mortality rate shows the marginal effect at percentile 25. 95% confidence intervals (shown in brackets) and p-values were adjusted for clustering within primary sampling units. †Sample restricted to children with a sibling with valid data. Postneonatal mortality rate per 100 person-years within a 50 kilometer radius was linked to the period of infancy.

Figure S17. Marginal effects of postneonatal mortality rate on height-for-age across the distribution of postneonatal mortality rate: Neighborhood fixed-effects on the sibling sample

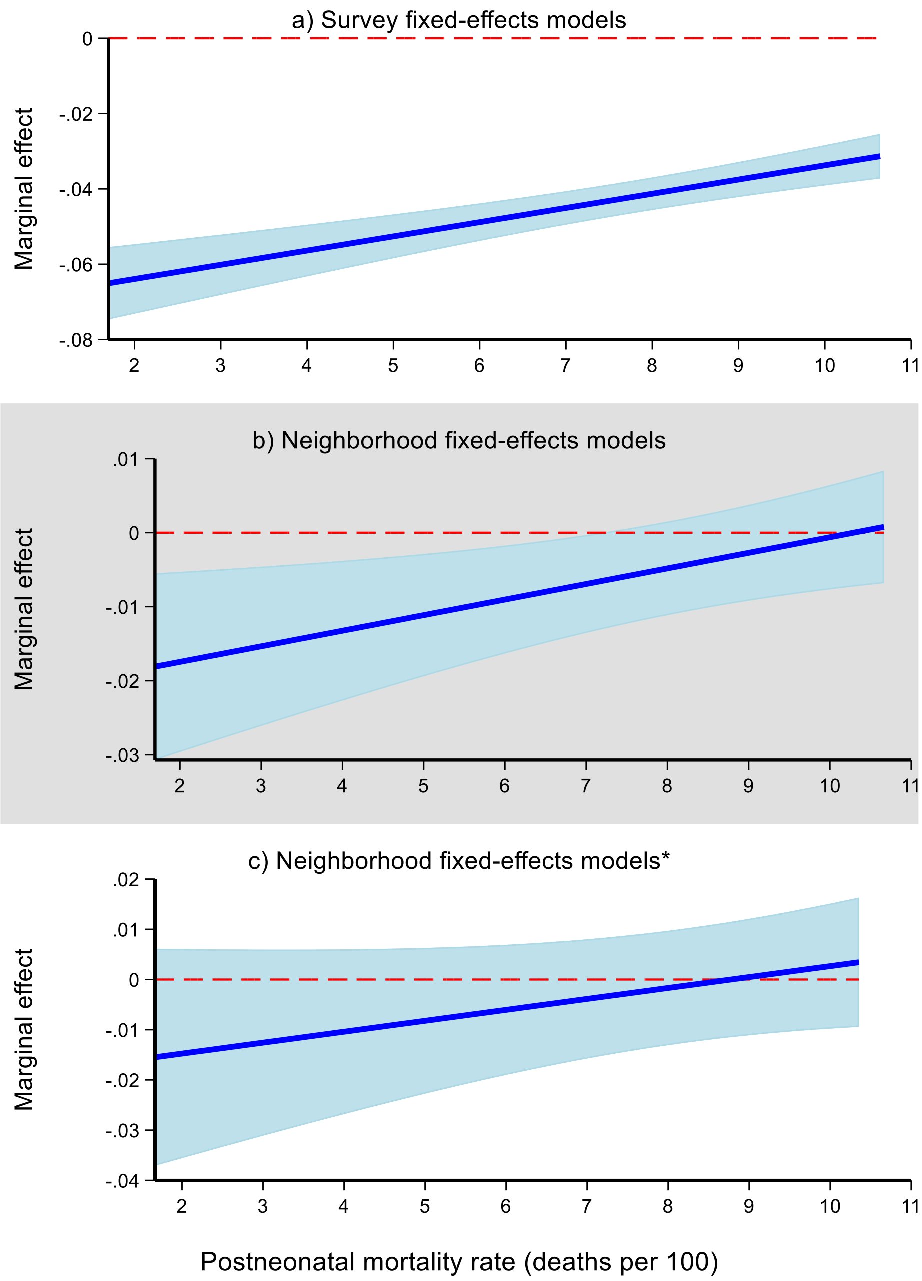

Notes: The values on the y-axis indicate marginal effects: that is, change in the outcome for a single death increase in postneonatal mortality rate per 100 person-years. Since the relationship was nonlinear, the marginal effects vary across the distribution of postneonatal mortality rate. The marginal effects were obtained using the partial derivative of the regression equation with respect to postneonatal mortality rate. The plot was restricted to postneonatal mortality rate between the 5th and 95th percentile (considering the pooled samples for both outcomes). 95% confidence intervals adjusted for clustering within primary sampling units are shown. *Sample restricted to children with a sibling with valid data. Postneonatal mortality rate per 100 person-years within a 50 kilometer radius was linked to the period of infancy.

Figure S18. Marginal effects of postneonatal mortality rate on school attendance across the distribution of postneonatal mortality rate: Neighborhood fixed-effects on the sibling sample

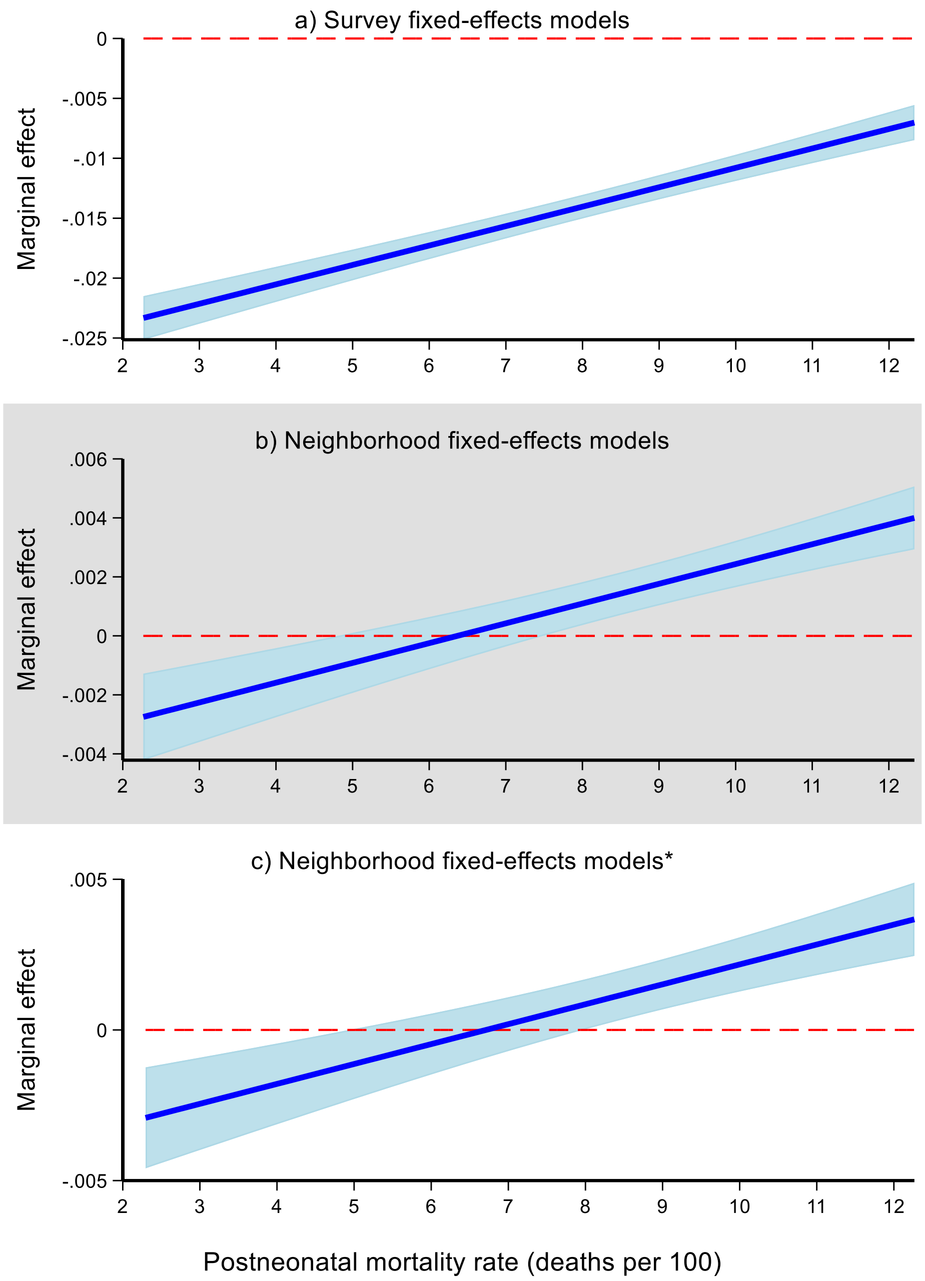

Notes: The values on the y-axis indicate marginal effects: that is, change in the outcome for a single death increase in postneonatal mortality rate per 100 person-years. Since the relationship was nonlinear, the marginal effects vary across the distribution of postneonatal mortality rate. The marginal effects were obtained using the partial derivative of the regression equation with respect to postneonatal mortality rate. The plot was restricted to postneonatal mortality rate between the 5th and 95th percentile (considering the pooled samples for both outcomes). 95% confidence intervals adjusted for clustering within primary sampling units are shown. *Sample restricted to children with a sibling with valid data. Postneonatal mortality rate per 100 person-years within a 50 kilometer radius was linked to the period of infancy.

Table S22. Marginal effects of postneonatal mortality rate on height-for-age and school attendance at different percentiles of postneonatal mortality rate: Neighborhood fixed-effects on the sibling sample

Outcome->	Height-for-age z-score			School attendance		
Fixed-effects->	Survey	Neighborhood	Neighborhood†	Survey	Neighborhood	Neighborhood†
Percentiles of postneonatal mortality rate (per 100)						
p1 (1.4)	-.067***	-.019***	-.016	-.025***	-.0032***	-.0034***
	[-.077, -.057]	[-.033, -.0058]	[-.039, .0062]	[-.027, -.023]	[-.0048, -.0016]	[-.0052, -.0016]
p5 (2.0)	-.065***	-.018***	-.015	-.023***	-.0027***	-.0029***
	[-.075, -.055]	[-.031, -.0055]	[-.037, .0061]	[-.025, -.022]	[-.0042, -.0013]	[-.0046, -.0012]
p10 (2.5)	-.063***	-.017***	-.015	-.023***	-.0024***	-.0026***
	[-.073, -.054]	[-.029, -.0052]	[-.035, .0061]	[-.024, -.021]	[-.0038, -.001]	[-.0042, -.001]
p25 (3.6)	-.06***	-.015***	-.013	-.021***	-.0016***	-.0018***
	[-.068, -.052]	[-.026, -.0046]	[-.031, .006]	[-.022, -.019]	[-.0028, -.00046]	[-.0032, -.00047]
p50 (5.4)	-.054***	-.012***	-.0093	-.018***	-.00038	-.00058
	[-.06, -.048]	[-.02, -.0032]	[-.025, .0061]	[-.019, -.016]	[-.0013, .00053]	[-.0016, .00045]
p75 (7.8)	-.046***	-.0073**	-.0047	-.014***	.0012***	.00094**
	[-.05, -.041]	[-.014, -.00056]	[-.017, .0075]	[-.015, -.013]	[.00047, .0019]	[.00011, .0018]
p90 (10.2)	-.037***	-.0026	-1.8e-06	-.0098***	.0028***	.0025***
	[-.042, -.033]	[-.0091, .0039]	[-.011, .011]	[-.011, -.0087]	[.002, .0037]	[.0016, .0035]
p95 (11.9)	-.031***	.00078	.0034	-.007***	.004***	.0037***
	[-.037, -.025]	[-.0068, .0084]	[-.0094, .016]	[-.0085, -.0056]	[.0029, .0051]	[.0025, .0049]
p99 (15.8)	-.017***	.0089	.012	-.00067	.0066***	.0063***
	[-.027, -.0063]	[-.0036, .021]	[-.0085, .032]	[-.003, .0017]	[.0049, .0084]	[.0043, .0083]

Notes: ***p<0.01; **p<0.05; *p<0.1. Change in outcome for a single increase in postneonatal mortality rate per 100 person-years are shown at different percentiles of postneonatal mortality rate. Since the relationship was nonlinear, the marginal effects vary across the distribution of postneonatal mortality rate. The marginal effects were obtained using the partial derivative of the regression equation with respect to postneonatal mortality rate. The level of postneonatal mortality rate per 100 at each of the percentile is shown in parentheses (considering the pooled samples for both outcomes). 95% confidence intervals adjusted for clustering within primary sampling units are shown in brackets. †Sample restricted to children with a sibling with valid data. Postneonatal mortality rate per 100 person-years within a 50 kilometer radius was linked to the period of infancy.

Table S23. Results from linear regression models: Postneonatal mortality rate indexed to a 12 month period starting at conception

Outcome->	Height-for-age z-score			School attendance		
Fixed-effects->	Survey	Neighborhood	Sibling	Survey	Neighborhood	Sibling
Independent variables						
Postneonatal mortality rate (per 100)	-.065***	-.0034	-.088	-.021***	-.000034	-.00063
	[-.081, -.049]	[-.034, .027]	[-.2, .023]	[-.024, -.018]	[-.0032, .0031]	[-.0089, .0076]
Postneonatal mortality rate (per 100) squared	.0025***	.0012	.0055	.00084***	.0001	-.00014
	[.0011, .0039]	[-.0011, .0036]	[-.0032, .014]	[.00059, .0011]	[-.00017, .00037]	[-.00088, .0006]
Firstborn	.23***	.27***	.23	.015***	.013**	-.00052
	[.16, .3]	[.17, .36]	[-.33, .79]	[.0038, .026]	[.00088, .026]	[-.036, .035]
Birth interval (months)	.0056***	.0061***	.012**	.00037***	.00024**	-.00031
	[.0045, .0067]	[.0046, .0076]	[9.2e-06, .024]	[.00021, .00054]	[.000045, .00043]	[-.001, .00042]
Birth order	-.28***	-.29***	-1.5***	.0087***	-.001	-.016
	[-.32, -.24]	[-.35, -.24]	[-2.2, -.87]	[.0054, .012]	[-.0047, .0027]	[-.046, .014]
Age (months)	-.08***	-.091***	-.12***	.016***	.015***	.016***
	[-.089, -.07]	[-.11, -.078]	[-.18, -.067]	[.015, .016]	[.014, .016]	[.013, .018]
Age (months) squared	.0011***	.0013***	.0011***	-.000052***	-.000052***	-.000057***
	[.00094, .0013]	[.001, .0015]	[.00027, .0019]	[-.000056, -.000048]	[-.000056, -.000048]	[-.000067, -.000048]
Female	.19***	.18***	.08	-.026***	-.03***	-.047***
	[.15, .22]	[.13, .23]	[-.15, .31]	[-.031, -.02]	[-.037, -.024]	[-.065, -.028]
Mother's age at birth (years)	.071***	.05***		.0047**	.00021	
	[.05, .092]	[.022, .078]		[.00045, .0089]	[-.0045, .0049]	
Mother's age at birth (years) squared	-.00083***	-.00062***		-.000072*	4.7e-06	
	[-.0012, -.00049]	[-.0011, -.00015]		[-.00015, 5.2e-06]	[-.000082, .000091]	
Mother's education (years)	.073***	.031***		.027***	.011***	
	[.067, .078]	[.021, .04]		[.026, .028]	[.0095, .012]	
Number of siblings	.24***	.28***		-.014***	-.0044***	
	[.2, .28]	[.22, .34]		[-.017, -.012]	[-.0074, -.0015]	
Twin	-.53***	-.59***	-1.1**	.031***	.0088	.051
	[-.68, -.38]	[-.79, -.4]	[-2.1, -.057]	[.0099, .051]	[-.015, .032]	[-.016, .12]
Constant	-1.8***	-1.9***	-1.6***	.76***	.72***	.7***
	[-1.8, -1.7]	[-2, -1.9]	[-1.8, -1.5]	[.76, .77]	[.71, .72]	[.69, .72]
R squared	0.086	0.355	0.721	0.240	0.525	0.764
Observations	37,354	32,139	2,065	77,325	72,513	17,773

Notes: ***p<0.01; **p<0.05; *p<0.1. Linear regression coefficients are shown. Postneonatal mortality rate was centered around percentile 25 (considering the pooled samples for both outcomes) and all covariates were mean-centered (using means for all valid observations in each analysis): therefore, the constant shows the mean outcome when covariates were at their means and postneonatal mortality rate was at percentile 25, and the coefficient of the linear term for postneonatal mortality rate shows the marginal effect at percentile 25. 95% confidence intervals (shown in brackets) and p-values were adjusted for clustering within primary sampling units. Postneonatal mortality rate per 100 person-years within a 50 kilometer radius was linked to a 12 month period starting at conception (ie, 9 months before birth).

Figure S19. Marginal effects of postneonatal mortality rate on height-for-age across the distribution of postneonatal mortality rate: Postneonatal mortality rate indexed to a 12 month period starting at conception

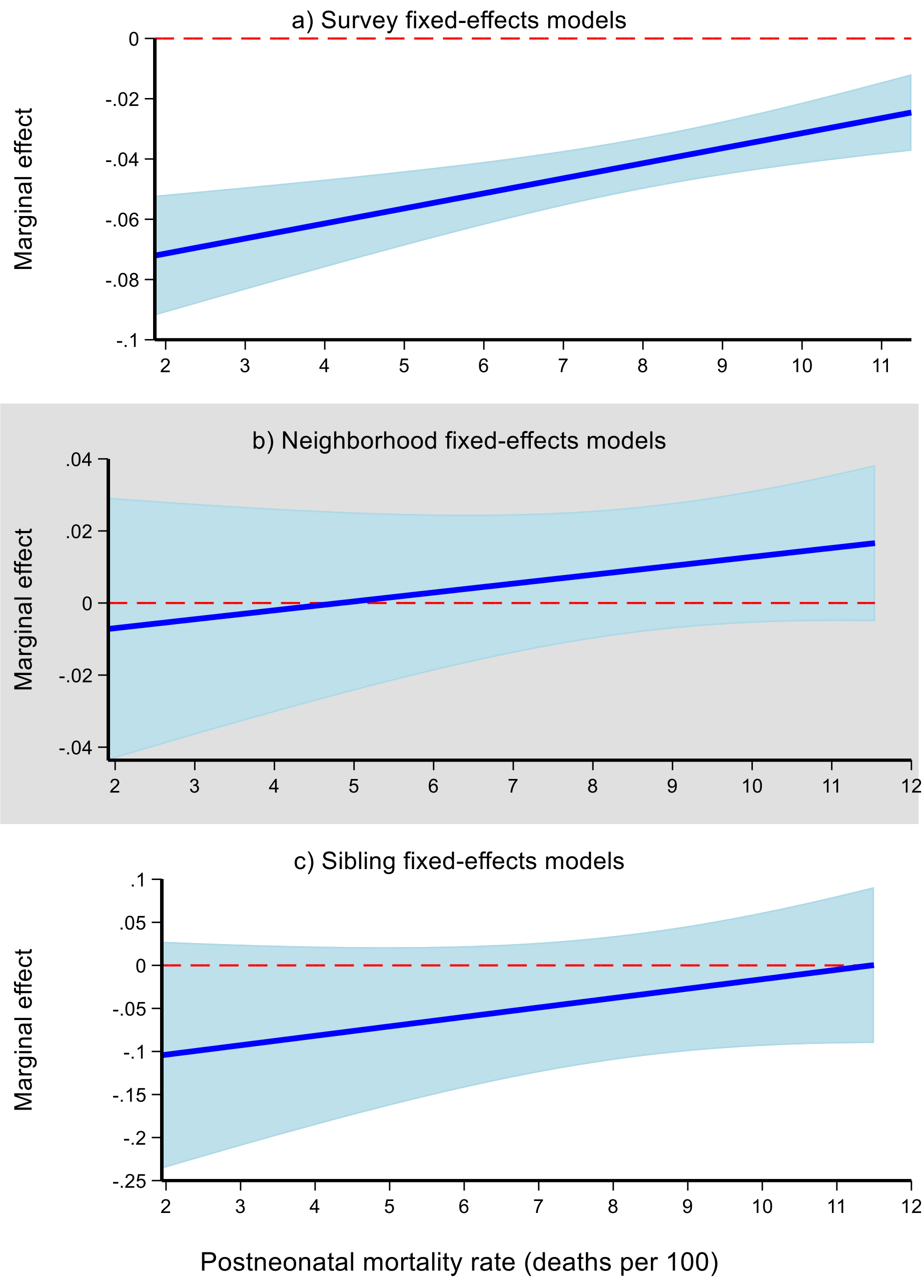

Notes: The values on the y-axis indicate marginal effects: that is, change in the outcome for a single death increase in postneonatal mortality rate per 100 person-years. Since the relationship was nonlinear, the marginal effects vary across the distribution of postneonatal mortality rate. The marginal effects were obtained using the partial derivative of the regression equation with respect to postneonatal mortality rate. The plot was restricted to postneonatal mortality rate between the 5th and 95th percentile (considering the pooled samples for both outcomes). 95% confidence intervals adjusted for clustering within primary sampling units are shown. Postneonatal mortality rate per 100 person-years within a 50 kilometer radius was linked to a 12 month period starting at conception (ie, 9 months before birth).

Figure S20. Marginal effects of postneonatal mortality rate on school attendance across the distribution of postneonatal mortality rate: Postneonatal mortality rate indexed to a 12 month period starting at conception

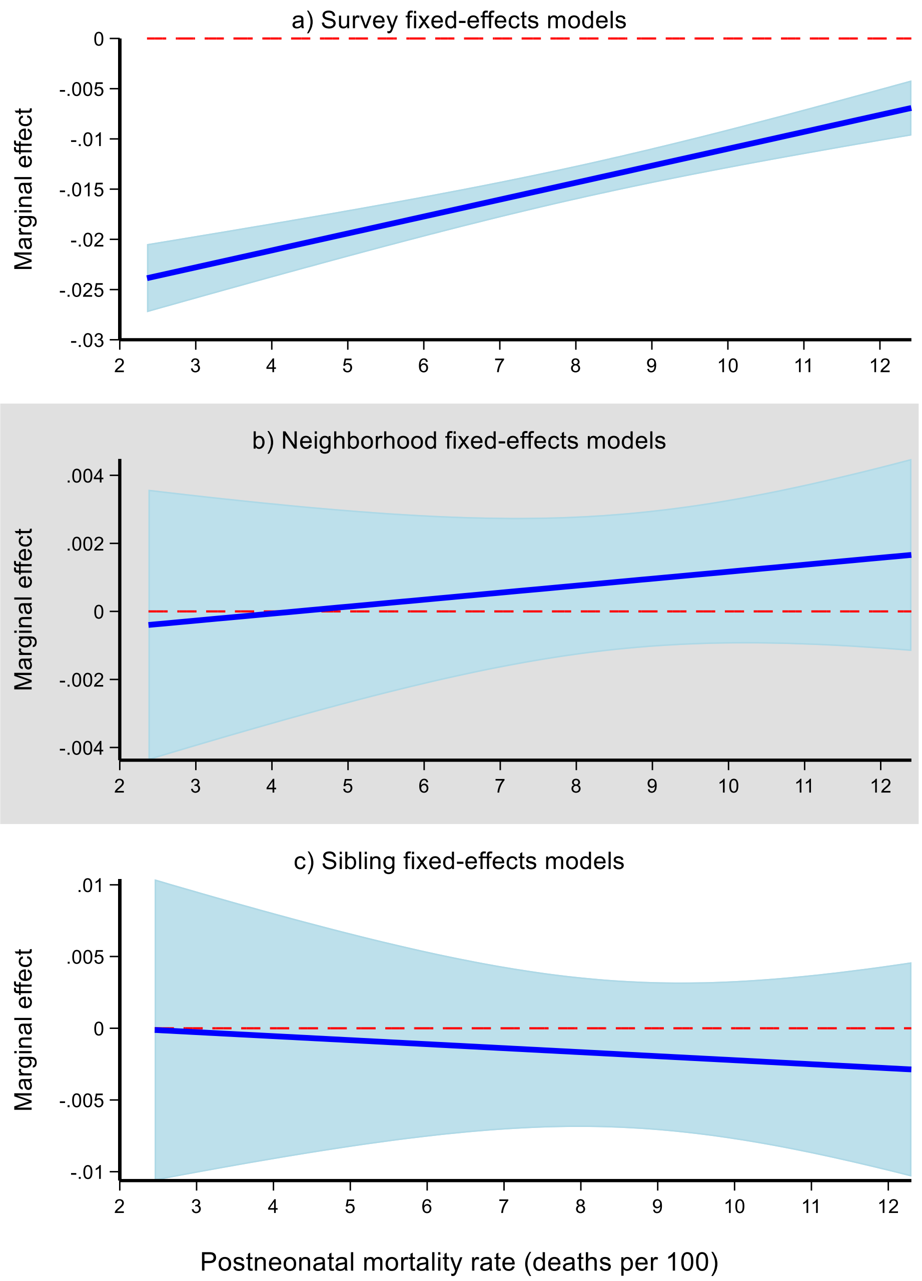

Notes: The values on the y-axis indicate marginal effects: that is, change in the outcome for a single death increase in postneonatal mortality rate per 100 person-years. Since the relationship was nonlinear, the marginal effects vary across the distribution of postneonatal mortality rate. The marginal effects were obtained using the partial derivative of the regression equation with respect to postneonatal mortality rate. The plot was restricted to postneonatal mortality rate between the 5th and 95th percentile (considering the pooled samples for both outcomes). 95% confidence intervals adjusted for clustering within primary sampling units are shown. Postneonatal mortality rate per 100 person-years within a 50 kilometer radius was linked to a 12 month period starting at conception (ie, 9 months before birth).

Table S24. Marginal effects of postneonatal mortality rate on height-for-age and school attendance at different percentiles of postneonatal mortality rate: Postneonatal mortality rate indexed to a 12 month period starting at conception

Outcome->	Height-for-age z-score			School attendance		
Fixed-effects->	Survey	Neighborhood	Sibling	Survey	Neighborhood	Sibling
Percentiles of postneonatal mortality rate (per 100)						
p1 (1.4)	-.075***	-.0087	-.11	-.025***	-.00055	.000094
	[-.096, -.054]	[-.048, .03]	[-.25, .03]	[-.029, -.021]	[-.0049, .0038]	[-.011, .012]
p5 (2.2)	-.072***	-.0072	-.1	-.024***	-.0004	-.00012
	[-.092, -.052]	[-.044, .029]	[-.24, .028]	[-.027, -.02]	[-.0044, .0036]	[-.011, .01]
p10 (2.7)	-.07***	-.006	-.1	-.023***	-.00029	-.00027
	[-.088, -.051]	[-.04, .028]	[-.23, .026]	[-.026, -.02]	[-.004, .0034]	[-.01, .0095]
p25 (3.9)	-.065***	-.0034	-.088	-.021***	-.000034	-.00063
	[-.081, -.049]	[-.034, .027]	[-.2, .023]	[-.024, -.018]	[-.0032, .0031]	[-.0089, .0076]
p50 (5.7)	-.056***	.0012	-.069	-.018***	.00036	-.0011
	[-.068, -.044]	[-.023, .025]	[-.16, .021]	[-.02, -.016]	[-.0021, .0028]	[-.0075, .0052]
p75 (8.0)	-.045***	.0065	-.047	-.014***	.00082	-.0017
	[-.054, -.036]	[-.012, .025]	[-.12, .028]	[-.016, -.012]	[-.0012, .0028]	[-.0069, .0034]
p90 (10.4)	-.034***	.012	-.02	-.0098***	.0013	-.0024
	[-.043, -.024]	[-.0058, .03]	[-.095, .055]	[-.012, -.0077]	[-.00095, .0036]	[-.0083, .0035]
p95 (12.1)	-.025***	.017	.00036	-.0069***	.0017	-.0029
	[-.037, -.012]	[-.0051, .038]	[-.09, .091]	[-.0097, -.0042]	[-.0012, .0045]	[-.01, .0046]
p99 (16.0)	-.0043	.027	.053	-.00045	.0024	-.004
	[-.027, .018]	[-.0097, .063]	[-.1, .21]	[-.0049, .004]	[-.0021, .007]	[-.017, .0086]

Notes: ***p<0.01; **p<0.05; *p<0.1. Change in outcome for a single increase in postneonatal mortality rate per 100 person-years are shown at different percentiles of postneonatal mortality rate. Since the relationship was nonlinear, the marginal effects vary across the distribution of postneonatal mortality rate. The marginal effects were obtained using the partial derivative of the regression equation with respect to postneonatal mortality rate. The level of postneonatal mortality rate per 100 at each of the percentile is shown in parentheses (considering the pooled samples for both outcomes). 95% confidence intervals adjusted for clustering within primary sampling units are shown in brackets. Postneonatal mortality rate per 100 person-years within a 50 kilometer radius was linked to a 12 month period starting at conception (ie, 9 months before birth).

Table S25. Results from regression models: logit models instead of linear regressions

Outcome->	School attendance		
Fixed-effects->	Survey	Neighborhood	Sibling
Independent variables			
Postneonatal mortality rate (per 100)	-.12***	-.000049	-.062***
	[-.13, -.11]	[-.0093, .0092]	[-.073, -.051]
Postneonatal mortality rate (per 100) squared	.0043***	.00076**	.0065***
	[.0036, .0051]	[.000041, .0015]	[.0056, .0075]
Firstborn	.028*	.01	-.1***
	[-.003, .06]	[-.018, .039]	[-.14, -.072]
Birth interval (months)	.00089***	.00024	-.0014***
	[.00039, .0014]	[-.00022, .00069]	[-.0021, -.00079]
Birth order	.067***	.0044	-.085***
	[.057, .078]	[-.0045, .013]	[-.11, -.059]
Age (months)	.097***	.095***	.083***
	[.095, .1]	[.093, .098]	[.08, .085]
Age (months) squared	-.00033***	-.00034***	-.0003***
	[-.00034, -.00032]	[-.00035, -.00033]	[-.00031, -.0003]
Female	-.14***	-.16***	-.16***
	[-.16, -.13]	[-.18, -.14]	[-.18, -.15]
Mother's age at birth (years)	.034***	-.004	
	[.021, .046]	[-.015, .0069]	
Mother's age at birth (years) squared	-.00046***	.000064	
	[-.00069, -.00023]	[-.00014, .00026]	
Mother's education (years)	.24***	.11***	
	[.24, .25]	[.1, .11]	
Number of siblings	-.12***	-.034***	
	[-.13, -.11]	[-.041, -.027]	
Twin	.19***	.089***	.079**
	[.13, .26]	[.03, .15]	[.0096, .15]
Constant	1.9***	1.8***	1.3***
	[1.9, 1.9]	[1.8, 1.8]	[1.3, 1.3]
Observations	442,405	441,159	323,830

Notes: ***p<0.01; **p<0.05; *p<0.1. Coefficients are shown. Postneonatal mortality rate was centered around percentile 25 (considering the pooled samples for both outcomes) and all covariates were mean-centered (using means for all valid observations in each analysis): therefore, the constant shows the mean outcome when covariates were at their means and postneonatal mortality rate was at percentile 25, and the coefficient of the linear term for postneonatal mortality rate shows the marginal effect at percentile 25. 95% confidence intervals (shown in brackets) and p-values were adjusted for clustering within primary sampling units. Mundlak-fixed-effects were used, where a survey, neighborhood, or sibling specific means of all independent variables were added as independent variables to the models. Postneonatal mortality rate per 100 person-years within a 50 kilometer radius was linked to the period of infancy.

Figure S21. Marginal effects of postneonatal mortality rate on school attendance across the distribution of postneonatal mortality rate: logit models instead of linear regressions

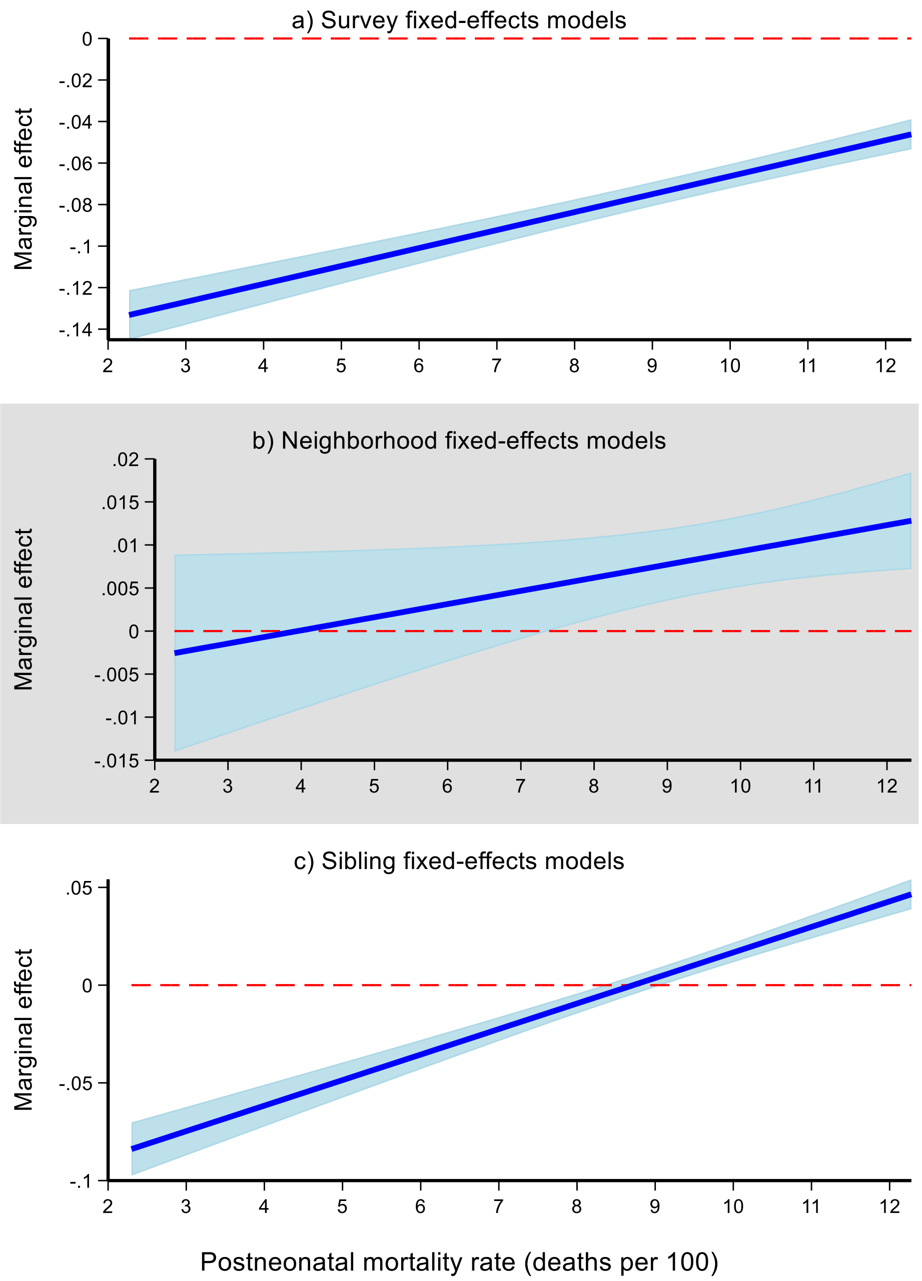

Notes: The values on the y-axis indicate marginal effects: that is, change in the outcome for a single death increase in postneonatal mortality rate per 100 person-years. Since the relationship was nonlinear, the marginal effects vary across the distribution of postneonatal mortality rate. The marginal effects were obtained using the partial derivative of the regression equation with respect to postneonatal mortality rate. The plot was restricted to postneonatal mortality rate between the 5th and 95th percentile (considering the pooled samples for both outcomes). 95% confidence intervals adjusted for clustering within primary sampling units are shown. Mundlak-fixed-effects were used, where a survey, neighborhood, or sibling specific means of all independent variables were added as independent variables to the models. Postneonatal mortality rate per 100 person-years within a 50 kilometer radius was linked to the period of infancy.

Table S26. Marginal effects of postneonatal mortality rate on school attendance at different percentiles of postneonatal mortality rate: logit models instead of linear regressions

Outcome->	School attendance		
Fixed-effects->	Survey	Neighborhood	Sibling
Percentiles of postneonatal mortality rate (per 100)			
p1 (1.4)	-.016***	-.00052	-.014***
	[-.018, -.015]	[-.0023, .0012]	[-.016, -.012]
p5 (2.0)	-.016***	-.00036	-.013***
	[-.018, -.015]	[-.002, .0012]	[-.015, -.011]
p10 (2.5)	-.016***	-.00026	-.012***
	[-.018, -.015]	[-.0018, .0013]	[-.014, -.01]
p25 (3.6)	-.016***	-6.9e-06	-.01***
	[-.017, -.015]	[-.0013, .0013]	[-.012, -.0085]
p50 (5.4)	-.015***	.0004	-.0065***
	[-.016, -.014]	[-.00057, .0014]	[-.0078, -.0051]
p75 (7.8)	-.013***	.0009***	-.0013***
	[-.014, -.012]	[.00026, .0015]	[-.0022, -.00044]
p90 (10.2)	-.01***	.0014***	.0042***
	[-.011, -.009]	[.00082, .002]	[.0033, .0052]
p95 (11.9)	-.0077***	.0018***	.0079***
	[-.0089, -.0064]	[.001, .0026]	[.0067, .0092]
p99 (15.8)	-.0021**	.0026***	.014***
	[-.0041, -.000076]	[.0012, .004]	[.013, .016]

Notes: ***p<0.01; **p<0.05; *p<0.1. Change in outcome for a single increase in postneonatal mortality rate per 100 person-years are shown at different percentiles of postneonatal mortality rate. Since the relationship was nonlinear, the marginal effects vary across the distribution of postneonatal mortality rate. The marginal effects were obtained using the partial derivative of the regression equation with respect to postneonatal mortality rate. The level of postneonatal mortality rate per 100 at each of the percentile is shown in parentheses (considering the pooled samples for both outcomes). 95% confidence intervals adjusted for clustering within primary sampling units are shown in brackets. Mundlak-fixed-effects were used, where a survey, neighborhood, or sibling specific means of all independent variables were added as independent variables to the models. Postneonatal mortality rate per 100 person-years within a 50 kilometer radius was linked to the period of infancy.

Table S27. Results from linear regression models: Postneonatal mortality rate converted to natural log scale

Outcome->	Height-for-age z-score			School attendance		
Fixed-effects->	Survey	Neighborhood	Sibling	Survey	Neighborhood	Sibling
Independent variables						
Postneonatal mortality rate (per 100)	-.21***	-.07***	-.12**	-.087***	-.011***	-.011***
	[-.24, -.19]	[-.11, -.031]	[-.21, -.022]	[-.092, -.082]	[-.016, -.0067]	[-.018, -.0042]
Postneonatal mortality rate (per 100) squared	-.04***	.034**	.067*	-.0057*	.023***	.026***
	[-.064, -.016]	[.0029, .064]	[-.0062, .14]	[-.011, .000055]	[.019, .027]	[.02, .033]
Firstborn	.22***	.2***	.24***	.00083	-.00013	-.015***
	[.19, .25]	[.17, .24]	[.14, .33]	[-.0036, .0052]	[-.0042, .0039]	[-.022, -.0082]
Birth interval (months)	.0047***	.0047***	.007***	.00016***	.000059*	-.00024***
	[.0043, .0051]	[.0042, .0051]	[.005, .0089]	[.00009, .00023]	[-3.1e-06, .00012]	[-.00038, -.000098]
Birth order	-.22***	-.23***	-1.1***	.01***	.00083	-.015***
	[-.24, -.2]	[-.25, -.21]	[-1.2, -.99]	[.0088, .012]	[-.00058, .0022]	[-.021, -.0091]
Age (months)	-.055***	-.058***	-.088***	.015***	.014***	.015***
	[-.058, -.052]	[-.061, -.054]	[-.096, -.08]	[.014, .015]	[.013, .014]	[.014, .015]
Age (months) squared	.0007***	.00072***	.00073***	-.000049***	-.000049***	-.000053***
	[.00066, .00075]	[.00068, .00077]	[.00063, .00083]	[-.00005, -.000048]	[-.000051, -.000048]	[-.000055, -.000052]
Female	.16***	.16***	.17***	-.02***	-.022***	-.027***
	[.14, .17]	[.14, .17]	[.13, .21]	[-.023, -.018]	[-.024, -.02]	[-.031, -.024]
Mother's age at birth (years)	.064***	.047***		.0033***	-.0017*	
	[.055, .074]	[.037, .057]		[.0013, .0052]	[-.0034, 3.8e-06]	
Mother's age at birth (years) squared	-.00069***	-.0005***		-.000054***	.000021	
	[-.00085, -.00054]	[-.00067, -.00032]		[-.000089, -.000019]	[-.000011, .000052]	
Mother's education (years)	.073***	.035***		.024***	.0091***	
	[.071, .076]	[.032, .038]		[.023, .024]	[.0087, .0095]	
Number of siblings	.18***	.2***		-.016***	-.0049***	
	[.16, .2]	[.18, .22]		[-.018, -.015]	[-.006, -.0038]	
Twin	-.58***	-.62***	-.7***	.025***	.0095**	.011
	[-.64, -.53]	[-.68, -.56]	[-.88, -.53]	[.017, .033]	[.0018, .017]	[-.003, .026]
Constant	-1.7***	-1.8***	-1.7***	.79***	.76***	.75***
	[-1.7, -1.7]	[-1.8, -1.8]	[-1.8, -1.7]	[.79, .8]	[.75, .76]	[.75, .76]
R squared	0.093	0.267	0.699	0.222	0.433	0.706
Observations	199,417	196,543	57,814	442,405	441,159	323,830

Notes: ***p<0.01; **p<0.05; *p<0.1. Linear regression coefficients are shown. Postneonatal mortality rate was centered around percentile 25 (considering the pooled samples for both outcomes) and all covariates were mean-centered (using means for all valid observations in each analysis): therefore, the constant shows the mean outcome when covariates were at their means and postneonatal mortality rate was at percentile 25, and the coefficient of the linear term for postneonatal mortality rate shows the marginal effect at percentile 25. 95% confidence intervals (shown in brackets) and p-values were adjusted for clustering within primary sampling units. Postneonatal mortality rate per 100 person-years within a 50 kilometer radius was linked to the period of infancy.

Figure S22. Marginal effects of postneonatal mortality rate on height-for-age across the distribution of postneonatal mortality rate: Postneonatal mortality rate converted to natural log scale

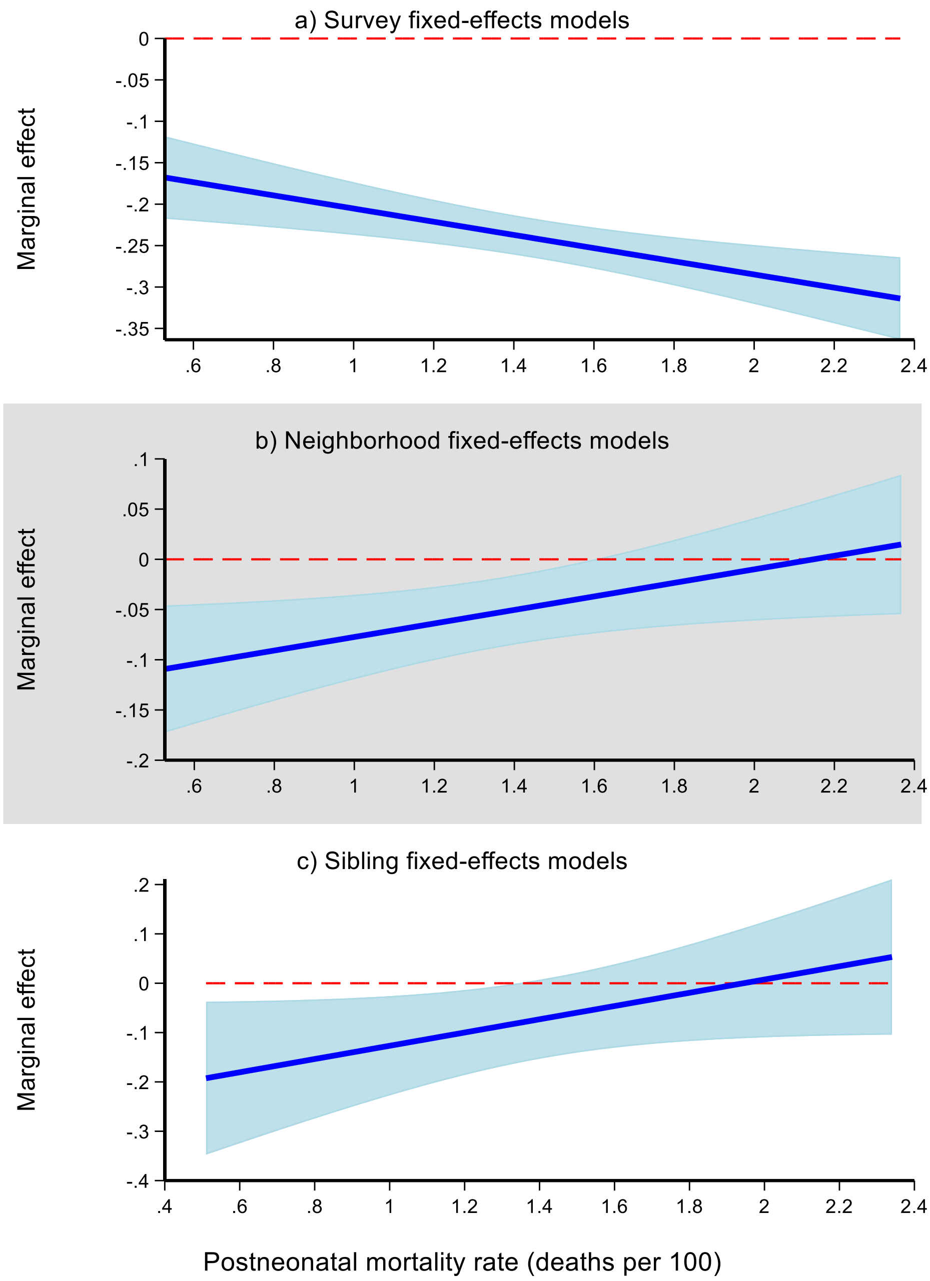

Notes: The values on the y-axis indicate marginal effects: that is, change in the outcome for a single death increase in postneonatal mortality rate per 100 person-years. Since the relationship was nonlinear, the marginal effects vary across the distribution of postneonatal mortality rate. The marginal effects were obtained using the partial derivative of the regression equation with respect to postneonatal mortality rate. The plot was restricted to postneonatal mortality rate between the 5th and 95th percentile (considering the pooled samples for both outcomes). 95% confidence intervals adjusted for clustering within primary sampling units are shown. Postneonatal mortality rate per 100 person-years within a 50 kilometer radius was linked to the period of infancy.

Figure S23. Marginal effects of postneonatal mortality rate on school attendance across the distribution of postneonatal mortality rate: Postneonatal mortality rate converted to natural log scale

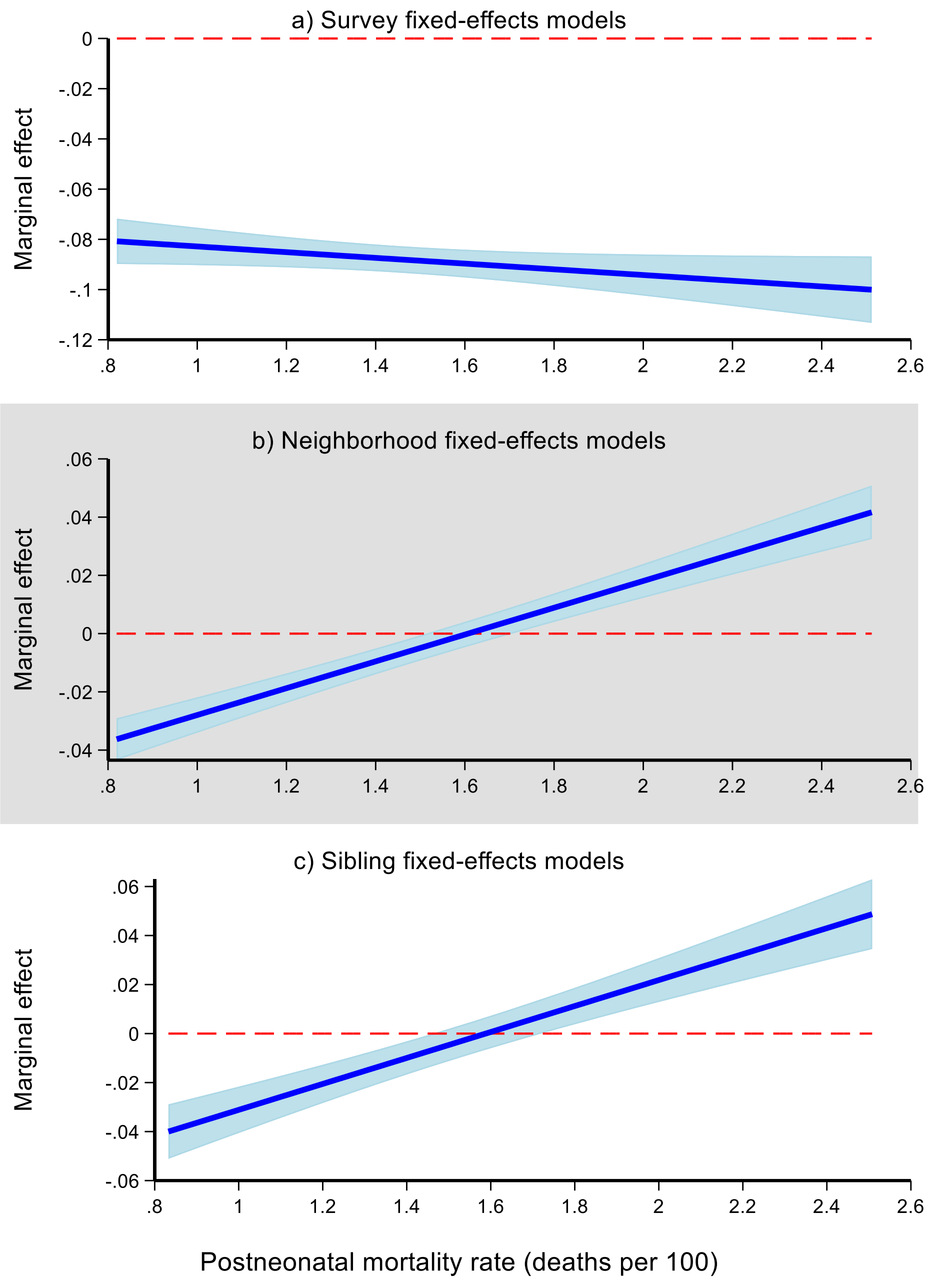

Notes: The values on the y-axis indicate marginal effects: that is, change in the outcome for a single death increase in postneonatal mortality rate per 100 person-years. Since the relationship was nonlinear, the marginal effects vary across the distribution of postneonatal mortality rate. The marginal effects were obtained using the partial derivative of the regression equation with respect to postneonatal mortality rate. The plot was restricted to postneonatal mortality rate between the 5th and 95th percentile (considering the pooled samples for both outcomes). 95% confidence intervals adjusted for clustering within primary sampling units are shown. Postneonatal mortality rate per 100 person-years within a 50 kilometer radius was linked to the period of infancy.

Table S28. Marginal effects of postneonatal mortality rate on height-for-age and school attendance at different percentiles of postneonatal mortality rate: Postneonatal mortality rate converted to natural log scale

Outcome->	Height-for-age z-score			School attendance		
Fixed-effects->	Survey	Neighborhood	Sibling	Survey	Neighborhood	Sibling
Percentiles of postneonatal mortality rate (per 100)						
p1 (0.3)	-.14***	-.13***	-.24**	-.076***	-.055***	-.061***
	[-.2, -.074]	[-.22, -.051]	[-.43, -.039]	[-.089, -.063]	[-.065, -.045]	[-.076, -.045]
p5 (0.7)	-.17***	-.11***	-.19**	-.081***	-.036***	-.04***
	[-.22, -.12]	[-.17, -.046]	[-.35, -.038]	[-.09, -.072]	[-.043, -.029]	[-.051, -.029]
p10 (0.9)	-.19***	-.094***	-.16**	-.083***	-.027***	-.03***
	[-.23, -.14]	[-.15, -.042]	[-.29, -.035]	[-.09, -.076]	[-.033, -.021]	[-.039, -.02]
p25 (1.3)	-.21***	-.07***	-.12**	-.087***	-.011***	-.011***
	[-.24, -.19]	[-.11, -.031]	[-.21, -.022]	[-.092, -.082]	[-.016, -.0067]	[-.018, -.0042]
p50 (1.7)	-.25***	-.041**	-.059	-.091***	.0069***	.0094**
	[-.27, -.22]	[-.077, -.0051]	[-.14, .023]	[-.098, -.085]	[.0022, .012]	[.0021, .017]
p75 (2.0)	-.28***	-.015	-.0066	-.095***	.023***	.027***
	[-.31, -.25]	[-.063, .033]	[-.11, .1]	[-.1, -.086]	[.016, .029]	[.017, .037]
p90 (2.3)	-.3***	.0037	.031	-.098***	.035***	.041***
	[-.34, -.26]	[-.057, .064]	[-.11, .17]	[-.11, -.087]	[.027, .043]	[.028, .053]
p95 (2.5)	-.31***	.015	.053	-.1***	.042***	.049***
	[-.36, -.26]	[-.055, .084]	[-.1, .21]	[-.11, -.087]	[.032, .051]	[.034, .063]
p99 (2.8)	-.34***	.036	.095	-.1***	.054***	.063***
	[-.4, -.28]	[-.051, .12]	[-.1, .29]	[-.12, -.087]	[.043, .066]	[.046, .081]

Notes: ***p<0.01; **p<0.05; *p<0.1. Change in outcome for a single increase in postneonatal mortality rate per 100 person-years are shown at different percentiles of postneonatal mortality rate. Since the relationship was nonlinear, the marginal effects vary across the distribution of postneonatal mortality rate. The marginal effects were obtained using the partial derivative of the regression equation with respect to postneonatal mortality rate. The level of postneonatal mortality rate per 100 at each of the percentile is shown in parentheses (considering the pooled samples for both outcomes). 95% confidence intervals adjusted for clustering within primary sampling units are shown in brackets. Postneonatal mortality rate per 100 person-years within a 50 kilometer radius was linked to the period of infancy.

SUPPLEMENT 5: Full results by sex and mother's education attainment

Table S29. Results from linear regression models: Postneonatal mortality rate interacted with having a mother with education

Outcome->	Height-for-age z-score			School attendance		
Fixed-effects->	Survey	Neighborhood	Sibling	Survey	Neighborhood	Sibling
Independent variables						
Postneonatal mortality rate (per 100)	-.068***	-.017***	-.038**	-.03***	-.0012	.0014
	[-.077, -.058]	[-.029, -.0049]	[-.07, -.006]	[-.032, -.028]	[-.0027, .00038]	[-.0013, .004]
Postneonatal mortality rate (per 100) squared	.0023***	.0012**	.0029***	.0013***	.00024***	.000094
	[.0015, .0031]	[.00026, .0021]	[.00081, .005]	[.0011, .0015]	[.00012, .00037]	[-.00012, .00031]
Mother had education×Postneonatal mortality rate (per 100)	.016***	.0044	.033	.017***	-.0013	-.0046***
	[.0046, .027]	[-.0086, .017]	[-.016, .082]	[.015, .019]	[-.0029, .00031]	[-.0078, -.0015]
Mother had education×Postneonatal mortality rate (per 100) squared	-.00085	-.00045	-.0029	-.00063***	.00032***	.00049***
	[-.0021, .00041]	[-.0019, .00099]	[-.0077, .002]	[-.00082, -.00043]	[.00016, .00049]	[.00019, .00079]
Mothers had education	-.09***	-.091***		.099***	.036***	
	[-.12, -.058]	[-.13, -.054]		[.092, .1]	[.031, .041]	
Firstborn	.22***	.2***	.24***	.0029	.00028	-.015***
	[.19, .25]	[.17, .24]	[.14, .33]	[-.0014, .0073]	[-.0038, .0043]	[-.022, -.0082]
Birth interval (months)	.0047***	.0047***	.007***	.00012***	.000052	-.00024***
	[.0043, .0051]	[.0042, .0052]	[.005, .009]	[.000052, .00019]	[-.000011, .00011]	[-.00038, -.000098]
Birth order	-.22***	-.23***	-1.1***	.0095***	.00065	-.015***
	[-.24, -.2]	[-.25, -.21]	[-1.2, -.98]	[.0079, .011]	[-.00076, .0021]	[-.021, -.0094]
Age (months)	-.055***	-.058***	-.087***	.015***	.014***	.014***
	[-.058, -.052]	[-.061, -.054]	[-.095, -.079]	[.014, .015]	[.013, .014]	[.014, .015]
Age (months) squared	.0007***	.00072***	.00073***	-.000049***	-.000049***	-.000053***
	[.00066, .00074]	[.00068, .00077]	[.00063, .00083]	[-.00005, -.000048]	[-.000051, -.000048]	[-.000055, -.000052]
Female	.16***	.16***	.17***	-.021***	-.022***	-.028***
	[.14, .17]	[.14, .17]	[.13, .21]	[-.023, -.018]	[-.024, -.02]	[-.031, -.024]
Mother's age at birth (years)	.064***	.046***		.0052***	-.0014	
	[.054, .073]	[.035, .056]		[.0033, .007]	[-.0031, .00035]	
Mother's age at birth (years) squared	-.00069***	-.00049***		-.000071***	.000019	
	[-.00085, -.00053]	[-.00066, -.00031]		[-.00011, -.000036]	[-.000013, .00005]	
Mother's education (years)	.08***	.043***		.012***	.0058***	
	[.076, .083]	[.038, .047]		[.012, .013]	[.0053, .0062]	
Number of siblings	.18***	.2***		-.017***	-.0051***	
	[.16, .2]	[.19, .22]		[-.018, -.016]	[-.0061, -.004]	
Twin	-.58***	-.62***	-.7***	.025***	.0099**	.011
	[-.64, -.53]	[-.68, -.56]	[-.88, -.52]	[.017, .034]	[.0022, .018]	[-.0031, .026]
Constant	-1.6***	-1.7***	-1.7***	.75***	.74***	.76***
	[-1.7, -1.6]	[-1.8, -1.7]	[-1.8, -1.7]	[.75, .76]	[.74, .74]	[.75, .76]
R squared	0.094	0.267	0.699	0.231	0.433	0.706
Observations	199,417	196,543	57,814	442,405	441,159	323,830

Notes: ***p<0.01; **p<0.05; *p<0.1. Linear regression coefficients are shown. Postneonatal mortality rate was centered around percentile 25 (considering the pooled samples for both outcomes) and all covariates were mean-centered (using means for all valid observations in each analysis): therefore, the constant shows the mean outcome when covariates were at their means and postneonatal mortality rate was at percentile 25, and the coefficient of the linear term for postneonatal mortality rate shows the marginal effect at percentile 25. 95% confidence intervals (shown in brackets) and p-values were adjusted for clustering within primary sampling units. Postneonatal mortality rate per 100 person-years within a 50 kilometer radius was linked to the period of infancy.

Figure S24. Marginal effects of postneonatal mortality rate on height-for-age across the distribution of postneonatal mortality rate: by maternal education

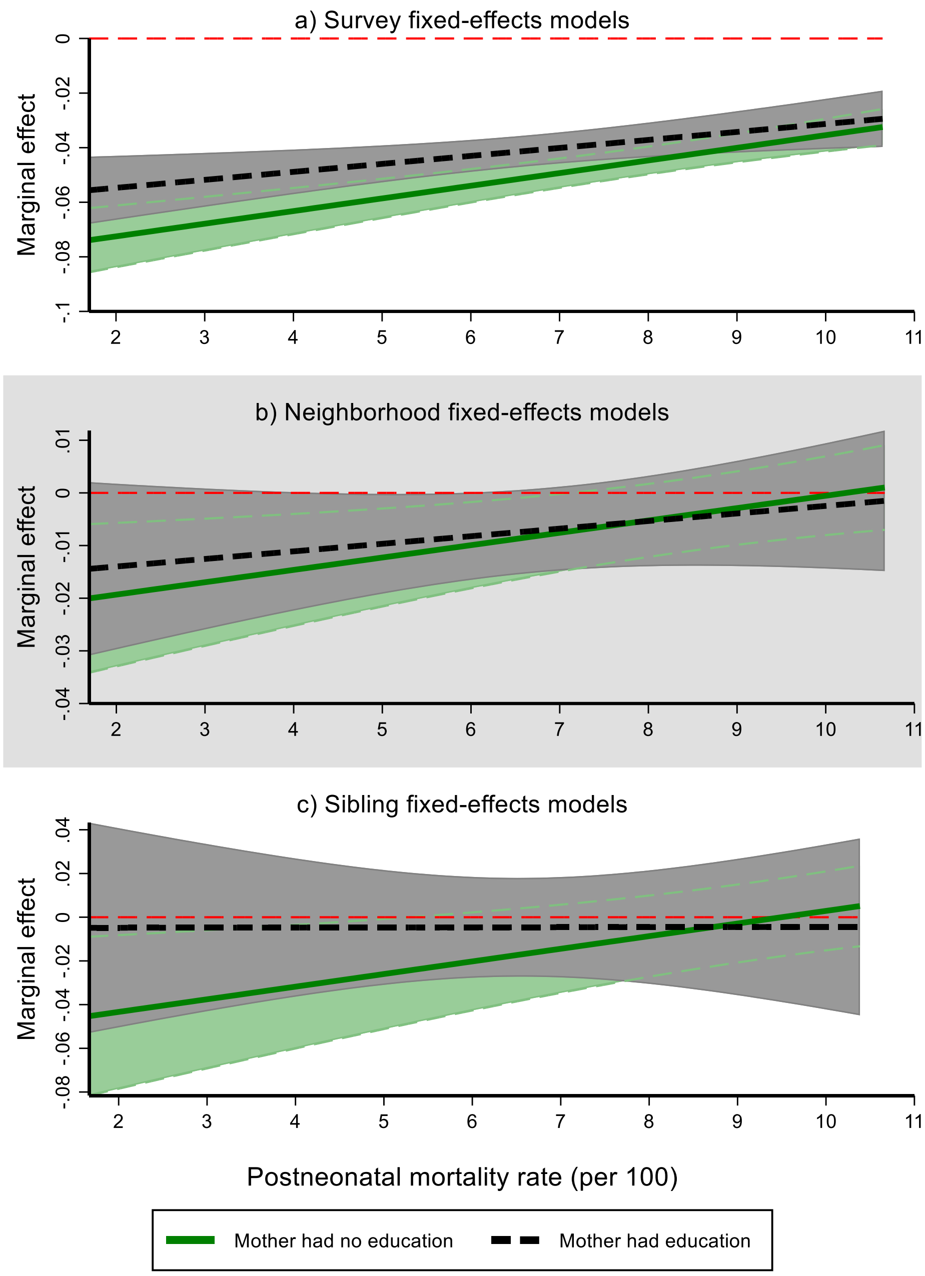

Notes: The values on the y-axis indicate marginal effects: that is, change in the outcome for a single death increase in postneonatal mortality rate per 100 person-years. Since the relationship was nonlinear, the marginal effects vary across the distribution of postneonatal mortality rate. The marginal effects were obtained using the partial derivative of the regression equation with respect to postneonatal mortality rate. The plot was restricted to postneonatal mortality rate between the 5th and 95th percentile (considering the pooled samples for both outcomes). 95% confidence intervals adjusted for clustering within primary sampling units are shown. Postneonatal mortality rate per 100 person-years within a 50 kilometer radius was linked to the period of infancy.

Figure S25. Marginal effects of postneonatal mortality rate on school attendance across the distribution of postneonatal mortality rate: by maternal education

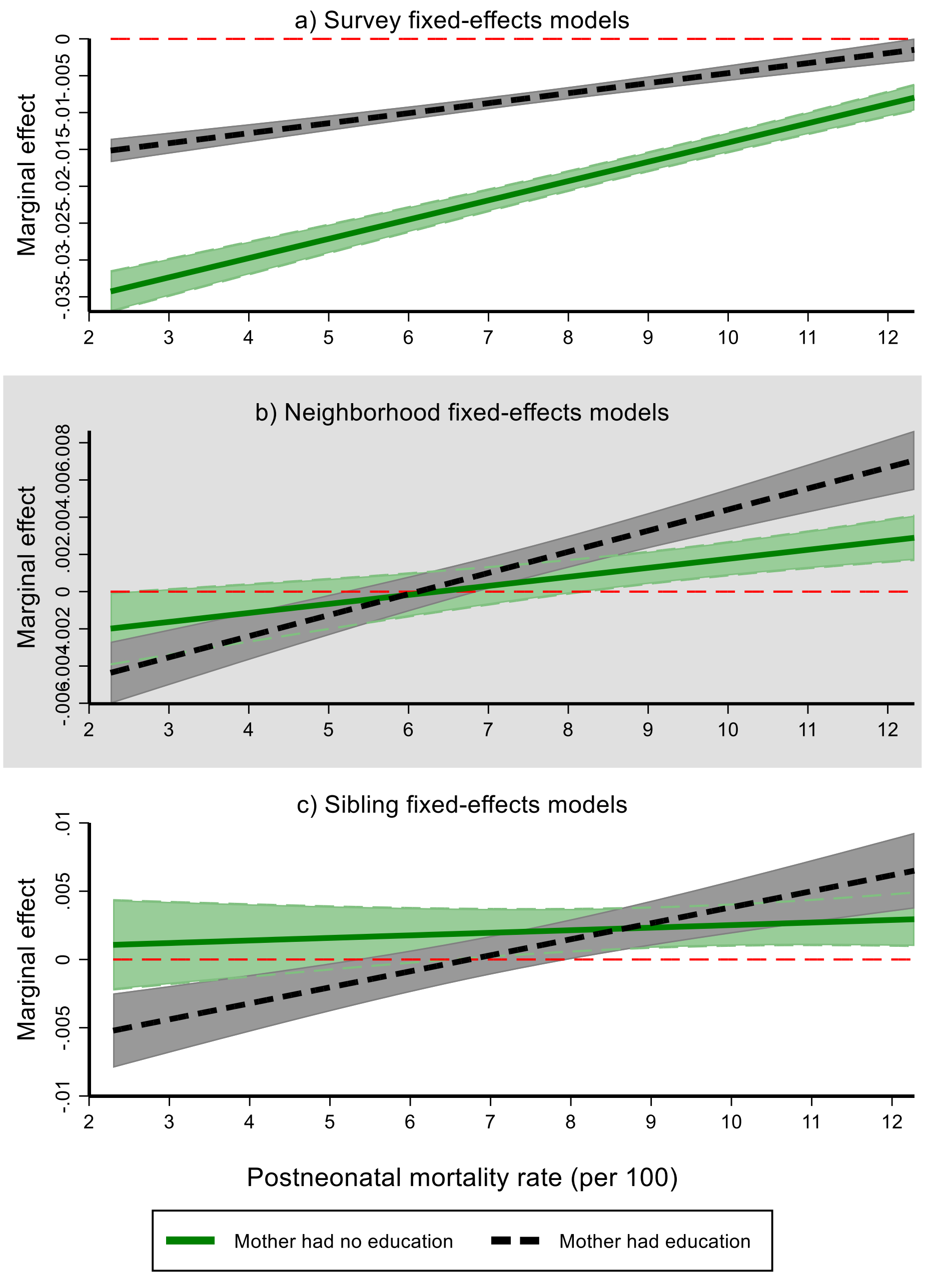

Notes: The values on the y-axis indicate marginal effects: that is, change in the outcome for a single death increase in postneonatal mortality rate per 100 person-years. Since the relationship was nonlinear, the marginal effects vary across the distribution of postneonatal mortality rate. The marginal effects were obtained using the partial derivative of the regression equation with respect to postneonatal mortality rate. The plot was restricted to postneonatal mortality rate between the 5th and 95th percentile (considering the pooled samples for both outcomes). 95% confidence intervals adjusted for clustering within primary sampling units are shown. Postneonatal mortality rate per 100 person-years within a 50 kilometer radius was linked to the period of infancy.

Table S30. Marginal effects of postneonatal mortality rate on height-for-age and school attendance at different percentiles of postneonatal mortality rate: by maternal education

Outcome->	Height-for-age z-score			School attendance		
Fixed-effects->	Survey	Neighborhood	Sibling	Survey	Neighborhood	Sibling
Percentiles of postneonatal mortality rate (per 100)						
Mother had no education						
p1 (1.4)	-.076***	-.021***	-.048**	-.036***	-.0023**	.00093
	[-.089, -.064]	[-.036, -.0063]	[-.086, -.0099]	[-.039, -.033]	[-.0044, -.00025]	[-.0026, .0045]
p5 (2.0)	-.074***	-.02***	-.045**	-.034***	-.002**	.0011
	[-.086, -.062]	[-.034, -.0059]	[-.082, -.0089]	[-.037, -.032]	[-.0039, -.000062]	[-.0022, .0043]
p10 (2.5)	-.072***	-.019***	-.043**	-.033***	-.0017*	.0012
	[-.083, -.061]	[-.033, -.0056]	[-.078, -.0081]	[-.036, -.03]	[-.0036, .000067]	[-.0019, .0043]
p25 (3.6)	-.068***	-.017***	-.038**	-.03***	-.0012	.0014
	[-.077, -.058]	[-.029, -.0049]	[-.07, -.006]	[-.032, -.028]	[-.0027, .00038]	[-.0013, .004]
p50 (5.4)	-.06***	-.013***	-.029**	-.025***	-.00027	.0017*
	[-.068, -.053]	[-.023, -.0033]	[-.055, -.0021]	[-.027, -.023]	[-.0015, .00092]	[-.00031, .0038]
p75 (7.8)	-.05***	-.0079**	-.016	-.019***	.00086*	.0022***
	[-.056, -.045]	[-.015, -.00047]	[-.037, .0044]	[-.02, -.018]	[-.000028, .0018]	[.00063, .0037]
p90 (10.2)	-.04***	-.0028	-.0041	-.013***	.002***	.0026***
	[-.045, -.035]	[-.0098, .0042]	[-.022, .014]	[-.014, -.011]	[.0011, .003]	[.001, .0042]
p95 (11.9)	-.032***	.001	.0051	-.008***	.0029***	.0029***
	[-.039, -.026]	[-.007, .0091]	[-.013, .024]	[-.0097, -.0062]	[.0017, .0041]	[.00099, .0049]
p99 (15.8)	-.015**	.01	.027**	.0023	.0048***	.0037**
	[-.026, -.0027]	[-.0031, .023]	[.000041, .054]	[-.0006, .0051]	[.0028, .0068]	[.00039, .007]
Mother had education						
p1 (1.4)	-.057***	-.015*	-.0049	-.016***	-.0052***	-.0061***
	[-.07, -.044]	[-.033, .0026]	[-.057, .047]	[-.018, -.014]	[-.0071, -.0034]	[-.0091, -.0031]
p5 (2.0)	-.056***	-.014*	-.0049	-.015***	-.0044***	-.0052***
	[-.068, -.043]	[-.031, .002]	[-.053, .043]	[-.017, -.014]	[-.006, -.0027]	[-.0079, -.0025]
p10 (2.5)	-.054***	-.014*	-.0048	-.014***	-.0038***	-.0046***
	[-.066, -.043]	[-.029, .0016]	[-.05, .04]	[-.016, -.013]	[-.0053, -.0023]	[-.0071, -.0021]
p25 (3.6)	-.052***	-.012*	-.0048	-.013***	-.0025***	-.0032***
	[-.061, -.042]	[-.026, .0008]	[-.043, .034]	[-.014, -.012]	[-.0038, -.0012]	[-.0053, -.0012]
p50 (5.4)	-.047***	-.01**	-.0047	-.01***	-.00036	-.0011
	[-.054, -.04]	[-.02, -.00013]	[-.033, .024]	[-.011, -.0094]	[-.0013, .00061]	[-.0026, .0005]
p75 (7.8)	-.041***	-.007*	-.0046	-.0072***	.0023***	.0016**
	[-.046, -.035]	[-.015, .00095]	[-.027, .018]	[-.008, -.0063]	[.0014, .0032]	[.00017, .0031]
p90 (10.2)	-.034***	-.0038	-.0045	-.0038***	.0051***	.0045***
	[-.042, -.026]	[-.014, .0062]	[-.034, .025]	[-.005, -.0027]	[.0039, .0063]	[.0024, .0066]
p95 (11.9)	-.029***	-.0015	-.0044	-.0015*	.0071***	.0065***
	[-.04, -.019]	[-.015, .012]	[-.045, .036]	[-.003, .00005]	[.0055, .0086]	[.0037, .0093]
p99 (15.8)	-.018**	.0041	-.0042	.0038***	.011***	.011***

Outcome->	Height-for-age z-score			School attendance		
	[-.036, -.00011]	[-.019, .027]	[-.075, .066]	[.0014, .0063]	[.0089, .014]	[.0066, .016]

Notes: ***p<0.01; **p<0.05; *p<0.1. Change in outcome for a single increase in postneonatal mortality rate per 100 person-years are shown at different percentiles of postneonatal mortality rate. Since the relationship was nonlinear, the marginal effects vary across the distribution of postneonatal mortality rate. The marginal effects were obtained using the partial derivative of the regression equation with respect to postneonatal mortality rate. The level of postneonatal mortality rate per 100 at each of the percentile is shown in parentheses (considering the pooled samples for both outcomes). 95% confidence intervals adjusted for clustering within primary sampling units are shown in brackets. Postneonatal mortality rate per 100 person-years within a 50 kilometer radius was linked to the period of infancy.

Table S31. Results from linear regression models: Postneonatal mortality rate interacted with being female

Outcome->	Height-for-age z-score			School attendance		
Fixed-effects->	Survey	Neighborhood	Sibling	Survey	Neighborhood	Sibling
Independent variables						
Postneonatal mortality rate (per 100)	-.056***	-.011*	-.025*	-.017***	.0025***	.0037***
	[-.065, -.047]	[-.022, .001]	[-.052, .0022]	[-.019, -.015]	[.0011, .0038]	[.0016, .0057]
Postneonatal mortality rate (per 100) squared	.0017***	.00085*	.002*	.00063***	.00015**	.00011
	[.00089, .0025]	[-.0001, .0018]	[-.00018, .0041]	[.00048, .00078]	[.000028, .00028]	[-.000089, .00031]
Female×Postneonatal mortality rate (per 100)	-.0089**	-.0095**	-.0023	-.0076***	-.0084***	-.0089***
	[-.018, -.00032]	[-.019, -.00016]	[-.029, .024]	[-.009, -.0063]	[-.0096, -.0071]	[-.011, -.0068]
Female×Postneonatal mortality rate (per 100) squared	.00036	.00041	.00029	.00036***	.00037***	.00033***
	[-.00054, .0013]	[-.00054, .0014]	[-.0025, .0031]	[.00021, .00051]	[.00023, .00051]	[.000089, .00057]
Firstborn	.22***	.2***	.24***	.0009	-.00006	-.015***
	[.19, .25]	[.17, .24]	[.14, .33]	[-.0035, .0053]	[-.0041, .004]	[-.022, -.0085]
Birth interval (months)	.0047***	.0047***	.007***	.00016***	.000062*	-.00024***
	[.0043, .0051]	[.0042, .0051]	[.005, .009]	[.000091, .00023]	[-7.4e-07, .00012]	[-.00038, -.000096]
Birth order	-.22***	-.23***	-1.1***	.01***	.00078	-.015***
	[-.24, -.2]	[-.25, -.21]	[-1.2, -.98]	[.0088, .012]	[-.00063, .0022]	[-.021, -.0094]
Age (months)	-.055***	-.058***	-.087***	.015***	.014***	.015***
	[-.058, -.052]	[-.061, -.054]	[-.095, -.08]	[.014, .015]	[.013, .014]	[.014, .015]
Age (months) squared	.0007***	.00072***	.00073***	-.000049***	-.000049***	-.000053***
	[.00066, .00074]	[.00068, .00077]	[.00063, .00083]	[-.00005, -.000048]	[-.000051, -.000048]	[-.000055, -.000052]
Female	.17***	.17***	.17***	-.0078***	-.0076***	-.011***
	[.16, .19]	[.15, .19]	[.12, .22]	[-.011, -.005]	[-.01, -.005]	[-.016, -.007]
Mother's age at birth (years)	.065***	.047***		.0033***	-.0017*	
	[.055, .074]	[.037, .057]		[.0014, .0052]	[-.0034, .000015]	
Mother's age at birth (years) squared	-.0007***	-.0005***		-.000054***	.000021	
	[-.00085, -.00054]	[-.00067, -.00032]		[-.00009, -.000019]	[-.000011, .000052]	
Mother's education (years)	.073***	.035***		.024***	.0091***	
	[.071, .076]	[.032, .038]		[.023, .024]	[.0087, .0095]	
Number of siblings	.18***	.2***		-.016***	-.0049***	
	[.16, .2]	[.18, .22]		[-.018, -.015]	[-.0059, -.0038]	
Twin	-.58***	-.62***	-.7***	.025***	.0096**	.011
	[-.64, -.53]	[-.68, -.56]	[-.88, -.52]	[.017, .033]	[.0019, .017]	[-.003, .026]
Constant	-1.8***	-1.9***	-1.8***	.8***	.76***	.76***
	[-1.8, -1.8]	[-1.9, -1.8]	[-1.9, -1.8]	[.8, .81]	[.76, .77]	[.76, .77]
R squared	0.093	0.267	0.699	0.223	0.433	0.706
Observations	199,417	196,543	57,814	442,405	441,159	323,830

Notes: ***p<0.01; **p<0.05; *p<0.1. Linear regression coefficients are shown. Postneonatal mortality rate was centered around percentile 25 (considering the pooled samples for both outcomes) and all covariates were mean-centered (using means for all valid observations in each analysis): therefore, the constant shows the mean outcome when covariates were at their means and postneonatal mortality rate was at percentile 25, and the coefficient of the linear term for postneonatal mortality

rate shows the marginal effect at percentile 25. 95% confidence intervals (shown in brackets) and p-values were adjusted for clustering within primary sampling units. Postneonatal mortality rate per 100 person-years within a 50 kilometer radius was linked to the period of infancy.

Figure S26. Marginal effects of postneonatal mortality rate on height-for-age across the distribution of postneonatal mortality rate: by sex

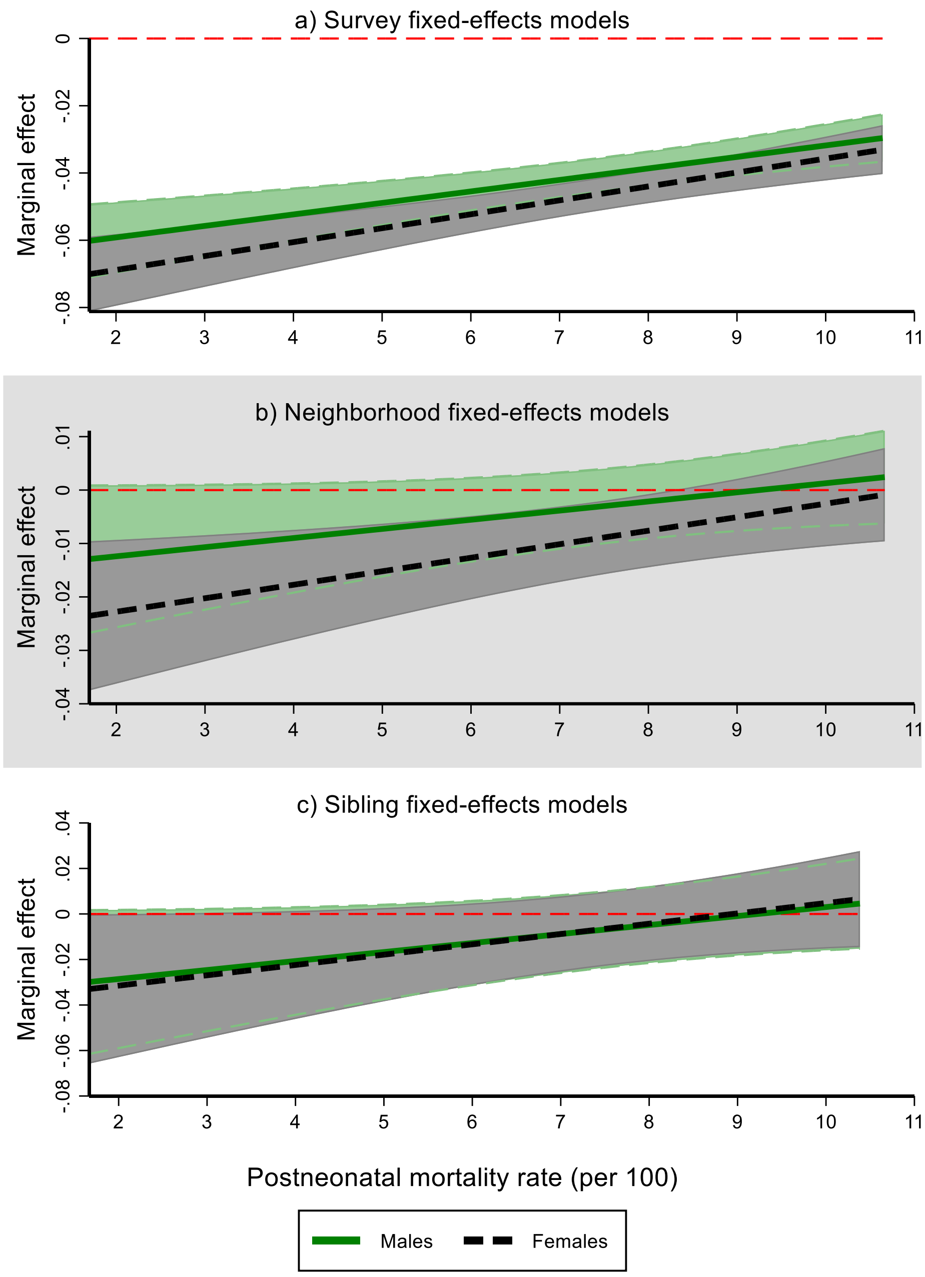

Notes: The values on the y-axis indicate marginal effects: that is, change in the outcome for a single death increase in postneonatal mortality rate per 100 person-years. Since the relationship was nonlinear, the marginal effects vary across the distribution of postneonatal mortality rate. The marginal effects were obtained using the partial derivative of the regression equation with respect to postneonatal mortality rate. The plot was restricted to postneonatal mortality rate between the 5th and 95th percentile (considering the pooled samples for both outcomes). 95% confidence intervals adjusted for clustering within primary sampling units are shown. Postneonatal mortality rate per 100 person-years within a 50 kilometer radius was linked to the period of infancy.

Figure S27. Marginal effects of postneonatal mortality rate on school attendance across the distribution of postneonatal mortality rate: by sex

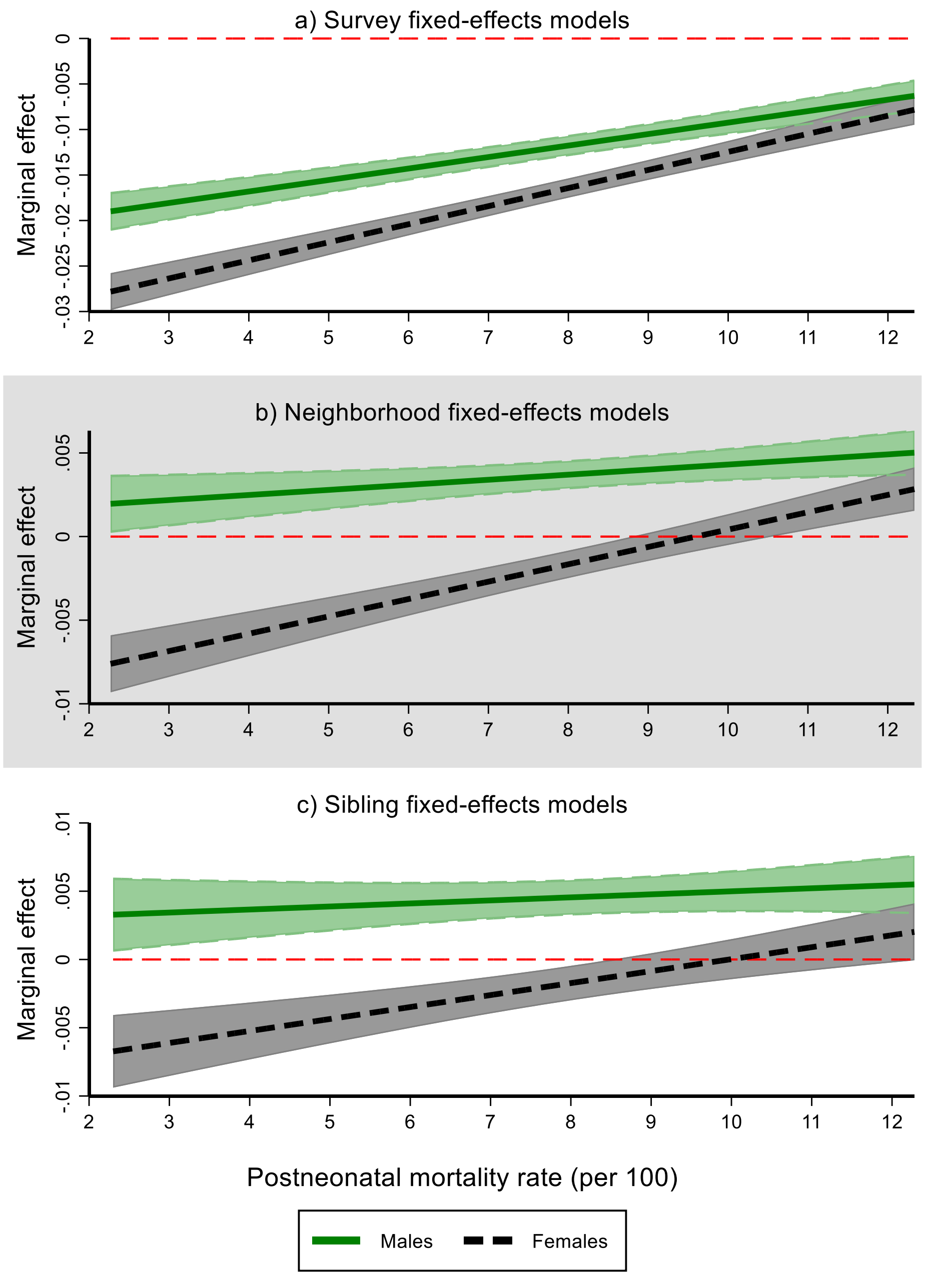

Notes: The values on the y-axis indicate marginal effects: that is, change in the outcome for a single death increase in postneonatal mortality rate per 100 person-years. Since the relationship was nonlinear, the marginal effects vary across the distribution of postneonatal mortality rate. The marginal effects were obtained using the partial derivative of the regression equation with respect to postneonatal mortality rate. The plot was restricted to postneonatal mortality rate between the 5th and 95th percentile (considering the pooled samples for both outcomes). 95% confidence intervals adjusted for clustering within primary sampling units are shown. Postneonatal mortality rate per 100 person-years within a 50 kilometer radius was linked to the period of infancy.

Table S32. Marginal effects of postneonatal mortality rate on height-for-age and school attendance at different percentiles of postneonatal mortality rate: by sex

Outcome->	Height-for-age z-score			School attendance		
Fixed-effects->	Survey	Neighborhood	Sibling	Survey	Neighborhood	Sibling
Percentiles of postneonatal mortality rate (per 100)						
Boys						
p1 (1.4)	-.062***	-.014*	-.032*	-.02***	.0017*	.0031**
	[-.074, -.05]	[-.028, .00086]	[-.065, .0016]	[-.022, -.018]	[-.0001, .0036]	[.00021, .006]
p5 (2.0)	-.06***	-.013*	-.03*	-.019***	.002**	.0033**
	[-.071, -.049]	[-.027, .00088]	[-.062, .0017]	[-.021, -.017]	[.00028, .0036]	[.00064, .0059]
p10 (2.5)	-.059***	-.012*	-.028*	-.018***	.0021***	.0034***
	[-.069, -.048]	[-.025, .00091]	[-.059, .0018]	[-.02, -.016]	[.00054, .0037]	[.00093, .0059]
p25 (3.6)	-.056***	-.011*	-.025*	-.017***	.0025***	.0037***
	[-.065, -.047]	[-.022, .001]	[-.052, .0022]	[-.019, -.015]	[.0011, .0038]	[.0016, .0057]
p50 (5.4)	-.05***	-.0078*	-.019*	-.015***	.003***	.0041***
	[-.057, -.043]	[-.017, .0015]	[-.041, .0035]	[-.016, -.013]	[.002, .004]	[.0025, .0056]
p75 (7.8)	-.043***	-.0041	-.01	-.012***	.0037***	.0046***
	[-.048, -.037]	[-.011, .0031]	[-.028, .0073]	[-.013, -.011]	[.0029, .0046]	[.0033, .0058]
p90 (10.2)	-.035***	-.00035	-.0017	-.0085***	.0045***	.0051***
	[-.041, -.03]	[-.0076, .0069]	[-.019, .015]	[-.0098, -.0072]	[.0035, .0055]	[.0035, .0067]
p95 (11.9)	-.03***	.0024	.0046	-.0063***	.005***	.0055***
	[-.037, -.023]	[-.0063, .011]	[-.015, .024]	[-.008, -.0046]	[.0037, .0063]	[.0034, .0076]
p99 (15.8)	-.016***	.009	.02	-.0014	.0062***	.0064***
	[-.029, -.004]	[-.0054, .023]	[-.012, .052]	[-.0041, .0014]	[.004, .0084]	[.0029, .0098]
Girls						
p1 (1.4)	-.072***	-.025***	-.035**	-.029***	-.0084***	-.0074***
	[-.084, -.06]	[-.04, -.01]	[-.07, -.00033]	[-.032, -.027]	[-.01, -.0065]	[-.01, -.0045]
p5 (2.0)	-.07***	-.024***	-.033**	-.028***	-.0076***	-.0067***
	[-.081, -.059]	[-.038, -.0096]	[-.066, -.00018]	[-.03, -.026]	[-.0093, -.0059]	[-.0094, -.0041]
p10 (2.5)	-.068***	-.022***	-.031**	-.027***	-.0071***	-.0063***
	[-.079, -.058]	[-.036, -.0092]	[-.062, -.00003]	[-.029, -.025]	[-.0087, -.0055]	[-.0088, -.0038]
p25 (3.6)	-.065***	-.02***	-.027*	-.025***	-.0059***	-.0053***
	[-.074, -.055]	[-.032, -.0084]	[-.055, .00046]	[-.026, -.023]	[-.0073, -.0045]	[-.0074, -.0032]
p50 (5.4)	-.058***	-.016***	-.02*	-.021***	-.0039***	-.0036***
	[-.065, -.051]	[-.025, -.0067]	[-.042, .0019]	[-.022, -.02]	[-.005, -.0029]	[-.0052, -.0021]
p75 (7.8)	-.049***	-.011***	-.01	-.016***	-.0015***	-.0016**
	[-.054, -.044]	[-.018, -.0034]	[-.027, .0065]	[-.017, -.015]	[-.0023, -.00069]	[-.0029, -.00034]
p90 (10.2)	-.04***	-.005	-.00069	-.011***	.001**	.00052
	[-.045, -.034]	[-.012, .0022]	[-.018, .017]	[-.013, -.01]	[.000032, .002]	[-.0011, .0021]
p95 (11.9)	-.033***	-.00086	.0065	-.0078***	.0028***	.002*
	[-.04, -.026]	[-.0096, .0079]	[-.015, .028]	[-.0095, -.0062]	[.0015, .0041]	[-.000063, .0041]
p99 (15.8)	-.017**	.0089	.024	-.000046	.0069***	.0055***
	[-.03, -.0041]	[-.0058, .024]	[-.012, .06]	[-.0027, .0026]	[.0048, .0091]	[.002, .0089]

Notes: ***p<0.01; **p<0.05; *p<0.1. Change in outcome for a single increase in postneonatal mortality rate per 100 person-years are shown at different percentiles of postneonatal mortality rate. Since the relationship was nonlinear, the marginal effects vary across the distribution of postneonatal mortality rate. The marginal effects were obtained using the partial derivative of the regression equation with respect to postneonatal mortality rate. The level of postneonatal mortality rate per 100 at each of the percentile is shown in parentheses (considering the pooled samples for both outcomes). 95% confidence intervals adjusted for clustering within primary sampling units are shown in brackets. Postneonatal mortality rate per 100 person-years within a 50 kilometer radius was linked to the period of infancy.
